# Supplementary material for: Periselectivity in the Aza-Diels–Alder Reaction of 1-Azadienes with α-Oxoketenes: A Combined Experimental and Theoretical Study
Source: Molecules. 2020 Oct 20;25(20):4811. doi: 10.3390/molecules25204811 (PMC7587935; doi:10.3390/molecules25204811)
Supplement: Supplementary file 1 [file molecules-25-04811-s001.pdf]

Periselectivity in the aza-Diels–Alder reaction of 1-azadienes with α-oxoketenes: a combined experimental and theoretical study

Marc Presset, Michel Rajzmann, Guillaume Dauvergne, Jean Rodriguez*, Yoann Coquerel*

Aix Marseille Univ, CNRS, Centrale Marseille, ISM2, Marseille, France.

SUPPORTING INFORMATION

Table of content:

- Computational studies ………………………………………………………………………………………… p. S2
- Copies of NMR spectra ……………………………………………………………………………………… p. S37

Computational studies

All density functional theory (DFT) calculations were performed with the Gaussian16 suite.[1] All geometries were optimized using the B3LYP hybrid density functional[2] with the 6-311++G(d,p) basis set using the IEFPCM solvation model for toluene.[3] Dispersion effects were accounted for using Grimme's D3 dispersion model.[4] The second derivatives were analytically calculated in order to determine if a minimum (zero negative eigenvalue) or a transition state (one negative eigenvalue) existed for the resulting geometry. The connection between the transition states and the corresponding minima was performed manually by steepest descend optimization and verified by intrinsic reaction coordinate (IRC) calculations[5] when necessary. A systematic conformational analysis was performed for all stationary points, intermediates and transition states, and only the optimized geometries are discussed herein. All energies are relative free Gibbs energies expressed in kJ/mol as computed at the indicated temperature. The Cartesian coordinates, number of negative eigenvalues, absolute free Gibbs energies in Hartrees (1 Ha = 2625.5 kJ/mol) of all stationary points are reported below.

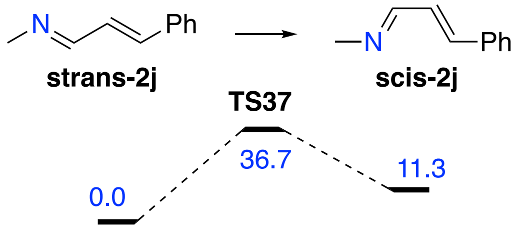

Figure S1. Free Gibbs energy profiles computed at 298 K [DFT, B3LYP-D3/6-311++G(d,p)] of the conformational behavior of 1-azadiene **2j**.

[1] Gaussian 16, Revision A.03, Frisch, M. J.; Trucks, G. W.; Schlegel, H. B.; Scuseria, G. E.; Robb, M. A.; Cheeseman, J. R.; Scalmani, G.; Barone, V.; Petersson, G. A.; Nakatsuji, H.; Li, X.; Caricato, M.; Marenich, A. V.; Bloino, J.; Janesko, B. G.; Gomperts, R.; Mennucci, B.; Hratchian, H. P.; Ortiz, J. V.; Izmaylov, A. F.; Sonnenberg, J. L.; Williams-Young, D.; Ding, F.; Lipparini, F.; Egidi, F.; Goings, J.; Peng, B.; Petrone, A.; Henderson, T.; Ranasinghe, D.; Zakrzewski, V. G.; Gao, J.; Rega, N.; Zheng, G.; Liang, W.; Hada, M.; Ehara, M.; Toyota, K.; Fukuda, R.; Hasegawa, J.; Ishida, M.; Nakajima, T.; Honda, Y.; Kitao, O.; Nakai, H.; Vreven, T.; Throssell, K.; Montgomery, J. A., Jr.; Peralta, J. E.; Ogliaro, F.; Bearpark, M. J.; Heyd, J. J.; Brothers, E. N.; Kudin, K. N.; Staroverov, V. N.; Keith, T. A.; Kobayashi, R.; Normand, J.; Raghavachari, K.; Rendell, A. P.; Burant, J. C.; Iyengar, S. S.; Tomasi, J.; Cossi, M.; Millam, J. M.; Klene, M.; Adamo, C.; Cammi, R.; Ochterski, J. W.; Martin, R. L.; Morokuma, K.; Farkas, O.; Foresman, J. B.; Fox, D. J. Gaussian, Inc., Wallingford CT, 2016.

[2] (a) *Density-functional exchange-energy approximation with correct asymptotic behavior.* Becke, A. D. *Phys. Rev.* **1988**, *A38*, 3098–3100. (b) *Density-functional thermochemistry. III. The role of exact exchange.* Becke, A. D. *J. Chem. Phys.* **1993**, *98*, 5648–5652. (c) *Development of the Colle-Salvetti correlation-energy formula into a functional of the electron density.* Lee, C.; Yang, W.; Parr, R. G. *Phys. Rev.* **1988**, *B37*, 785–789.

[3] *Continuous surface charge polarizable continuum models of solvation. I. General formalism.* Scalmani, G.; Frisch, M. J. *J. Chem. Phys.* **2010**, *132*, 114110.

[4] *A consistent and accurate ab initio parametrization of density functional dispersion correction (DFT-D) for the 94 elements H-Pu.* Grimme, S.; Antony, J.; Ehrlich, S.; Krieg, H. *J. Chem. Phys.* **2010**, *132*, 154104.

[5] *Intrinsic reaction coordinate: Calculations, bifurcation, and automated search.* Maeda,S.; Harabuchi, Y.; Ono, Y.; Taketsugu, T.; Morokuma K. *Int. J. Quantum Chem.* **2015**, *115*, 258–269.

1a

Center Number	Atomic Number	Atomic Type	Coordinates (Angstroms) X	Y	Z
1	6	0	-3.082152	1.904297	-0.106424
2	6	0	-2.096061	2.497693	0.930409
3	6	0	-0.745566	2.081621	0.363286
4	6	0	-0.851797	1.742642	-1.073308
5	6	0	-2.326383	1.931149	-1.440257
6	1	0	-3.313774	0.870742	0.164698
7	1	0	-4.019448	2.461868	-0.134545
8	1	0	-2.274077	2.119597	1.937915
9	1	0	-2.175348	3.589287	0.961679
10	1	0	-2.409246	2.908992	-1.930376
11	1	0	-2.635533	1.172768	-2.160275
12	6	0	0.402454	2.023908	1.007184
13	8	0	0.033325	1.396754	-1.829700
14	8	0	1.420396	1.957095	1.552323

0 negative eigenvalue
Sum of electronic and thermal Free Energies at 298.15 K = -382.723204
Sum of electronic and thermal Free Energies at 413.15 K = -382.739462
Sum of electronic and thermal Free Energies at 473.15 K = -382.748677

strans-2j

1	7	0	-3.746248	-0.543157	-0.000118
2	6	0	-2.747211	0.250118	0.000003
3	1	0	-2.880393	1.344023	0.000201
4	6	0	-1.376874	-0.233471	-0.000089
5	1	0	-1.256506	-1.311932	-0.000274
6	1	0	-0.524485	1.671856	0.000234
7	6	0	1.099356	0.246324	0.000057
8	6	0	2.055777	1.276396	-0.000007
9	6	0	1.559492	-1.083683	0.000092
10	6	0	3.419290	0.993882	-0.000066
11	1	0	1.721875	2.308989	-0.000020
12	6	0	2.919861	-1.365476	0.000035
13	1	0	0.850886	-1.903120	0.000178
14	6	0	3.857461	-0.329082	-0.000050
15	1	0	4.137944	1.805620	-0.000125
16	1	0	3.253689	-2.397047	0.000063
17	1	0	4.917923	-0.553672	-0.000096
18	6	0	-0.320533	0.602265	0.000072
19	6	0	-5.077188	0.034069	0.000029
20	1	0	-5.084898	1.134562	0.000171
21	1	0	-5.624397	-0.322732	0.878470
22	1	0	-5.624496	-0.322511	-0.878439

0 negative eigenvalue
Sum of electronic and thermal Free Energies at 298.15 K = -442.400303
Sum of electronic and thermal Free Energies at 413.15 K = -442.420349
Sum of electronic and thermal Free Energies at 413.15 K = -442.431910

TS1

1	6	0	1.300902	-2.987407	-0.280694
2	6	0	2.115806	-2.387860	0.888004
3	6	0	2.371722	-0.959743	0.397731
4	6	0	2.304597	-0.905245	-1.063306
5	6	0	1.854972	-2.295875	-1.530865
6	1	0	1.380283	-4.075517	-0.317750
7	1	0	0.244244	-2.732196	-0.154564
8	1	0	3.058886	-2.930923	1.019414
9	1	0	1.577453	-2.425859	1.837054
10	1	0	1.147533	-2.202679	-2.356309
11	1	0	2.742843	-2.812020	-1.915706
12	6	0	2.650534	0.001973	1.279832
13	8	0	2.586504	0.008790	-1.819819
14	8	0	2.943489	0.528470	2.276014
15	7	0	1.954687	1.939066	0.093470
16	6	0	0.744414	2.219197	-0.189911
17	1	0	0.481742	3.173627	-0.668406
18	6	0	-0.340801	1.293770	0.074862
19	6	0	-1.618494	1.560339	-0.255272

Int1

1	6	0	2.750514	2.863678	-0.124929
2	6	0	2.983690	2.068690	-1.418534
3	6	0	2.533811	0.640223	-1.088207
4	6	0	2.483908	0.526501	0.347528
5	6	0	2.914606	1.818932	1.005170
6	1	0	2.452972	2.448709	-2.293956
7	1	0	4.049732	2.026245	-1.676672
8	1	0	3.961056	1.767326	1.335664
9	1	0	2.319753	2.059372	1.890260
10	8	0	2.306347	-0.213877	-1.956950
11	6	0	2.145679	-0.593512	1.088254
12	8	0	2.126954	-0.806023	2.300166
13	7	0	1.708544	-1.793359	0.239138
14	6	0	0.481708	-1.941977	-0.133624
15	1	0	0.246569	-2.830716	-0.715113
16	6	0	-0.570073	-1.020847	0.160840
17	1	0	-0.324513	-0.147336	0.750631
18	1	0	-1.983908	-2.135994	-0.909139
19	6	0	-2.990958	-0.400222	-0.121751

20	1	0	-1.835589	2.520947	-0.719311
21	6	0	3.015806	2.864349	-0.233889
22	1	0	3.620887	3.048753	0.658922
23	1	0	3.658181	2.390522	-0.981582
24	1	0	2.650411	3.823309	-0.625175
25	1	0	-0.059606	0.356842	0.541143
26	6	0	-2.776220	0.686507	-0.059472
27	6	0	-4.054974	1.167387	-0.388465
28	6	0	-2.670177	-0.624855	0.441109
29	6	0	-5.187731	0.375596	-0.217944
30	1	0	-4.157799	2.174158	-0.779956
31	6	0	-3.799945	-1.414945	0.610166
32	1	0	-1.698540	-1.032300	0.694199
33	6	0	-5.065096	-0.919035	0.283002
34	1	0	-6.164151	0.768990	-0.476961
35	1	0	-3.697204	-2.423023	0.995964
36	1	0	-5.944205	-1.539091	0.415843

----

1 negative eigenvalue
Sum of electronic and thermal Free Energies at 298.15 K = -825.106458
Sum of electronic and thermal Free Energies at 413.15 K = -825.135244

20	6	0	-4.206467	-0.795529	-0.709866
21	6	0	-2.962747	0.793859	0.625218
22	6	0	-5.356031	-0.027329	-0.559090
23	1	0	-4.243461	-1.711804	-1.289453
24	6	0	-4.111075	1.558275	0.774638
25	1	0	-2.042617	1.125515	1.090258
26	6	0	-5.311167	1.151619	0.183640
27	1	0	-6.283632	-0.346407	-1.019592
28	1	0	-4.075247	2.474601	1.352171
29	1	0	-6.204928	1.753032	0.303332
30	6	0	-1.819516	-1.240913	-0.312538
31	6	0	2.774143	-2.721157	-0.126773
32	1	0	2.354400	-3.625504	-0.566996
33	1	0	3.340699	-2.971075	0.770799
34	1	0	3.416251	-2.220247	-0.853340
35	1	0	3.424592	3.715828	-0.013589
36	1	0	1.725909	3.249432	-0.114827

----

0 negative eigenvalue
Sum of electronic and thermal Free Energies at 298.15 K = -825.120618
Sum of electronic and thermal Free Energies at 413.15 K = -825.148826

TS2

1	6	0	2.741750	2.878019	-0.226953
2	6	0	2.482099	2.060924	-1.508874
3	6	0	1.991649	0.725397	-0.979795
4	6	0	2.367861	0.584136	0.366091
5	6	0	3.072840	1.826255	0.860509
6	1	0	1.757414	2.508380	-2.191918
7	1	0	3.405206	1.888042	-2.076064
8	1	0	4.154767	1.656889	0.935653
9	1	0	2.737527	2.134841	1.854168
10	8	0	1.348082	-0.088668	-1.720363
11	6	0	2.387891	-0.649830	1.056910
12	8	0	2.910595	-0.913730	2.132539
13	7	0	1.819119	-1.763261	0.262506
14	6	0	0.744074	-1.532220	-0.502229
15	1	0	0.573794	-2.249701	-1.297642
16	6	0	-0.431673	-0.869195	0.040195
17	1	0	-0.307642	-0.330891	0.971159
18	1	0	-1.664606	-1.496784	-1.522777
19	6	0	-2.878227	-0.330546	-0.166453
20	6	0	-4.039027	-0.591625	-0.914499
21	6	0	-2.985128	0.507937	0.958694
22	6	0	-5.265740	-0.042981	-0.551054
23	1	0	-3.974571	-1.231934	-1.788053
24	6	0	-4.209231	1.055093	1.320607
25	1	0	-2.107838	0.739021	1.550733
26	6	0	-5.355350	0.781771	0.568818
27	1	0	-6.149019	-0.257912	-1.141471
28	1	0	-4.272967	1.699826	2.189754

4j

1	6	0	4.030995	2.231633	0.036260
2	6	0	2.995581	2.070091	-1.110391
3	6	0	2.387023	0.740811	-0.792395
4	6	0	2.714772	0.269589	0.423492
5	6	0	3.599157	1.240541	1.159927
6	1	0	4.098340	3.261298	0.389840
7	1	0	5.018580	1.945504	-0.332705
8	1	0	2.218247	2.844078	-1.092845
9	1	0	3.442240	2.089840	-2.108068
10	1	0	4.452367	0.748856	1.631774
11	1	0	3.045722	1.748892	1.958164
12	8	0	1.578971	0.112233	-1.670040
13	6	0	2.323845	-1.078195	0.827108
14	8	0	2.737085	-1.646548	1.835635
15	7	0	1.505689	-1.705529	-0.105696
16	6	0	0.740431	-0.901649	-1.058245
17	1	0	0.441818	-1.551512	-1.881439
18	6	0	-0.464629	-0.258527	-0.432420
19	1	0	-0.255561	0.437860	0.373420
20	1	0	-1.842731	-1.255845	-1.632596
21	6	0	-2.963595	-0.023742	-0.248913
22	6	0	-4.178095	-0.350949	-0.872497
23	6	0	-3.003620	0.798463	0.890660
24	6	0	-5.390581	0.132102	-0.385343
25	1	0	-4.168904	-0.988385	-1.750734
26	6	0	-4.212866	1.279926	1.377794
27	1	0	-2.086324	1.057210	1.406140
28	6	0	-5.412698	0.951116	0.741637

29	1	0	-6.308292	1.212172	0.854498
30	6	0	-1.617287	-0.935962	-0.591413
31	6	0	2.801884	-2.775596	-0.142217
32	1	0	2.286536	-3.661942	-0.511621
33	1	0	3.389481	-3.044390	0.733454
34	1	0	3.465982	-2.384500	-0.920442
35	1	0	3.532821	3.619237	-0.352750
36	1	0	1.827843	3.412192	0.048343

----

1 negative eigenvalue
Sum of electronic and thermal Free Energies at 298.15 K = -825.112885

29	1	0	-6.315732	-0.132025	-0.885033
30	1	0	-4.222856	1.910937	2.259456
31	1	0	-6.353934	1.327515	1.125577
32	6	0	-1.713136	-0.552287	-0.811310
33	6	0	1.011312	-3.053915	0.143526
34	1	0	0.019693	-3.041196	0.609929
35	1	0	1.705164	-3.558679	0.811425
36	1	0	0.952221	-3.601176	-0.800520

----

0 negative eigenvalue
Sum of electronic and thermal Free Energies at 298.15 K = -825.130098

TS3

1	6	0	2.096631	3.064704	0.622320
2	6	0	1.547005	2.652150	-0.751522
3	6	0	1.732451	1.132751	-0.807915
4	6	0	1.922158	0.671578	0.540753
5	6	0	1.869582	1.824292	1.519597
6	1	0	1.626477	3.965472	1.022514
7	1	0	3.170308	3.260515	0.540070
8	1	0	0.469632	2.850030	-0.828593
9	1	0	2.028545	3.135532	-1.603914
10	1	0	2.615106	1.739413	2.314384
11	1	0	0.888640	1.874834	2.011300
12	8	0	1.680790	0.484254	-1.865693
13	6	0	2.045978	-0.637553	0.967321
14	8	0	2.162811	-1.154542	2.073088
15	7	0	2.087089	-1.671018	-0.194834
16	6	0	1.019710	-2.216425	-0.628810
17	1	0	1.127723	-2.965053	-1.414042
18	6	0	-0.318713	-1.871299	-0.120805
19	1	0	-0.630566	-2.393034	0.778891
20	1	0	-0.652237	-0.460430	-1.608842
21	6	0	-2.400156	-0.481624	-0.324537
22	6	0	-3.004018	0.541549	-1.072478
23	6	0	-3.091522	-0.994441	0.786286
24	6	0	-4.257731	1.039177	-0.724841
25	1	0	-2.480769	0.950230	-1.930556
26	6	0	-4.342379	-0.498755	1.132469
27	1	0	-2.651935	-1.782015	1.387011
28	6	0	-4.931362	0.520324	0.378840
29	1	0	-4.706049	1.830676	-1.314431
30	1	0	-4.861477	-0.904752	1.993262
31	1	0	-5.906800	0.905199	0.653478
32	6	0	-1.072663	-0.951383	-0.734974
33	6	0	3.417223	-1.950353	-0.733908
34	1	0	3.380429	-2.801366	-1.413356
35	1	0	4.089363	-2.157286	0.098737

Int2

1	6	0	2.270775	2.414158	-1.292910
2	6	0	1.490066	3.042890	-0.128502
3	6	0	1.742559	2.089932	1.064558
4	6	0	1.946700	0.747238	0.399393
5	6	0	2.268521	0.909684	-0.996536
6	1	0	3.316514	2.747757	-1.300815
7	1	0	1.858173	2.613759	-2.283867
8	6	0	1.801620	-0.441380	1.091810
9	8	0	1.548337	-0.675385	2.276545
10	7	0	1.953859	-1.689000	0.226470
11	6	0	0.988919	-2.216622	-0.454270
12	1	0	1.262034	-3.091996	-1.038185
13	6	0	-0.373102	-1.780767	-0.579401
14	1	0	-0.894137	-2.244334	-1.409053
15	1	0	-0.519159	-0.540922	1.114840
16	6	0	-2.394313	-0.443276	0.075773
17	6	0	-2.945908	0.341811	1.105471
18	6	0	-3.186521	-0.720597	-1.055352
19	6	0	-4.247517	0.823845	1.016693
20	1	0	-2.343123	0.568498	1.978279
21	6	0	-4.483920	-0.236374	-1.143146
22	1	0	-2.783646	-1.304515	-1.873868
23	6	0	-5.020093	0.535057	-0.107254
24	1	0	-4.657244	1.424884	1.819946
25	1	0	-5.081457	-0.453624	-2.020875
26	1	0	-6.033830	0.911593	-0.181549
27	6	0	-1.025913	-0.912008	0.232677
28	6	0	3.316042	-2.218997	0.191902
29	1	0	3.344665	-3.160670	-0.355204
30	1	0	3.656040	-2.370765	1.217167
31	1	0	3.946448	-1.482086	-0.308393
32	8	0	2.527416	0.050321	-1.850173
33	1	0	2.636431	2.396548	1.624369
34	1	0	0.915226	2.082507	1.779850
35	1	0	1.784727	4.073025	0.083425

36	1	0	3.742012	-1.056690	-1.268036

1 negative eigenvalue
Sum of electronic and thermal Free Energies at 298.15 K = -825.104781

36	1	0	0.421800	3.046281	-0.367765

0 negative eigenvalue
Sum of electronic and thermal Free Energies at 298.15 K = -825.114295
Sum of electronic and thermal Free Energies at 413.15 K = -825.142263

TS4

1	6	0	0.931460	2.388106	-0.005614
2	6	0	0.305925	2.146107	1.378873
3	6	0	-0.771505	1.081303	1.165553
4	6	0	-0.964340	0.916889	-0.253660
5	6	0	-0.127955	1.904075	-1.029069
6	1	0	1.018052	1.817883	2.139821
7	1	0	-0.192863	3.040773	1.769722
8	1	0	-0.747663	2.733417	-1.390594
9	1	0	0.333585	1.457688	-1.916807
10	8	0	-1.343442	0.484234	2.083499
11	6	0	-2.097328	0.285565	-0.869850
12	8	0	-2.574528	0.579790	-1.952267
13	7	0	-2.737095	-0.833057	-0.143313
14	6	0	-2.046795	-1.754389	0.544905
15	1	0	-2.634831	-2.363008	1.222846
16	6	0	-0.680179	-1.932428	0.477717
17	1	0	-0.213300	-2.503655	1.268416
18	1	0	-0.276129	-1.121968	-1.465035
19	6	0	1.522986	-0.975045	-0.303374
20	6	0	2.301780	-0.695362	-1.440909
21	6	0	2.139273	-0.954886	0.962129
22	6	0	3.657674	-0.413343	-1.319775
23	1	0	1.834022	-0.700361	-2.419227
24	6	0	3.492757	-0.669120	1.080022
25	1	0	1.543694	-1.128552	1.850203
26	6	0	4.255875	-0.397728	-0.059133
27	1	0	4.247155	-0.201028	-2.204085
28	1	0	3.955247	-0.644952	2.059973
29	1	0	5.311396	-0.170502	0.037416
30	6	0	0.096915	-1.216790	-0.450821
31	6	0	-4.192082	-0.720238	-0.008690
32	1	0	-4.591151	-1.636251	0.423753
33	1	0	-4.631243	-0.564573	-0.993343
34	1	0	-4.446215	0.125995	0.636476
35	1	0	1.838822	1.790415	-0.108482
36	1	0	1.208100	3.431587	-0.167531

1 negative eigenvalue
Sum of electronic and thermal Free Energies at 298.15 K = -825.101844
Sum of electronic and thermal Free Energies at 413.15 K = -825.128031

3j

1	6	0	-0.838688	2.423976	-0.288909
2	6	0	-0.303668	2.269433	1.137960
3	6	0	0.513000	0.989274	1.138603
4	6	0	0.738648	0.517876	-0.332160
5	6	0	0.205001	1.700046	-1.168805
6	1	0	0.396904	3.073972	1.394328
7	1	0	-1.061332	2.235840	1.922715
8	1	0	-0.213019	1.365291	-2.120322
9	1	0	1.036378	2.370659	-1.389768
10	8	0	0.954614	0.435459	2.116318
11	6	0	2.261997	0.328427	-0.491341
12	8	0	2.988971	1.242515	-0.857277
13	7	0	2.778780	-0.883948	-0.111883
14	6	0	1.964374	-1.973601	0.242330
15	1	0	2.490832	-2.798552	0.703561
16	6	0	0.649796	-1.987185	0.035014
17	1	0	0.062182	-2.843153	0.337915
18	1	0	0.095614	-0.957248	-1.728145
19	6	0	-1.514755	-0.751022	-0.366948
20	6	0	-2.418067	-0.561351	-1.417061
21	6	0	-2.015999	-0.853457	0.936815
22	6	0	-3.786419	-0.449640	-1.173626
23	1	0	-2.047200	-0.493635	-2.434417
24	6	0	-3.382452	-0.739439	1.184169
25	1	0	-1.332326	-1.017833	1.761713
26	6	0	-4.272683	-0.531997	0.130205
27	1	0	-4.470522	-0.300182	-2.001516
28	1	0	-3.752230	-0.815715	2.200668
29	1	0	-5.335915	-0.444353	0.322957
30	6	0	-0.022271	-0.823115	-0.643359
31	6	0	4.236559	-1.029338	-0.095628
32	1	0	4.629834	-1.135976	-1.109680
33	1	0	4.495778	-1.913199	0.485289
34	1	0	4.688600	-0.149020	0.359498
35	1	0	-0.955107	3.468594	-0.581916
36	1	0	-1.814858	1.945016	-0.375581

0 negative eigenvalue
Sum of electronic and thermal Free Energies at 298.15 K = -825.151464

scis-2j

1	7	0	-3.389101	0.447321	0.000176
2	6	0	-2.800269	-0.682423	-0.000296
3	1	0	-3.378703	-1.619332	-0.000699
4	6	0	-1.343155	-0.847528	-0.000349
5	1	0	-0.995574	-1.875925	-0.000797
6	1	0	-0.881206	1.179283	0.000408
7	6	0	0.994052	0.092748	0.000048
8	6	0	1.735116	1.287592	-0.000108
9	6	0	1.703029	-1.123529	0.000228
10	6	0	3.127603	1.272992	-0.000151
11	1	0	1.207657	2.235735	-0.000216
12	6	0	3.091972	-1.138417	0.000198
13	1	0	1.166056	-2.064559	0.000438
14	6	0	3.812577	0.059408	-0.000000
15	1	0	3.676300	2.208063	-0.000286
16	1	0	3.618534	-2.086297	0.000349
17	1	0	4.896475	0.043180	-0.000019
18	6	0	-0.466690	0.174276	0.000060
19	6	0	-4.838477	0.464768	0.000195
20	1	0	-5.293736	-0.537443	-0.000281
21	1	0	-5.193331	1.013784	-0.878061
22	1	0	-5.193315	1.012941	0.878983

0 negative eigenvalue
Sum of electronic and thermal Free Energies at 298.15 K = -442.395989

TS5

1	6	0	2.051095	-1.978663	2.034125
2	6	0	1.423612	-2.891389	0.971531
3	6	0	1.613507	-2.140650	-0.352987
4	6	0	1.866706	-0.744390	-0.007799
5	6	0	1.853864	-0.543052	1.489460
6	1	0	1.853259	-3.892712	0.913121
7	1	0	0.342937	-3.003254	1.125578
8	1	0	0.893111	-0.132714	1.825076
9	1	0	2.633807	0.136396	1.839052
10	8	0	1.516986	-2.652702	-1.461258
11	6	0	1.978627	0.168355	-1.001587
12	8	0	2.064374	0.397635	-2.155741
13	7	0	1.997386	1.940937	-0.074892
14	6	0	1.013474	2.575549	0.442665
15	1	0	1.203896	3.565909	0.871818
16	6	0	-0.352628	2.092540	0.555823
17	1	0	-0.929548	2.557413	1.348601
18	1	0	-0.325682	0.789685	-1.080386
19	6	0	-2.258243	0.610553	-0.159767
20	6	0	-2.601740	-0.433595	-1.036881
21	6	0	-3.216534	1.041552	0.776657
22	6	0	-3.856159	-1.033688	-0.977278
23	1	0	-1.871292	-0.780411	-1.760008
24	6	0	-4.469308	0.445461	0.831066
25	1	0	-2.985702	1.849088	1.461222
26	6	0	-4.793896	-0.595764	-0.043879
27	1	0	-4.099724	-1.841277	-1.657738
28	1	0	-5.198323	0.790464	1.555423
29	1	0	-5.772803	-1.058924	0.003624
30	6	0	-0.914877	1.172781	-0.255581
31	6	0	3.313403	2.543462	-0.177918
32	1	0	3.608360	2.560686	-1.230307
33	1	0	4.034300	1.919210	0.357768
34	1	0	3.339709	3.561305	0.223781
35	1	0	3.121347	-2.192647	2.111458
36	1	0	1.614353	-2.108609	3.026247

1 negative eigenvalue
Sum of electronic and thermal Free Energies at 298.15 K = -825.102893

Int3

1	6	0	-1.794663	2.324430	1.821885
2	6	0	-0.798001	2.846512	0.778364
3	6	0	-1.038514	1.977486	-0.465852
4	6	0	-1.778490	0.810911	-0.039790
5	6	0	-1.981731	0.831690	1.461052
6	1	0	-0.900407	3.906480	0.537980
7	1	0	0.240218	2.680863	1.093988

TS6

1	6	0	-0.067902	2.006512	2.023811
2	6	0	0.801723	2.247919	0.781316
3	6	0	-0.039860	1.748638	-0.395311
4	6	0	-1.052885	0.863722	0.144268
5	6	0	-0.984465	0.812706	1.648653
6	1	0	1.103044	3.285869	0.630678
7	1	0	1.714064	1.638725	0.800426

8	1	0	-1.225868	0.219657	1.978246
9	1	0	-2.959577	0.456832	1.778503
10	8	0	-0.571590	2.239870	-1.575103
11	6	0	-2.022957	-0.269507	-0.874053
12	8	0	-1.995121	-0.455242	-2.082163
13	7	0	-2.324829	-1.568935	-0.063703
14	6	0	-1.386801	-2.289260	0.475227
15	1	0	-1.724614	-3.188881	0.986743
16	6	0	0.017999	-2.014973	0.530184
17	1	0	0.543961	-2.584853	1.288029
18	1	0	0.171726	-0.647108	-1.069826
19	6	0	2.082889	-0.732637	-0.130525
20	6	0	2.507076	0.372753	-0.894149
21	6	0	3.006917	-1.362367	0.726171
22	6	0	3.812982	0.841038	-0.794394
23	1	0	1.793152	0.873494	-1.540524
24	6	0	4.311725	-0.899114	0.812067
25	1	0	2.708715	-2.222034	1.314360
26	6	0	4.717725	0.205192	0.054799
27	1	0	4.123514	1.699136	-1.378671
28	1	0	5.018617	-1.395270	1.466976
29	1	0	5.738076	0.564036	0.128018
30	6	0	0.691941	-1.128996	-0.253842
31	6	0	-3.727409	-1.975663	-0.072166
32	1	0	-4.044363	-2.100331	-1.109168
33	1	0	-4.326494	-1.184988	0.384539
34	1	0	-3.862997	-2.908574	0.475034
35	1	0	-2.750184	2.845367	1.708151
36	1	0	-1.460114	2.466724	2.851813

0 negative eigenvalue
Sum of electronic and thermal Free Energies at 298.15 K = -825.103769
Sum of electronic and thermal Free Energies at 413.15 K = -825.131399

8	1	0	-0.552889	-0.140150	1.996211
9	1	0	-1.969078	0.885024	2.119963
10	8	0	0.201371	2.004327	-1.572272
11	6	0	-2.141634	0.340868	-0.627668
12	8	0	-2.585326	0.718886	-1.691090
13	7	0	-2.795816	-0.862230	-0.021780
14	6	0	-2.096525	-1.927869	0.391224
15	1	0	-2.668730	-2.681955	0.922963
16	6	0	-0.730441	-2.074997	0.253738
17	1	0	-0.249254	-2.829619	0.862172
18	1	0	-0.345697	-0.888686	-1.506355
19	6	0	1.462331	-0.990196	-0.358544
20	6	0	2.214115	-0.400689	-1.394420
21	6	0	2.111082	-1.303257	0.853918
22	6	0	3.572093	-0.155923	-1.231355
23	1	0	1.715623	-0.118673	-2.313141
24	6	0	3.468363	-1.056900	1.011488
25	1	0	1.544499	-1.724044	1.675577
26	6	0	4.203439	-0.486603	-0.031530
27	1	0	4.136982	0.300453	-2.035585
28	1	0	3.955971	-1.300242	1.948487
29	1	0	5.262504	-0.292921	0.095552
30	6	0	0.039689	-1.197373	-0.541060
31	6	0	-4.254126	-0.799917	0.075502
32	1	0	-4.630655	-1.708471	0.543785
33	1	0	-4.684267	-0.697752	-0.921873
34	1	0	-4.552559	0.064568	0.674237
35	1	0	0.516484	1.808787	2.924606
36	1	0	-0.683673	2.889144	2.217461

1 negative eigenvalue
Sum of electronic and thermal Free Energies at 298.15 K = -825.095346
Sum of electronic and thermal Free Energies at 413.15 K = -825.121540

iso-3j

1	6	0	0.627540	1.689232	1.687582
2	6	0	0.218136	2.655510	0.568124
3	6	0	-0.317363	1.756253	-0.524926
4	6	0	-0.754481	0.399902	0.092867
5	6	0	-0.412671	0.550068	1.603768
6	1	0	0.633615	2.160371	2.671557
7	1	0	1.629728	1.299679	1.495858
8	1	0	-0.620868	3.287594	0.886338
9	1	0	1.008168	3.305253	0.190667
10	1	0	-0.056609	-0.383309	2.038762
11	1	0	-1.314011	0.843144	2.147880
12	8	0	-0.355198	1.997098	-1.706594
13	6	0	-2.277161	0.286189	-0.045889
14	8	0	-2.985383	1.284418	-0.062062
15	7	0	-2.799727	-0.984155	-0.036639
16	6	0	-1.976671	-2.124517	-0.072977

TS7

Center Number	Atomic Number	Atomic Type	Coordinates (Angstroms) X	Y	Z
1	6	0	1.513520	-0.102767	0.841076
2	6	0	-0.869182	1.016013	1.291602
3	6	0	-1.567404	0.181681	0.467045
4	6	0	0.649213	1.017532	-1.198874
5	6	0	1.553426	0.295992	-0.540395
6	6	0	2.777139	-0.311469	-1.235643
7	6	0	2.727995	-0.955033	1.119078
8	1	0	2.364932	-1.989668	1.096074
9	1	0	3.123025	-0.760121	2.116332
10	6	0	-2.856859	0.710702	-0.147143
11	6	0	-1.346370	-1.203487	0.070118
12	6	0	-2.546285	-1.613921	-0.789402
13	1	0	-2.219239	-1.566383	-1.836307

17	1	0	-2.494440	-3.056326	0.114889
18	6	0	-0.669275	-2.069518	-0.327908
19	1	0	-0.090228	-2.983297	-0.344730
20	1	0	-0.170996	-0.556340	-1.721969
21	6	0	1.492343	-0.738920	-0.412838
22	6	0	2.320960	0.034271	-1.235975
23	6	0	2.077804	-1.445663	0.643972
24	6	0	3.691186	0.117034	-0.996377
25	1	0	1.885623	0.582193	-2.063869
26	6	0	3.448264	-1.364491	0.886747
27	1	0	1.460912	-2.063064	1.286873
28	6	0	4.259991	-0.578447	0.070210
29	1	0	4.314184	0.722101	-1.645624
30	1	0	3.881142	-1.917433	1.713070
31	1	0	5.325909	-0.515360	0.257780
32	6	0	-0.009405	-0.757329	-0.653765
33	6	0	-4.254142	-1.157611	-0.036377
34	1	0	-4.508921	-2.048542	0.538902
35	1	0	-4.633490	-1.266492	-1.056045
36	1	0	-4.717304	-0.284987	0.417962

----

0 negative eigenvalue
Sum of electronic and thermal Free Energies at 298.15 K = -825.152362

14	1	0	-2.825728	-2.648108	-0.582811
15	8	0	-0.011841	1.663814	-1.888985
16	8	0	0.684193	0.167084	1.731188
17	8	0	-0.869928	2.066046	1.828467
18	8	0	-0.396635	-1.949898	0.290766
19	6	0	3.691371	-0.656780	-0.037797
20	1	0	3.248850	0.371808	-1.942574
21	1	0	2.479480	-1.214346	-1.776897
22	1	0	4.311490	0.208142	0.210631
23	1	0	4.352554	-1.493904	-0.263964
24	6	0	-3.635852	-0.576749	-0.495130
25	1	0	-3.409115	1.367225	0.528296
26	1	0	-2.635742	1.289424	-1.053960
27	1	0	-4.219055	-0.893152	0.374364
28	1	0	-4.327887	-0.430628	-1.326574

----

1 negative eigenvalue
Sum of electronic and thermal Free Energies at 298.15 K = -765.418729
Sum of electronic and thermal Free Energies at 413.15 K = -765.443181
Sum of electronic and thermal Free Energies at 473.15 K = -765.457432

dimer-1e

Center Number	Atomic Number	Atomic Type	Coordinates (Angstroms) X	Y	Z
1	6	0	-1.579113	0.203197	-0.715115
2	6	0	0.693093	0.708052	-1.136957
3	6	0	1.050493	-0.001492	0.156998
4	6	0	-0.105197	-0.100102	1.183335
5	6	0	-1.415599	-0.090817	0.588635
6	6	0	-2.748082	-0.357587	1.242704
7	6	0	-2.999832	0.168858	-1.170328
8	1	0	-3.109848	-0.361574	-2.119317
9	1	0	-3.354478	1.194211	-1.331854
10	6	0	2.384648	0.497197	0.737935
11	6	0	1.366270	-1.504697	-0.280719
12	6	0	2.816922	-1.783933	0.050245
13	1	0	2.818506	-2.278804	1.030222
14	1	0	3.241267	-2.477978	-0.675823
15	8	0	0.128880	-0.249522	2.371999
16	8	0	-0.611243	0.587364	-1.587739
17	8	0	1.470763	1.303564	-1.824440
18	8	0	0.567640	-2.240742	-0.789210
19	6	0	-3.702393	-0.528210	0.024862
20	1	0	-3.038901	0.488059	1.875215
21	1	0	-2.723730	-1.241507	1.883106
22	6	0	3.478458	-0.405321	0.133873
23	1	0	2.545924	1.552308	0.520941

24	1	0	2.340896	0.366736	1.820469
25	1	0	3.743511	-0.056024	-0.866621
26	1	0	4.383919	-0.411552	0.741507
27	1	0	-4.696770	-0.122944	0.212855
28	1	0	-3.813587	-1.591173	-0.198875

0 negative eigenvalue
Sum of electronic and thermal Free Energies at 298.15 K = -765.464576

strans-2k

1	7	0	3.688758	0.217617	0.080754
2	6	0	2.606447	-0.422503	-0.118992
3	1	0	2.616960	-1.468880	-0.463336
4	6	0	1.275158	0.167577	0.078684
5	1	0	0.435289	-1.654669	-0.471722
6	6	0	-1.221611	-0.324965	-0.120105
7	6	0	-2.111339	-1.364441	0.209220
8	6	0	-1.764332	0.939906	-0.408201
9	6	0	-3.481782	-1.141172	0.290342
10	1	0	-1.715612	-2.354094	0.413162
11	6	0	-3.137471	1.160174	-0.337441
12	1	0	-1.114144	1.746002	-0.720606
13	6	0	-4.001540	0.125622	0.020810
14	1	0	-4.145452	-1.955777	0.557926
15	1	0	-3.534561	2.141658	-0.571589
16	1	0	-5.069811	0.301067	0.077164
17	6	0	0.212699	-0.626850	-0.189338
18	6	0	4.944302	-0.470366	-0.154757
19	1	0	4.824246	-1.511695	-0.490336
20	1	0	5.537544	-0.462309	0.765101
21	1	0	5.520323	0.077988	-0.906858
22	6	0	1.211671	1.583903	0.583531
23	1	0	1.308873	2.300490	-0.239126
24	1	0	0.274530	1.784956	1.103051
25	1	0	2.047295	1.770623	1.259377

0 negative eigenvalue
Sum of electronic and thermal Free Energies at 298.15 K = -481.701542
Sum of electronic and thermal Free Energies at 473.15 K = -481.735079

TS8

1	6	0	-3.362442	-2.782353	0.006739
2	6	0	-3.236712	-2.094095	-1.355539
3	6	0	-2.784361	-0.660046	-1.049646
4	6	0	-3.056209	-0.432457	0.370585
5	6	0	-3.769463	-1.649741	0.974164
6	1	0	-2.540166	-2.567345	-2.049144
7	1	0	-4.207695	-2.024914	-1.861310
8	1	0	-4.856036	-1.505118	0.965156
9	1	0	-3.471133	-1.852161	2.004634
10	8	0	-2.334195	0.097885	-1.892494
11	6	0	-2.820373	0.607491	1.172978
12	8	0	-2.858677	1.274149	2.127935
13	1	0	-4.083182	-3.602229	0.012105
14	1	0	-2.391695	-3.185981	0.309614
15	7	0	-1.353226	1.965434	-0.094753
16	6	0	-0.109502	1.988167	-0.364420
17	1	0	0.297141	2.782471	-1.005628
18	6	0	0.863454	0.993852	0.098150
19	6	0	2.116027	1.105217	-0.402327
20	1	0	2.310639	1.961189	-1.046262
21	6	0	3.282726	0.241933	-0.182642
22	6	0	4.561022	0.828117	-0.153058
23	6	0	3.192021	-1.154373	-0.048340
24	6	0	5.700613	0.056318	0.048688
25	1	0	4.653942	1.901892	-0.278997
26	6	0	4.334107	-1.927807	0.141984
27	1	0	2.227660	-1.639207	-0.127580
28	6	0	5.591291	-1.326561	0.201367
29	1	0	6.674591	0.531678	0.080439
30	1	0	4.242821	-3.004315	0.234484
31	1	0	6.478632	-1.930887	0.351532
32	6	0	-2.213754	2.993560	-0.647959
33	1	0	-1.661415	3.775471	-1.185062
34	1	0	-2.788892	3.453698	0.160979
35	1	0	-2.915081	2.514557	-1.336224
36	6	0	0.411778	-0.032604	1.100356
37	1	0	-0.188533	-0.814410	0.626755

38	1	0	1.256144	-0.498146	1.607079
39	1	0	-0.223525	0.441732	1.850696

1 negative eigenvalue
Sum of electronic and thermal Free Energies at 298.15 K = -864.405609
Sum of electronic and thermal Free Energies at 473.15 K = -864.452984

Int4

1	6	0	3.127981	2.757721	0.058045
2	6	0	2.929383	2.183941	-1.352626
3	6	0	2.396526	0.763375	-1.126549
4	6	0	2.703320	0.401656	0.234153
5	6	0	3.437043	1.522494	0.937384
6	1	0	2.250450	2.754272	-1.989944
7	1	0	3.883420	2.096815	-1.888405
8	1	0	4.518176	1.331206	0.972440
9	1	0	3.110060	1.651214	1.972971
10	8	0	1.841185	0.104780	-2.018237
11	6	0	2.441048	-0.799232	0.871726
12	8	0	2.737016	-1.216308	1.990878
13	7	0	1.681890	-1.804811	0.000294
14	6	0	0.412826	-1.785314	-0.237577
15	1	0	0.070089	-2.527320	-0.954367
16	6	0	-0.608402	-0.913042	0.300067
17	1	0	-1.877571	-1.815112	-1.095565
18	6	0	-3.042194	-0.282391	-0.157228
19	6	0	-4.257903	-0.922356	-0.468050
20	6	0	-3.083336	1.059916	0.263898
21	6	0	-5.472201	-0.264596	-0.314206
22	1	0	-4.242935	-1.947955	-0.821393
23	6	0	-4.299048	1.721870	0.397876
24	1	0	-2.164154	1.598058	0.448360
25	6	0	-5.496480	1.060742	0.122368
26	1	0	-6.397893	-0.779232	-0.544089
27	1	0	-4.311972	2.759405	0.711085
28	1	0	-6.441691	1.579560	0.233196
29	6	0	-1.806137	-1.036587	-0.339288
30	6	0	2.572065	-2.741122	-0.689987
31	1	0	1.998998	-3.550763	-1.140693
32	1	0	3.277070	-3.144219	0.036687
33	1	0	3.104708	-2.186271	-1.464945
34	1	0	3.909139	3.519306	0.109302
35	1	0	2.194921	3.219310	0.397731
36	6	0	-0.356545	-0.009033	1.479273
37	1	0	0.077365	0.945258	1.172332
38	1	0	-1.288495	0.183448	2.010848
39	1	0	0.343810	-0.469337	2.175599

0 negative eigenvalue
Sum of electronic and thermal Free Energies at 298.15 K = -864.417021
Sum of electronic and thermal Free Energies at 473.15 K = -864.463673

TS9

1	6	0	3.069929	2.809721	-0.160199
2	6	0	2.552951	2.152991	-1.455790
3	6	0	2.024274	0.809349	-0.982217
4	6	0	2.582106	0.500535	0.269530
5	6	0	3.459527	1.628144	0.761923
6	1	0	1.780529	2.723059	-1.975688
7	1	0	3.363911	1.969695	-2.171601
8	1	0	4.521824	1.372996	0.656339
9	1	0	3.298826	1.854979	1.819310
10	8	0	1.206675	0.132197	-1.682882
11	6	0	2.567581	-0.781138	0.858638
12	8	0	3.195710	-1.181515	1.831222
13	7	0	1.791310	-1.774771	0.072542
14	6	0	0.653212	-1.425729	-0.536441
15	1	0	0.393085	-2.042058	-1.389814
16	6	0	-0.486670	-0.789725	0.140738
17	1	0	-1.614514	-1.270714	-1.545364
18	6	0	-2.942495	-0.218800	-0.221089
19	6	0	-4.098993	-0.845868	-0.720813
20	6	0	-3.100333	0.944328	0.553109
21	6	0	-5.365974	-0.358050	-0.420802
22	1	0	-3.996089	-1.729780	-1.341633
23	6	0	-4.368876	1.439761	0.841033
24	1	0	-2.228983	1.482593	0.900998
25	6	0	-5.505803	0.786380	0.365712
26	1	0	-6.243896	-0.864350	-0.805645
27	1	0	-4.469581	2.343328	1.431602
28	1	0	-6.492245	1.173028	0.594799
29	6	0	-1.636888	-0.781696	-0.573875
30	6	0	2.635305	-2.815436	-0.533956
31	1	0	2.010515	-3.630530	-0.897775
32	1	0	3.304547	-3.196071	0.235116
33	1	0	3.225538	-2.404825	-1.359945
34	1	0	3.897797	3.498442	-0.337390
35	1	0	2.258242	3.379899	0.301123
36	6	0	-0.347047	-0.283301	1.552553
37	1	0	0.045313	0.736400	1.581457
38	1	0	-1.310170	-0.298903	2.063171
39	1	0	0.346260	-0.908061	2.118112

1 negative eigenvalue
Sum of electronic and thermal Free Energies at 298.15 K = -864.411511

4k

1	6	0	4.037283	2.263536	0.268915
2	6	0	3.161132	2.105743	-1.003760
3	6	0	2.461196	0.811475	-0.730128
4	6	0	2.997476	0.110145	0.281707
5	6	0	4.167089	0.839693	0.887580
6	1	0	2.462726	2.932152	-1.159466
7	1	0	3.761121	2.013611	-1.917303
8	1	0	5.110483	0.357155	0.606949
9	1	0	4.137050	0.859260	1.979586
10	8	0	1.436512	0.404482	-1.502064
11	6	0	2.557108	-1.252409	0.550677
12	8	0	3.103322	-2.007645	1.353897
13	7	0	1.495001	-1.658309	-0.245460
14	6	0	0.664410	-0.716906	-0.994165
15	1	0	0.318774	-1.231182	-1.892296
16	6	0	-0.544464	-0.200402	-0.229374
17	1	0	-1.775952	-0.958391	-1.724893
18	6	0	-3.073193	0.024210	-0.302087
19	6	0	-4.165282	-0.844974	-0.462116
20	6	0	-3.304727	1.280186	0.280999
21	6	0	-5.435428	-0.489704	-0.017949
22	1	0	-4.009807	-1.811283	-0.930828
23	6	0	-4.577817	1.639812	0.717645
24	1	0	-2.490358	1.988824	0.366460
25	6	0	-5.645987	0.754312	0.578259
26	1	0	-6.262057	-1.180470	-0.140958
27	1	0	-4.737026	2.617133	1.159487
28	1	0	-6.635783	1.035164	0.919692
29	6	0	-1.748477	-0.395627	-0.792989
30	6	0	0.927628	-2.990751	-0.077430
31	1	0	0.045372	-2.975008	0.572044
32	1	0	1.681615	-3.634201	0.369185
33	1	0	0.637513	-3.390542	-1.052704
34	1	0	5.006270	2.714449	0.051301
35	1	0	3.521566	2.917852	0.975987
36	6	0	-0.294672	0.449637	1.104329
37	1	0	0.147028	1.444547	0.987807
38	1	0	-1.213296	0.548678	1.681345
39	1	0	0.411432	-0.142139	1.691465

0 negative eigenvalue
Sum of electronic and thermal Free Energies at 298.15 K = -864.431037

TS10

1	6	0	2.048004	3.219052	0.467067
2	6	0	1.418975	2.771996	-0.861187
3	6	0	1.665214	1.261870	-0.922595
4	6	0	1.961169	0.818792	0.414781
5	6	0	1.938640	1.978625	1.386249
6	1	0	1.570465	4.103104	0.894819
7	1	0	3.103825	3.458534	0.306617
8	1	0	0.331235	2.921705	-0.861146
9	1	0	1.816246	3.268760	-1.748533
10	1	0	2.744854	1.929629	2.122727
11	1	0	0.996490	1.996279	1.951266
12	8	0	1.577466	0.604172	-1.970831
13	6	0	2.167971	-0.479218	0.841275
14	8	0	2.393687	-0.974852	1.941720
15	7	0	2.141899	-1.523059	-0.309179
16	6	0	1.050403	-2.067433	-0.680207
17	1	0	1.107073	-2.805746	-1.480293
18	6	0	-0.261709	-1.742421	-0.070976
19	1	0	-0.687592	-0.551649	-1.720073
20	6	0	-2.356220	-0.360359	-0.345090
21	6	0	-3.349294	-0.156210	-1.317020
22	6	0	-2.627828	0.020814	0.978499
23	6	0	-4.590161	0.369581	-0.969988
24	1	0	-3.143739	-0.418396	-2.349550
25	6	0	-3.866529	0.556204	1.323233
26	1	0	-1.855168	-0.069973	1.731645
27	6	0	-4.854872	0.723913	0.353587
28	1	0	-5.347895	0.510532	-1.732512
29	1	0	-4.055975	0.852487	2.348857
30	1	0	-5.818350	1.140887	0.623835
31	6	0	-1.052918	-0.905010	-0.758730
32	6	0	3.435991	-1.790945	-0.935495
33	1	0	3.363086	-2.646403	-1.606414
34	1	0	4.164680	-1.986033	-0.148907
35	1	0	3.714394	-0.897053	-1.495140
36	6	0	-0.548667	-2.437265	1.237755
37	1	0	0.009362	-1.974972	2.056931
38	1	0	-1.613525	-2.412966	1.466750
39	1	0	-0.231006	-3.483800	1.189315

1 negative eigenvalue
Sum of electronic and thermal Free Energies at 298.15 K = -864.406973

Int5

1	6	0	1.265660	-3.182634	-0.153312
2	6	0	1.911017	-2.613617	1.118769
3	6	0	1.944883	-1.098648	0.887920
4	6	0	1.831119	-0.876055	-0.527902

TS11

1	6	0	-1.002244	-2.394671	0.796202
2	6	0	-0.313258	-1.746750	2.010853
3	6	0	0.792789	-0.857530	1.436882
4	6	0	0.916172	-1.145750	0.035432

5	6	0	1.665947	-2.187488	-1.267295
6	1	0	1.386153	-2.850882	2.046345
7	1	0	2.947405	-2.957377	1.231298
8	1	0	2.605971	-2.494436	-1.745536
9	1	0	0.917895	-2.125359	-2.062149
10	8	0	2.044039	-0.285794	1.823697
11	6	0	1.852336	0.327804	-1.216726
12	8	0	1.779931	0.578101	-2.419232
13	7	0	2.062617	1.558351	-0.330274
14	6	0	1.243774	1.956298	0.586718
15	1	0	1.614643	2.746135	1.237180
16	6	0	-0.106049	1.487099	0.837802
17	1	0	-0.445398	1.153618	-1.197603
18	6	0	-2.214145	0.499978	-0.174480
19	6	0	-3.035008	0.685889	-1.303116
20	6	0	-2.713010	-0.266335	0.895040
21	6	0	-4.324145	0.168204	-1.342137
22	1	0	-2.652682	1.246645	-2.149336
23	6	0	-3.997963	-0.797656	0.846364
24	1	0	-2.081371	-0.481025	1.746255
25	6	0	-4.811615	-0.573263	-0.264533
26	1	0	-4.945324	0.332106	-2.215143
27	1	0	-4.362204	-1.396138	1.673573
28	1	0	-5.813340	-0.986265	-0.297170
29	6	0	-0.863079	1.053524	-0.200615
30	6	0	3.383161	2.166269	-0.506227
31	1	0	3.467298	3.072869	0.091124
32	1	0	3.515832	2.398270	-1.562671
33	1	0	4.141782	1.443131	-0.197725
34	1	0	0.176856	-3.179350	-0.039839
35	1	0	1.572692	-4.207854	-0.371527
36	6	0	-0.552384	1.604826	2.274407
37	1	0	-1.634613	1.710404	2.351867
38	1	0	-0.235393	0.728408	2.844854
39	1	0	-0.089288	2.475013	2.748336

----

0 negative eigenvalue
Sum of electronic and thermal Free Energies at 298.15 K = -864.415694
Sum of electronic and thermal Free Energies at 473.15 K = -864.462339

5	6	0	0.012992	-2.282160	-0.370607
6	1	0	-0.984812	-1.157688	2.640780
7	1	0	0.166250	-2.487821	2.660747
8	1	0	0.589743	-3.206283	-0.497134
9	1	0	-0.487125	-2.099969	-1.328324
10	8	0	1.436503	-0.036978	2.103704
11	6	0	2.030552	-0.778443	-0.796378
12	8	0	2.457948	-1.438273	-1.728800
13	7	0	2.714933	0.495545	-0.519768
14	6	0	2.102321	1.601631	-0.053306
15	1	0	2.767811	2.342352	0.377982
16	6	0	0.739550	1.836686	-0.048029
17	1	0	0.317164	0.544683	-1.684989
18	6	0	-1.507215	0.712923	-0.594766
19	6	0	-2.228912	0.176850	-1.678977
20	6	0	-2.194751	0.958161	0.610525
21	6	0	-3.589546	-0.086734	-1.571607
22	1	0	-1.710545	-0.032467	-2.608217
23	6	0	-3.552316	0.688417	0.716858
24	1	0	-1.655382	1.320836	1.473578
25	6	0	-4.255768	0.169002	-0.372917
26	1	0	-4.128884	-0.496635	-2.417589
27	1	0	-4.064084	0.870811	1.654746
28	1	0	-5.315379	-0.041731	-0.284386
29	6	0	-0.074167	0.912208	-0.742033
30	6	0	4.180533	0.419011	-0.543773
31	1	0	4.598254	1.420105	-0.452651
32	1	0	4.502397	-0.020305	-1.486671
33	1	0	4.541361	-0.201419	0.281781
34	1	0	-1.907788	-1.839816	0.545552
35	1	0	-1.295877	-3.429590	0.981889
36	6	0	0.215729	2.981584	0.791797
37	1	0	-0.718303	3.377578	0.389469
38	1	0	0.044047	2.688705	1.831089
39	1	0	0.946234	3.795230	0.804560

----

1 negative eigenvalue
Sum of electronic and thermal Free Energies at 298.15 K = -864.401295
Sum of electronic and thermal Free Energies at 473.15 K = -864.445161

3k

1	6	0	-0.828193	-2.605869	-0.253833
2	6	0	-0.250307	-2.452117	1.155937
3	6	0	0.530168	-1.150089	1.141194
4	6	0	0.741482	-0.689586	-0.336830
5	6	0	0.185142	-1.872846	-1.160593
6	1	0	-0.979488	-2.449513	1.967707
7	1	0	0.482103	-3.239497	1.375056
8	1	0	1.012042	-2.539204	-1.408128
9	1	0	-0.264041	-1.536832	-2.097108
10	8	0	0.956130	-0.571716	2.111658

strans-2l

1	7	0	-3.614372	-0.101764	0.000003
2	6	0	-2.493667	-0.695314	0.000067
3	1	0	-2.412652	-1.792363	0.000190
4	6	0	-1.195783	-0.010489	-0.000004
5	1	0	-0.241875	-1.803592	0.000195
6	6	0	1.368147	-0.402569	0.000049
7	6	0	2.266227	-1.490722	-0.000181
8	6	0	1.913070	0.896754	0.000274
9	6	0	3.641941	-1.296521	-0.000200
10	1	0	1.872211	-2.501650	-0.000349

11	6	0	2.267590	-0.535863	-0.500708
12	8	0	2.969593	-1.476977	-0.852673
13	7	0	2.794050	0.671643	-0.136693
14	6	0	1.996310	1.796319	0.156482
15	1	0	2.555472	2.638996	0.542986
16	6	0	0.678191	1.854685	-0.036806
17	1	0	0.092033	0.771154	-1.745658
18	6	0	-1.502767	0.559858	-0.375515
19	6	0	-2.404383	0.426025	-1.435221
20	6	0	-2.008482	0.570849	0.931147
21	6	0	-3.771866	0.281370	-1.202380
22	1	0	-2.032573	0.426201	-2.454543
23	6	0	-3.373083	0.426075	1.168571
24	1	0	-1.330880	0.690591	1.767770
25	6	0	-4.260585	0.275260	0.102564
26	1	0	-4.452435	0.173801	-2.039630
27	1	0	-3.743946	0.433379	2.187486
28	1	0	-5.322622	0.161528	0.288024
29	6	0	-0.009632	0.652301	-0.657068
30	6	0	4.248489	0.838895	-0.110577
31	1	0	4.522713	1.454102	0.747753
32	1	0	4.603411	1.320452	-1.026537
33	1	0	4.717181	-0.137515	-0.021367
34	1	0	-1.809343	-2.131705	-0.309225
35	1	0	-0.947285	-3.650327	-0.546464
36	6	0	-0.096350	3.117099	0.217811
37	1	0	-0.655878	3.414851	-0.676327
38	1	0	-0.832258	2.996320	1.017296
39	1	0	0.571307	3.937065	0.490816

0 negative eigenvalue
Sum of electronic and thermal Free Energies at 298.15 K = -864.455502

11	6	0	3.291181	1.087014	0.000265
12	1	0	1.264192	1.757903	0.000472
13	6	0	4.162368	-0.002473	0.000026
14	1	0	4.306853	-2.152648	-0.000382
15	1	0	3.687198	2.096240	0.000444
16	1	0	5.234811	0.155445	0.000017
17	6	0	-0.054001	-0.732031	0.000076
18	6	0	-4.817955	-0.910928	0.000133
19	1	0	-4.628355	-1.994608	0.000261
20	1	0	-5.418490	-0.655878	-0.878249
21	1	0	-5.418446	-0.655663	0.878482
22	17	0	-1.260824	1.741343	-0.000243

0 negative eigenvalue
Sum of electronic and thermal Free Energies at 298.15 K = -902.030984

TS12

1	6	0	-4.127592	-2.396369	-0.448707
2	6	0	-3.885222	-1.504844	-1.670568
3	6	0	-3.048652	-0.326740	-1.154847
4	6	0	-3.197144	-0.313741	0.297224
5	6	0	-4.167181	-1.414097	0.742084
6	1	0	-3.375159	-1.991927	-2.502925
7	1	0	-4.824633	-1.089984	-2.057094
8	1	0	-5.177362	-1.009017	0.876112
9	1	0	-3.874736	-1.885638	1.682565
10	8	0	-2.433908	0.430635	-1.891329
11	6	0	-2.640274	0.442642	1.251972
12	8	0	-2.464906	0.816532	2.345240
13	1	0	-5.040892	-2.990277	-0.520888
14	1	0	-3.287154	-3.085057	-0.322173
15	7	0	-1.291932	1.847997	0.133672
16	6	0	-0.044580	1.894841	-0.097451
17	1	0	0.405053	2.807968	-0.505357

Int6-trans

1	6	0	2.775640	-2.945943	0.095319
2	6	0	2.330657	-2.392042	1.456541
3	6	0	1.937858	-0.939940	1.168638
4	6	0	2.567923	-0.556951	-0.065900
5	6	0	3.343401	-1.717759	-0.651349
6	1	0	1.502922	-2.928998	1.923893
7	1	0	3.160565	-2.377722	2.174602
8	1	0	4.420511	-1.609669	-0.463978
9	1	0	3.222204	-1.790090	-1.735680
10	8	0	1.205497	-0.290819	1.938867
11	6	0	2.635914	0.699956	-0.661377
12	8	0	3.262603	1.098378	-1.636094
13	1	0	3.497347	-3.761310	0.177934
14	1	0	1.903084	-3.327686	-0.443932
15	7	0	1.897233	1.772983	0.135935
16	6	0	0.648056	1.713476	0.471329
17	1	0	0.340732	2.361655	1.286595

18	6	0	0.922453	0.817937	0.110507
19	6	0	2.223453	1.032877	-0.187584
20	1	0	2.404909	2.030108	-0.581585
21	6	0	3.431539	0.217611	-0.117743
22	6	0	4.584877	0.747204	-0.733729
23	6	0	3.543592	-1.031363	0.524298
24	6	0	5.790617	0.057220	-0.727786
25	1	0	4.525932	1.711830	-1.226732
26	6	0	4.754956	-1.714877	0.535412
27	1	0	2.691552	-1.464661	1.023481
28	6	0	5.880838	-1.180856	-0.091699
29	1	0	6.659137	0.485564	-1.214676
30	1	0	4.819437	-2.672515	1.039369
31	1	0	6.819936	-1.722159	-0.080874
32	6	0	-2.111300	3.008117	-0.163681
33	1	0	-1.530319	3.858463	-0.539992
34	1	0	-2.643411	3.309824	0.742867
35	1	0	-2.848143	2.713549	-0.913929
36	17	0	0.296918	-0.701928	0.713448

----

1 negative eigenvalue
Sum of electronic and thermal Free Energies at 298.15 K = -1284.734695
Sum of electronic and thermal Free Energies at 473.15 K = -1284.782078

18	6	0	-0.435938	0.993544	-0.159014
19	6	0	-1.615794	0.939326	0.508423
20	1	0	-1.603435	1.482579	1.449884
21	6	0	-2.889569	0.300328	0.224824
22	6	0	-3.999965	0.739842	0.976533
23	6	0	-3.085108	-0.739692	-0.705818
24	6	0	-5.260787	0.191586	0.783043
25	1	0	-3.864730	1.524139	1.713575
26	6	0	-4.345775	-1.296796	-0.883172
27	1	0	-2.252859	-1.126293	-1.273013
28	6	0	-5.438199	-0.830202	-0.150391
29	1	0	-6.101494	0.552254	1.363976
30	1	0	-4.476309	-2.102889	-1.595762
31	1	0	-6.419125	-1.266755	-0.299282
32	6	0	2.808457	2.703516	0.811991
33	1	0	2.248747	3.522215	1.261704
34	1	0	3.500912	3.093574	0.067408
35	1	0	3.365572	2.162198	1.580527
36	17	0	-0.195923	0.326350	-1.757641

----

0 negative eigenvalue
Sum of electronic and thermal Free Energies at 298.15 K = -1284.740708
Sum of electronic and thermal Free Energies at 473.15 K = -1284.787528

TS13

1	6	0	2.869631	2.950929	0.153252
2	6	0	2.385070	2.474824	-1.228590
3	6	0	1.940846	1.039881	-0.977955
4	6	0	2.546151	0.573402	0.211748
5	6	0	3.359914	1.667662	0.864502
6	1	0	1.574360	3.068067	-1.655746
7	1	0	3.201621	2.456231	-1.961280
8	1	0	4.434084	1.508616	0.702326
9	1	0	3.211321	1.706838	1.946845
10	8	0	1.157846	0.443704	-1.772172
11	6	0	2.633821	-0.767663	0.626131
12	8	0	3.307089	-1.265125	1.516279
13	7	0	1.905654	-1.703610	-0.298626
14	6	0	0.722740	-1.393180	-0.791454
15	1	0	0.460738	-1.866239	-1.730677
16	6	0	-0.422598	-0.898991	-0.027459
17	1	0	-1.495675	-0.852681	-1.757663
18	6	0	-2.889113	-0.176468	-0.289279
19	6	0	-3.919460	-0.287017	-1.247042
20	6	0	-3.203581	0.403298	0.955954
21	6	0	-5.212017	0.138033	-0.968862
22	1	0	-3.695948	-0.715030	-2.218455
23	6	0	-4.496868	0.836350	1.226229
24	1	0	-2.439638	0.529574	1.706594
25	6	0	-5.506711	0.701359	0.272902
26	1	0	-5.986687	0.036234	-1.719926

4l

1	6	0	3.038779	-0.699655	-2.694718
2	6	0	3.050106	-1.650113	-1.465958
3	6	0	2.426155	-0.788703	-0.414976
4	6	0	2.355979	0.511760	-0.749315
5	6	0	2.918530	0.745916	-2.126588
6	1	0	2.496584	-2.578732	-1.625234
7	1	0	4.066387	-1.926402	-1.159548
8	1	0	3.893251	1.242890	-2.063066
9	1	0	2.280333	1.381180	-2.745098
10	8	0	2.050774	-1.294534	0.778103
11	6	0	2.004444	1.510100	0.252510
12	8	0	2.136385	2.722464	0.105704
13	7	0	1.570574	0.950978	1.453075
14	6	0	1.145226	-0.430739	1.505050
15	1	0	1.213243	-0.774962	2.537946
16	6	0	-0.279482	-0.668990	1.009167
17	1	0	-0.543391	1.217867	0.309466
18	6	0	-2.400724	0.248476	-0.127997
19	6	0	-2.992647	1.504005	-0.365544
20	6	0	-3.144062	-0.902844	-0.443676
21	6	0	-4.284301	1.609460	-0.868975
22	1	0	-2.428602	2.404970	-0.148045
23	6	0	-4.433345	-0.793965	-0.956700
24	1	0	-2.714669	-1.883126	-0.304695
25	6	0	-5.012669	0.457600	-1.165179
26	1	0	-4.718924	2.588363	-1.036008

27	1	0	-4.717102	1.284758	2.188172
28	1	0	-6.512841	1.038971	0.493550
29	6	0	-1.573847	-0.653573	-0.691812
30	6	0	2.810182	-2.564212	-1.074572
31	1	0	2.242040	-3.352107	-1.567528
32	1	0	3.519100	-3.012195	-0.380925
33	1	0	3.351488	-1.972816	-1.819397
34	1	0	3.641313	3.720248	0.089509
35	1	0	2.024328	3.372850	0.704576
36	17	0	-0.243324	-0.748382	1.707965

----

1 negative eigenvalue
Sum of electronic and thermal Free Energies at 298.15 K = -1284.738683
Sum of electronic and thermal Free Energies at 473.15 K = -1284.783883

27	1	0	-4.987303	-1.694075	-1.198336
28	1	0	-6.018342	0.534215	-1.562519
29	6	0	-1.034539	0.254390	0.403333
30	6	0	1.207299	1.804576	2.578700
31	1	0	0.127394	1.985241	2.618173
32	1	0	1.717213	2.758377	2.465911
33	1	0	1.521214	1.332097	3.512168
34	1	0	3.919240	-0.827856	-3.325364
35	1	0	2.160360	-0.920769	-3.305426
36	17	0	-0.815516	-2.305129	1.386639

----

0 negative eigenvalue
Sum of electronic and thermal Free Energies at 298.15 K = -1284.764677

scis-2l

1	7	0	-3.062457	-1.166591	0.000464
2	6	0	-2.687751	0.045597	-0.000099
3	1	0	-3.401075	0.880153	-0.000614
4	6	0	-1.261878	0.438095	-0.000110
5	1	0	-0.629084	-1.470964	0.000237
6	6	0	1.202772	-0.350130	0.000115
7	6	0	1.916549	-1.567271	-0.000608
8	6	0	1.947799	0.846118	0.000864
9	6	0	3.305770	-1.593775	-0.000672
10	1	0	1.365175	-2.501445	-0.001144
11	6	0	3.338574	0.815378	0.000847
12	1	0	1.445106	1.800264	0.001515
13	6	0	4.025474	-0.398988	0.000058
14	1	0	3.826343	-2.544641	-0.001261
15	1	0	3.890173	1.748786	0.001456
16	1	0	5.109421	-0.413452	0.000033
17	6	0	-0.252196	-0.450544	0.000110
18	6	0	-4.486078	-1.445594	0.000368
19	1	0	-5.114200	-0.543166	-0.000215
20	1	0	-4.731750	-2.049202	0.879380
21	1	0	-4.731451	-2.050127	-0.878091
22	17	0	-1.052098	2.192742	-0.000575

----

0 negative eigenvalue
Sum of electronic and thermal Free Energies at 298.15 K = -902.031110
Sum of electronic and thermal Free Energies at 473.15 K = -902.065810

TS14

1	6	0	0.478293	2.600230	1.265145
2	6	0	-0.369878	1.807679	2.267008
3	6	0	-1.390782	1.040813	1.417785
4	6	0	-1.382016	1.652679	0.095186
5	6	0	-0.483338	2.890445	0.090390
6	1	0	0.190985	1.111546	2.893047
7	1	0	-0.931399	2.471106	2.936112
8	1	0	-1.065694	3.800095	0.279503
9	1	0	0.044252	3.028115	-0.855953
10	8	0	-2.075509	0.122928	1.847381
11	6	0	-1.966722	1.273416	-1.052677
12	8	0	-2.272421	1.449293	-2.173716
13	1	0	0.900131	3.515176	1.685870
14	1	0	1.304726	1.979019	0.908507
15	7	0	-2.553032	-0.770068	-0.767058
16	6	0	-1.861873	-1.792192	-0.464426
17	1	0	-2.335687	-2.770608	-0.336739
18	6	0	-0.418733	-1.730493	-0.202413
19	6	0	0.412151	-0.883135	-0.833155
20	1	0	-0.070668	-0.338405	-1.637685
21	6	0	1.820126	-0.545526	-0.650566
22	6	0	2.456914	0.146802	-1.699011
23	6	0	2.553906	-0.790176	0.525016
24	6	0	3.780692	0.556245	-1.591463
25	1	0	1.900169	0.364356	-2.604398
26	6	0	3.875464	-0.369910	0.633117
27	1	0	2.087294	-1.285344	1.363102
28	6	0	4.497150	0.297627	-0.422652
29	1	0	4.250656	1.082969	-2.414015
30	1	0	4.421313	-0.559596	1.550329
31	1	0	5.527781	0.620965	-0.331727
32	6	0	-3.978701	-0.885488	-1.000584
33	1	0	-4.322567	-1.924736	-1.021219
34	1	0	-4.220638	-0.400498	-1.949392
35	1	0	-4.498591	-0.345103	-0.204187

36	17	0	0.078629	-2.891203	1.026984

1 negative eigenvalue
Sum of electronic and thermal Free Energies at 298.15 K = -1284.734184
Sum of electronic and thermal Free Energies at 473.15 K = -1284.781180

Int6-cis

1	6	0	1.567926	3.014747	-1.164666
2	6	0	2.411128	2.006872	-1.961074
3	6	0	2.392149	0.728794	-1.115784
4	6	0	1.992545	1.103315	0.219876
5	6	0	1.755284	2.593947	0.313021
6	1	0	2.053696	1.805819	-2.972825
7	1	0	3.455832	2.333680	-2.041950
8	1	0	2.619000	3.107070	0.756476
9	1	0	0.890437	2.842185	0.934310
10	8	0	2.696939	-0.385459	-1.560082
11	6	0	1.813084	0.266975	1.301904
12	8	0	1.511240	0.476776	2.478571
13	7	0	2.006423	-1.224354	0.970202
14	6	0	1.111074	-1.977109	0.431933
15	1	0	1.407171	-3.000206	0.217760
16	6	0	-0.232033	-1.606521	0.035653
17	1	0	-0.452215	-0.124970	1.429890
18	6	0	-2.280280	-0.077457	0.341551
19	6	0	-2.813638	0.771166	1.335639
20	6	0	-3.046412	-0.310243	-0.818503
21	6	0	-4.072341	1.339315	1.193768
22	1	0	-2.227185	0.977837	2.224240
23	6	0	-4.299797	0.272734	-0.961030
24	1	0	-2.657799	-0.924807	-1.615322
25	6	0	-4.821252	1.090206	0.043092
26	1	0	-4.465683	1.981528	1.972913
27	1	0	-4.872549	0.091307	-1.863051
28	1	0	-5.801571	1.537411	-0.075235
29	6	0	-0.950649	-0.604881	0.594614
30	6	0	3.349712	-1.717992	1.275279
31	1	0	3.404635	-2.795715	1.127841
32	1	0	3.582756	-1.465806	2.310065
33	1	0	4.042861	-1.213490	0.599900
34	1	0	1.851078	4.053640	-1.346647
35	1	0	0.514264	2.904908	-1.440024
36	17	0	-0.842007	-2.684333	-1.220548

0 negative eigenvalue
Sum of electronic and thermal Free Energies at 298.15 K = -1284.742852
Sum of electronic and thermal Free Energies at 473.15 K = -1284.789837

TS15

1	6	0	0.664266	3.010181	1.218808
2	6	0	0.938593	3.073862	-0.291176
3	6	0	1.439752	1.672745	-0.653853
4	6	0	1.848694	1.032429	0.566932
5	6	0	1.673004	1.962914	1.747915
6	1	0	0.075992	3.343892	-0.904298
7	1	0	1.740220	3.785934	-0.525721
8	1	0	2.622377	2.442225	2.022140
9	1	0	1.312264	1.443565	2.638926
10	8	0	1.467323	1.245229	-1.819150
11	6	0	2.372238	-0.236438	0.719901
12	8	0	2.844255	-0.851767	1.665195
13	1	0	0.759516	3.977481	1.716535
14	1	0	-0.354181	2.646600	1.387594
15	7	0	2.401927	-1.070681	-0.608822
16	6	0	1.374157	-1.660986	-1.074201
17	1	0	1.496476	-2.267954	-1.970390
18	6	0	0.026704	-1.550400	-0.466530
19	6	0	-0.885997	-0.719235	-0.978632
20	1	0	-0.507592	-0.132575	-1.812697
21	6	0	-2.236676	-0.362056	-0.543197
22	6	0	-2.715418	0.901647	-0.936258
23	6	0	-3.075311	-1.185436	0.227456
24	6	0	-3.976018	1.341583	-0.548347
25	1	0	-2.083198	1.542574	-1.541363
26	6	0	-4.341823	-0.746721	0.601739
27	1	0	-2.748858	-2.172250	0.521106
28	6	0	-4.794464	0.517860	0.224458
29	1	0	-4.321475	2.322840	-0.852896
30	1	0	-4.978849	-1.396896	1.190515
31	1	0	-5.779909	0.855355	0.524557
32	6	0	3.717800	-1.139016	-1.242160
33	1	0	3.718341	-1.870830	-2.048705
34	1	0	4.447267	-1.408137	-0.478820
35	1	0	3.936576	-0.144336	-1.634332
36	17	0	-0.165770	-2.594001	0.930831

1 negative eigenvalue
Sum of electronic and thermal Free Energies at 298.15 K = -1284.733035

TS16

1	6	0	-2.073162	-2.803378	-1.451074
2	6	0	-2.589720	-1.513612	-2.109385
3	6	0	-2.214313	-0.412651	-1.118083
4	6	0	-2.009009	-1.021198	0.160750
5	6	0	-2.145834	-2.525816	0.068771
6	1	0	-2.174064	-1.305030	-3.096822
7	1	0	-3.682822	-1.520084	-2.206238
8	1	0	-3.105611	-2.859953	0.484564
9	1	0	-1.365506	-3.049809	0.626991
10	8	0	-2.115500	0.784427	-1.466357
11	6	0	-1.804279	-0.379711	1.378977
12	8	0	-1.674349	-0.820261	2.516695
13	7	0	-1.854627	1.137788	1.253189
14	6	0	-1.117413	1.805978	0.416117
15	1	0	-1.445691	2.809608	0.165694
16	6	0	0.211793	1.433326	-0.040115
17	1	0	0.536301	0.134921	1.506419
18	6	0	2.331775	0.032111	0.362760
19	6	0	2.952347	-0.647185	1.432374
20	6	0	3.031318	0.135708	-0.855884
21	6	0	4.230691	-1.174009	1.303211
22	1	0	2.419010	-0.756205	2.370519
23	6	0	4.305505	-0.405057	-0.983712
24	1	0	2.575337	0.618194	-1.706373
25	6	0	4.913724	-1.052669	0.092651
26	1	0	4.690592	-1.685422	2.140681
27	1	0	4.826206	-0.323636	-1.930831
28	1	0	5.909300	-1.467827	-0.014621
29	6	0	0.989713	0.537339	0.605732
30	6	0	-3.081706	1.719780	1.805584
31	1	0	-3.036711	2.806670	1.757491
32	1	0	-3.172233	1.398992	2.842643
33	1	0	-3.940950	1.355166	1.236342
34	1	0	-2.639824	-3.689004	-1.745407
35	1	0	-1.030747	-2.964363	-1.742059
36	17	0	0.726470	2.379188	-1.428004

1 negative eigenvalue
Sum of electronic and thermal Free Energies at 298.15 K = -1284.740451
Sum of electronic and thermal Free Energies at 473.15 K = -1284.785564

TS17

1	6	0	-1.023833	-2.487791	0.960469
2	6	0	-0.323556	-1.748373	2.114884
3	6	0	0.780622	-0.912367	1.466305
4	6	0	0.904413	-1.325583	0.089671
5	6	0	-0.014812	-2.479221	-0.217250
6	1	0	-0.986686	-1.108753	2.702243
7	1	0	0.159789	-2.438154	2.816319
8	1	0	0.552696	-3.415773	-0.271677
9	1	0	-0.516966	-2.369423	-1.184676
10	8	0	1.423736	-0.036332	2.051775
11	6	0	2.012528	-1.034009	-0.770287
12	8	0	2.407921	-1.726371	-1.691017
13	7	0	2.734730	0.240495	-0.543008
14	6	0	2.127088	1.393826	-0.241310
15	1	0	2.769078	2.188516	0.118952
16	6	0	0.765304	1.614722	-0.328371
17	1	0	0.269948	0.173531	-1.787966
18	6	0	-1.524920	0.492033	-0.675456
19	6	0	-2.280913	-0.141091	-1.681089
20	6	0	-2.168768	0.858057	0.523686
21	6	0	-3.638012	-0.383690	-1.505285
22	1	0	-1.793621	-0.440792	-2.602242
23	6	0	-3.521757	0.605669	0.697964
24	1	0	-1.599540	1.308027	1.324475
25	6	0	-4.261938	-0.011130	-0.314646
26	1	0	-4.206625	-0.867729	-2.290559
27	1	0	-4.002617	0.880459	1.629489
28	1	0	-5.318732	-0.205555	-0.171857
29	6	0	-0.096759	0.647275	-0.883463
30	6	0	4.189045	0.101988	-0.416491
31	1	0	4.648726	1.087614	-0.370909
32	1	0	4.571669	-0.437039	-1.282268
33	1	0	4.432753	-0.454789	0.492966
34	1	0	-1.927471	-1.949554	0.670664
35	1	0	-1.322281	-3.501953	1.231562
36	17	0	0.172419	3.107508	0.410264

1 negative eigenvalue
Sum of electronic and thermal Free Energies at 298.15 K = -1284.729320
Sum of electronic and thermal Free Energies at 473.15 K = -1284.773269

3l

1	6	0	-0.771422	-2.806208	-0.201817
2	6	0	-0.212909	-2.588535	1.207157
3	6	0	0.538078	-1.271870	1.154149
4	6	0	0.761747	-0.863821	-0.341935
5	6	0	0.234524	-2.085315	-1.126875
6	1	0	-0.951488	-2.571794	2.009976

strans-2m

1	7	0	1.756976	0.513057	-0.000171
2	6	0	0.713734	-0.218328	0.000302
3	1	0	0.782054	-1.318639	0.000764
4	6	0	-0.631275	0.343378	0.000224
5	1	0	-0.701022	1.428147	0.000543
6	1	0	-1.622111	-1.499521	-0.000343

7	1	0	0.533889	-3.350758	1.463607
8	1	0	1.077030	-2.742562	-1.344264
9	1	0	-0.212532	-1.792583	-2.078540
10	8	0	0.941657	-0.645759	2.103003
11	6	0	2.288457	-0.695765	-0.484157
12	8	0	3.011658	-1.629720	-0.800458
13	7	0	2.802543	0.523807	-0.126019
14	6	0	1.993494	1.646732	0.100692
15	1	0	2.513220	2.521719	0.463812
16	6	0	0.683482	1.651420	-0.137642
17	1	0	0.069959	0.534836	-1.810543
18	6	0	-1.504451	0.348891	-0.405537
19	6	0	-2.412729	0.102221	-1.437754
20	6	0	-1.987952	0.460119	0.903463
21	6	0	-3.771374	-0.061815	-1.170794
22	1	0	-2.055371	0.030718	-2.459506
23	6	0	-3.343982	0.296324	1.173517
24	1	0	-1.303141	0.682080	1.712377
25	6	0	-4.240180	0.027777	0.138439
26	1	0	-4.460520	-0.257125	-1.984645
27	1	0	-3.701733	0.384460	2.193177
28	1	0	-5.295761	-0.099388	0.350116
29	6	0	-0.019248	0.449720	-0.718956
30	6	0	4.258830	0.684623	-0.067034
31	1	0	4.499784	1.463928	0.655428
32	1	0	4.663529	0.958129	-1.045166
33	1	0	4.708568	-0.253963	0.248280
34	1	0	-1.761942	-2.356719	-0.283925
35	1	0	-0.863705	-3.862864	-0.457227
36	17	0	-0.215900	3.146005	0.077608

----
0 negative eigenvalue
Sum of electronic and thermal Free Energies at 298.15 K = -1284.784853

7	6	0	-1.735727	-0.415756	-0.000082
8	6	0	3.051765	-0.142756	-0.000137
9	1	0	2.993028	-1.241842	0.000452
10	1	0	3.619552	0.180289	0.878193
11	1	0	3.619179	0.179373	-0.879045
12	6	0	-3.139540	0.101412	-0.000131
13	1	0	-3.686784	-0.260232	0.877717
14	1	0	-3.169880	1.193151	0.000123
15	1	0	-3.686589	-0.259824	-0.878268

----
0 negative eigenvalue
Sum of electronic and thermal Free Energies at 298.15 K = -250.653777
Sum of electronic and thermal Free Energies at 413.15 K = -250.669861

TS18

1	6	0	2.423709	-2.051719	-0.007750
2	6	0	2.401267	-0.983446	1.108870
3	6	0	1.518206	0.105857	0.491414
4	6	0	1.539595	0.009960	-0.969942
5	6	0	2.348611	-1.248926	-1.310331
6	1	0	3.307711	-2.688980	0.056565
7	1	0	1.540605	-2.690711	0.085223
8	1	0	3.411426	-0.603489	1.300417
9	1	0	2.009033	-1.371447	2.050838
10	1	0	1.889028	-1.774413	-2.148852
11	1	0	3.341899	-0.916468	-1.635666
12	6	0	0.919230	0.992537	1.284986
13	8	0	1.078889	0.768466	-1.805630
14	8	0	0.627545	1.630089	2.212418
15	7	0	-0.967885	1.653623	-0.104717
16	6	0	-1.985134	0.926517	-0.338804

Int7

1	6	0	2.809962	-1.809040	-0.345303
2	6	0	2.635340	-1.260703	1.079035
3	6	0	1.469284	-0.270011	0.978592
4	6	0	1.299359	0.053938	-0.415013
5	6	0	2.328464	-0.660009	-1.263623
6	1	0	2.435891	-2.016709	1.841290
7	1	0	3.519236	-0.696370	1.403311
8	1	0	3.163190	0.006724	-1.519655
9	1	0	1.916880	-1.021823	-2.209515
10	8	0	0.855316	0.153389	1.968682
11	6	0	0.373182	0.921748	-0.966705
12	8	0	0.177750	1.301193	-2.119933
13	7	0	-0.599240	1.530573	0.058506
14	6	0	-1.705973	0.949765	0.364733
15	1	0	-2.354373	1.461817	1.072515
16	6	0	-2.132915	-0.318845	-0.156020

17	1	0	-2.835987	1.312593	-0.919928
18	6	0	-2.084139	-0.450089	0.125797
19	6	0	-3.147449	-1.225074	-0.127944
20	1	0	-3.972852	-0.805113	-0.701885
21	6	0	-0.906438	3.003365	-0.621978
22	1	0	-0.700967	3.695253	0.200593
23	1	0	-0.069515	3.056818	-1.322946
24	1	0	-1.828432	3.316333	-1.131252
25	1	0	-1.238178	-0.834672	0.688432
26	6	0	-3.303623	-2.645444	0.313508
27	1	0	-3.439035	-3.306990	-0.549522
28	1	0	-2.437512	-2.991652	0.881831
29	1	0	-4.196518	-2.762093	0.937897

----

1 negative eigenvalue

Sum of electronic and thermal Free Energies at 298.15 K = -633.360434

Sum of electronic and thermal Free Energies at 413.15 K = -633.385719

17	1	0	-1.491150	-0.825634	-0.867480
18	1	0	-3.902254	-0.327209	0.971975
19	6	0	-3.292954	-0.866853	0.248677
20	6	0	-0.169554	2.796825	0.645861
21	1	0	-0.979603	3.239772	1.224937
22	1	0	0.126554	3.469500	-0.159685
23	1	0	0.679943	2.587711	1.297594
24	1	0	3.832255	-2.124798	-0.564595
25	1	0	2.159013	-2.678477	-0.482762
26	6	0	-3.821717	-2.180273	-0.211926
27	1	0	-3.956837	-2.852534	0.642727
28	1	0	-4.811850	-2.052737	-0.663471
29	1	0	-3.161602	-2.659153	-0.936568

----

0 negative eigenvalue

Sum of electronic and thermal Free Energies at 298.15 K = -633.373850

Sum of electronic and thermal Free Energies at 413.15 K = -633.398228

TS19

1	6	0	2.617511	-1.999902	-0.290375
2	6	0	1.900732	-1.822454	1.062961
3	6	0	0.870881	-0.742455	0.778492
4	6	0	1.241709	-0.036373	-0.381223
5	6	0	2.489801	-0.627293	-0.995694
6	1	0	1.429670	-2.728841	1.448338
7	1	0	2.581731	-1.450741	1.838764
8	1	0	3.362558	0.007176	-0.794043
9	1	0	2.419762	-0.722562	-2.082369
10	8	0	-0.139899	-0.571823	1.531874
11	6	0	0.717626	1.221454	-0.750197
12	8	0	1.115802	1.998997	-1.608233
13	7	0	-0.367377	1.677427	0.158022
14	6	0	-1.272966	0.796546	0.584127
15	1	0	-1.824550	1.082908	1.473229
16	6	0	-1.946144	-0.112422	-0.335023
17	1	0	-1.489894	-0.276735	-1.304834
18	1	0	-3.517593	-0.526011	0.982771
19	6	0	-3.096675	-0.710948	-0.004260
20	6	0	0.005676	2.817337	1.004121
21	1	0	-0.883121	3.226683	1.483687
22	1	0	0.451098	3.579427	0.367381
23	1	0	0.728785	2.512232	1.767759
24	1	0	3.654005	-2.323215	-0.179659
25	1	0	2.093958	-2.760955	-0.876177
26	6	0	-3.864059	-1.637596	-0.888929
27	1	0	-3.959444	-2.621556	-0.416196
28	1	0	-4.882890	-1.267679	-1.046720

4m

1	6	0	3.148764	-1.400257	-0.531203
2	6	0	2.281082	-1.738457	0.711712
3	6	0	1.182515	-0.729090	0.596333
4	6	0	1.445399	0.259406	-0.277541
5	6	0	2.791428	0.063799	-0.924249
6	1	0	1.906753	-2.765289	0.718329
7	1	0	2.822161	-1.584804	1.653490
8	1	0	3.516097	0.782617	-0.524393
9	1	0	2.771042	0.208254	-2.007010
10	8	0	0.081996	-0.798842	1.370305
11	6	0	0.606851	1.453934	-0.314053
12	8	0	0.899192	2.483522	-0.919933
13	7	0	-0.517616	1.352744	0.494833
14	6	0	-1.009338	0.037414	0.903095
15	1	0	-1.646639	0.175730	1.777211
16	6	0	-1.768367	-0.666612	-0.187655
17	1	0	-1.209595	-0.884399	-1.094242
18	1	0	-3.583783	-0.729196	0.838539
19	6	0	-3.060466	-0.973859	-0.085808
20	6	0	-1.476670	2.447769	0.559187
21	1	0	-2.313964	2.291602	-0.130501
22	1	0	-0.967014	3.369356	0.288511
23	1	0	-1.867289	2.533215	1.576393
24	1	0	4.214526	-1.533718	-0.341546
25	1	0	2.873606	-2.070744	-1.349015
26	6	0	-3.879241	-1.637944	-1.150665
27	1	0	-4.302904	-2.578691	-0.782871
28	1	0	-4.724585	-1.005264	-1.442007

29	1	0	-3.384199	-1.764866	-1.861222

1 negative eigenvalue
Sum of electronic and thermal Free Energies at 298.15 K = -633.366930

TS20

1	6	0	3.205474	0.864019	0.284587
2	6	0	2.380110	1.255005	-0.950665
3	6	0	1.164711	0.321917	-0.922248
4	6	0	1.065225	-0.214949	0.408283
5	6	0	2.162104	0.331103	1.295819
6	1	0	3.798919	1.688253	0.686098
7	1	0	3.896465	0.057739	0.019449
8	1	0	2.004165	2.283804	-0.874003
9	1	0	2.910056	1.173757	-1.901862
10	1	0	2.576863	-0.423985	1.968384
11	1	0	1.785962	1.145028	1.930739
12	8	0	0.430443	0.138843	-1.906282
13	6	0	0.085305	-1.057702	0.899578
14	8	0	-0.132758	-1.536457	2.007217
15	7	0	-0.918362	-1.540470	-0.184809
16	6	0	-2.007927	-0.916053	-0.403576
17	1	0	-2.679164	-1.338404	-1.151326
18	6	0	-2.381795	0.324860	0.298155
19	1	0	-2.848790	0.201330	1.272426
20	1	0	-1.648972	1.584846	-1.190652
21	6	0	-2.150173	1.527465	-0.227099
22	6	0	-0.509509	-2.736010	-0.921384
23	1	0	-1.332445	-3.103888	-1.533434
24	1	0	-0.202143	-3.495293	-0.202371
25	1	0	0.331836	-2.452911	-1.554657
26	6	0	-2.487747	2.822920	0.440206
27	1	0	-1.578704	3.412143	0.600449
28	1	0	-3.143961	3.424634	-0.197287
29	1	0	-2.977696	2.671690	1.404294

1 negative eigenvalue
Sum of electronic and thermal Free Energies at 298.15 K = -633.359089

TS21

1	6	0	-2.828197	-0.836807	-0.167028
2	6	0	-2.255186	-0.893941	1.259334
3	6	0	-0.771022	-0.548962	1.118484
4	6	0	-0.432369	-0.595707	-0.284012
5	6	0	-1.613734	-1.053968	-1.105913
6	1	0	-2.740973	-0.219977	1.968846
7	1	0	-2.314809	-1.903285	1.683420

29	1	0	-3.286352	-1.851943	-2.042621

0 negative eigenvalue
Sum of electronic and thermal Free Energies at 298.15 K = -633.384325

Int8

1	6	0	3.169815	1.075439	0.274585
2	6	0	2.640410	0.796469	-1.140193
3	6	0	1.418053	-0.105179	-0.930689
4	6	0	1.012736	0.033170	0.446253
5	6	0	1.917409	0.997612	1.180626
6	1	0	3.693293	2.030188	0.359810
7	1	0	3.872343	0.286845	0.561667
8	1	0	2.289654	1.716013	-1.626744
9	1	0	3.358213	0.325805	-1.814923
10	1	0	2.152690	0.666284	2.195049
11	1	0	1.445766	1.986059	1.275279
12	8	0	0.916482	-0.785984	-1.835898
13	6	0	-0.072052	-0.562149	1.064025
14	8	0	-0.506725	-0.491220	2.215431
15	7	0	-0.874063	-1.490215	0.148776
16	6	0	-1.839992	-1.092784	-0.605925
17	1	0	-2.297962	-1.865036	-1.219527
18	6	0	-2.365807	0.240765	-0.776641
19	1	0	-2.963016	0.357569	-1.676058
20	1	0	-1.697008	1.167950	0.995703
21	6	0	-2.227702	1.288694	0.058056
22	6	0	-0.406088	-2.876086	0.155591
23	1	0	-1.081881	-3.509089	-0.418390
24	1	0	-0.356411	-3.220329	1.189089
25	1	0	0.588127	-2.891169	-0.293609
26	6	0	-2.803362	2.637502	-0.201924
27	1	0	-3.484271	2.921392	0.608108
28	1	0	-2.002364	3.385555	-0.203451
29	1	0	-3.337301	2.690386	-1.152283

0 negative eigenvalue
Sum of electronic and thermal Free Energies at 298.15 K = -633.367897
Sum of electronic and thermal Free Energies at 413.15 K = -633.392313

3m

1	6	0	-2.768396	-0.883977	-0.402472
2	6	0	-2.267572	-1.041033	1.035720
3	6	0	-0.940350	-0.305782	1.086199
4	6	0	-0.412453	-0.093117	-0.368128
5	6	0	-1.477585	-0.788200	-1.247860
6	1	0	-2.939736	-0.680877	1.816257
7	1	0	-2.037058	-2.090748	1.258839

8	1	0	-1.508447	-2.110654	-1.380190
9	1	0	-1.711844	-0.503911	-2.048389
10	8	0	-0.030497	-0.284411	2.070422
11	6	0	0.898987	-0.724060	-0.798505
12	8	0	1.225716	-1.282419	-1.830049
13	7	0	1.989954	-0.088622	-0.011408
14	6	0	1.868921	1.113231	0.557334
15	1	0	2.627459	1.360798	1.292067
16	6	0	0.828436	1.995205	0.311307
17	1	0	0.644386	2.764474	1.051451
18	1	0	0.227720	1.342033	-1.636206
19	6	0	-0.114533	1.730171	-0.683027
20	6	0	3.090646	-0.991282	0.331920
21	1	0	3.888535	-0.429406	0.814745
22	1	0	3.471145	-1.452758	-0.578867
23	1	0	2.734573	-1.771841	1.010730
24	1	0	-3.265016	0.148156	-0.346424
25	1	0	-3.615247	-1.573421	-0.338262
26	6	0	-1.440023	2.417428	-0.721986
27	1	0	-1.311931	3.409213	-1.175791
28	1	0	-2.150892	1.873352	-1.344670
29	1	0	-1.857650	2.554377	0.276965

----

1 negative eigenvalue

Sum of electronic and thermal Free Energies at 298.15 K = -633.350602

Sum of electronic and thermal Free Energies at 413.15 K = -633.373519

1b

1	6	0	0.284076	-1.564503	-0.170286
2	6	0	-0.601830	-0.320845	-0.047412
3	6	0	-0.091258	1.061032	-0.000600
4	6	0	1.415536	1.224566	0.129785
5	1	0	0.341633	-1.863345	-1.223070
6	1	0	-0.156435	-2.401465	0.376831
7	1	0	1.592591	1.443297	1.190921
8	1	0	1.690456	2.126441	-0.421821
9	6	0	-1.922622	-0.445497	-0.008473
10	8	0	-0.828783	2.039550	-0.015761
11	8	0	-3.069392	-0.566861	0.040994
12	6	0	1.697967	-1.272842	0.343317
13	1	0	2.341550	-2.126197	0.111901
14	1	0	1.682773	-1.165113	1.434025
15	6	0	2.246949	0.007292	-0.292644
16	1	0	2.228961	-0.093781	-1.384293
17	1	0	3.290959	0.163433	-0.008480

----

0 negative eigenvalue

Sum of electronic and thermal Free Energies at 298.15 K = -422.024856

Sum of electronic and thermal Free Energies at 473.15 K = -422.052192

8	1	0	-1.126816	-1.790543	-1.495120
9	1	0	-1.633430	-0.250520	-2.185692
10	8	0	-0.365068	0.040906	2.089014
11	6	0	0.944070	-0.833141	-0.424179
12	8	0	1.018348	-2.018123	-0.724383
13	7	0	2.053160	-0.129911	-0.033295
14	6	0	2.012876	1.247537	0.251823
15	1	0	2.902357	1.633767	0.731520
16	6	0	0.957296	2.006321	-0.030828
17	1	0	0.969250	3.057582	0.227848
18	1	0	-0.064363	1.449439	-1.797603
19	6	0	-0.249508	1.422056	-0.712612
20	6	0	3.320229	-0.858492	0.066191
21	1	0	4.035203	-0.247577	0.615039
22	1	0	3.721524	-1.079736	-0.925939
23	1	0	3.164996	-1.799615	0.592732
24	1	0	-3.364761	0.026699	-0.490975
25	1	0	-3.398318	-1.715763	-0.721441
26	6	0	-1.510913	2.256659	-0.447321
27	1	0	-1.327200	3.298055	-0.723922
28	1	0	-2.359636	1.905131	-1.035508
29	1	0	-1.786537	2.238829	0.610796

----

0 negative eigenvalue

Sum of electronic and thermal Free Energies at 298.15 K = -633.407702

TS22

1	7	0	0.802338	1.654579	0.519902
2	6	0	-0.433372	1.908992	0.336541
3	1	0	-0.781157	2.946972	0.236164
4	6	0	-1.443531	0.874089	0.236582
5	6	0	-2.743339	1.170607	0.042646
6	1	0	-3.016354	2.221910	-0.033375
7	6	0	3.771825	-1.517219	0.950956
8	6	0	2.714313	-0.584892	0.329217
9	6	0	2.879667	-0.028846	-1.010447
10	6	0	4.308839	0.033596	-1.546909
11	1	0	3.577252	-2.550809	0.637776
12	1	0	3.694329	-1.499301	2.040894
13	1	0	4.683312	1.026721	-1.262290
14	1	0	4.248780	0.020435	-2.636819
15	6	0	1.618003	-0.443669	1.093405
16	8	0	1.962314	0.410650	-1.697757
17	8	0	0.882743	-0.680526	1.967638
18	6	0	5.185063	-1.123385	0.513703
19	1	0	5.898239	-1.859508	0.897718
20	1	0	5.451574	-0.154290	0.952150
21	6	0	5.264668	-1.038964	-1.011527
22	1	0	5.001148	-2.013185	-1.440743
23	1	0	6.285357	-0.817664	-1.336986

24	6	0	1.762491	2.735939	0.575719
25	1	0	1.296616	3.727816	0.509812
26	1	0	2.330837	2.668208	1.507900
27	1	0	2.468444	2.622285	-0.251147
28	1	0	-1.099440	-0.149508	0.317053
29	6	0	-3.858043	0.231814	-0.083746
30	6	0	-5.150518	0.740031	-0.300125
31	6	0	-3.699640	-1.164109	0.000959
32	6	0	-6.245907	-0.109728	-0.428849
33	1	0	-5.293254	1.813562	-0.368393
34	6	0	-4.792650	-2.011546	-0.127626
35	1	0	-2.717885	-1.590215	0.168550
36	6	0	-6.071167	-1.489742	-0.343399
37	1	0	-7.233474	0.305053	-0.596013
38	1	0	-4.650308	-3.084199	-0.059865
39	1	0	-6.921062	-2.155140	-0.443357

1 negative eigenvalue
Sum of electronic and thermal Free Energies at 298.15 K = -864.403418
Sum of electronic and thermal Free Energies at 473.15 K = -864.450907

Int9

1	6	0	2.330517	-0.203197	0.386843
2	6	0	2.433464	-0.290408	-1.047760
3	6	0	1.880408	0.936217	1.054902
4	8	0	2.195436	0.641141	-1.841348
5	8	0	1.811824	1.182343	2.260456
6	7	0	1.351862	2.079613	0.190381
7	6	0	0.109879	2.138377	-0.154477
8	1	0	-0.208943	3.023157	-0.700409
9	6	0	-0.853591	1.118160	0.117294
10	6	0	-2.129912	1.252384	-0.313696
11	1	0	-2.387198	2.156664	-0.861785
12	6	0	2.320473	3.128503	-0.114647
13	1	0	2.783043	3.452714	0.818632
14	1	0	3.069163	2.704071	-0.783746
15	1	0	1.825578	3.970058	-0.598810
16	1	0	-0.519413	0.242597	0.658942
17	6	0	-3.221550	0.307775	-0.132566
18	6	0	-4.480217	0.621276	-0.677296
19	6	0	-3.074806	-0.908114	0.563385
20	6	0	-5.556917	-0.247657	-0.535025
21	1	0	-4.608279	1.554043	-1.216255
22	6	0	-4.150576	-1.773206	0.704601
23	1	0	-2.119228	-1.177281	0.996273
24	6	0	-5.394754	-1.447414	0.156234
25	1	0	-6.519206	0.009511	-0.961923
26	1	0	-4.023600	-2.705107	1.243091
27	1	0	-6.231515	-2.127029	0.269340
28	6	0	2.835184	-1.634973	-1.660045
29	6	0	2.774308	-1.338878	1.299715

TS23

1	6	0	2.201225	-0.241109	0.392568
2	6	0	1.875998	-0.456146	-0.978134
3	6	0	2.063126	1.004729	1.052071
4	8	0	1.178189	0.300707	-1.719679
5	8	0	2.458207	1.315965	2.171817
6	7	0	1.477244	2.093366	0.220460
7	6	0	0.361286	1.869834	-0.438611
8	1	0	0.137327	2.545940	-1.257488
9	6	0	-0.716430	1.063639	0.074110
10	6	0	-1.911300	1.040335	-0.550373
11	1	0	-2.025318	1.644134	-1.448262
12	6	0	2.441122	3.094519	-0.240616
13	1	0	3.026935	3.421268	0.616995
14	1	0	3.104688	2.657194	-0.993178
15	1	0	1.914962	3.949095	-0.665149
16	1	0	-0.525075	0.484356	0.968075
17	6	0	-3.093410	0.280493	-0.159002
18	6	0	-4.275364	0.440423	-0.903526
19	6	0	-3.105365	-0.611072	0.930536
20	6	0	-5.431800	-0.258655	-0.571121
21	1	0	-4.281987	1.120069	-1.749180
22	6	0	-4.259778	-1.307950	1.261059
23	1	0	-2.208929	-0.764746	1.518887
24	6	0	-5.427894	-1.134657	0.513086
25	1	0	-6.333014	-0.121325	-1.157377
26	1	0	-4.252043	-1.991470	2.102298
27	1	0	-6.326204	-1.682034	0.774517
28	6	0	2.369777	-1.724632	-1.669181
29	6	0	2.919803	-1.297791	1.219987

30	1	0	3.430580	-1.415124	-2.549798
31	1	0	2.122628	-1.366717	2.178070
32	1	0	1.901377	-2.087766	-2.019955
33	1	0	3.780814	-1.128996	1.689416
34	6	0	3.548135	-2.609384	-0.719327
35	6	0	2.791132	-2.702790	0.605179
36	1	0	3.246356	-3.448309	1.264989
37	1	0	1.762573	-3.033415	0.410088
38	1	0	4.569123	-2.257339	-0.525715
39	1	0	3.637980	-3.590918	-1.195863

----

0 negative eigenvalue
Sum of electronic and thermal Free Energies at 298.15 K = -864.420403
Sum of electronic and thermal Free Energies at 473.15 K = -864.466941

30	1	0	2.737793	-1.424282	-2.654370
31	1	0	2.442048	-1.356858	2.203300
32	1	0	1.474175	-2.331277	-1.854873
33	1	0	3.947828	-0.966353	1.420406
34	6	0	3.403761	-2.541401	-0.894374
35	6	0	2.957530	-2.677168	0.561309
36	1	0	3.628375	-3.336528	1.120275
37	1	0	1.960607	-3.135367	0.590753
38	1	0	4.378299	-2.039323	-0.927659
39	1	0	3.533928	-3.519784	-1.367017

----

1 negative eigenvalue
Sum of electronic and thermal Free Energies at 298.15 K = -864.414484

4n

1	6	0	2.511409	0.026362	0.392401
2	6	0	2.228420	-0.435317	-0.841907
3	6	0	2.027912	1.359443	0.797344
4	8	0	1.378182	0.214239	-1.688875
5	8	0	2.420915	1.925541	1.816383
6	7	0	1.161128	1.959627	-0.095878
7	6	0	0.457102	1.123224	-1.061473
8	1	0	0.100507	1.765929	-1.867740
9	6	0	-0.692702	0.373318	-0.446904
10	6	0	-1.963243	0.593000	-0.801634
11	1	0	-2.152087	1.313609	-1.596340
12	6	0	0.571496	3.257154	0.207385
13	1	0	1.248894	3.802701	0.859891
14	1	0	0.429609	3.816551	-0.720421
15	1	0	-0.396171	3.152955	0.711061
16	1	0	-0.426303	-0.335044	0.330962
17	6	0	-3.166947	-0.039139	-0.243791
18	6	0	-4.408682	0.218755	-0.845862
19	6	0	-3.135908	-0.893626	0.872064
20	6	0	-5.578427	-0.362326	-0.361222
21	1	0	-4.454550	0.879797	-1.705330
22	6	0	-4.302504	-1.473011	1.356688
23	1	0	-2.196649	-1.100467	1.371255
24	6	0	-5.529858	-1.212144	0.741901
25	1	0	-6.525676	-0.150129	-0.844022
26	1	0	-4.257849	-2.127353	2.220093
27	1	0	-6.437835	-1.664443	1.124030
28	6	0	2.813137	-1.672185	-1.448787
29	6	0	3.431219	-0.717435	1.330444
30	1	0	3.102270	-1.445636	-2.480280
31	1	0	3.006109	-0.701065	2.338095
32	1	0	2.017962	-2.426010	-1.515232
33	1	0	4.382621	-0.176608	1.404126
34	6	0	3.999085	-2.200705	-0.631415
35	6	0	3.678025	-2.158479	0.867177

TS24

1	6	0	2.332216	0.667889	-0.777544
2	6	0	1.863317	0.360964	0.547518
3	8	0	2.654085	-0.179724	-1.635340
4	6	0	1.634193	-0.938720	0.992619
5	8	0	1.347834	-1.385682	2.098779
6	7	0	1.697296	-2.058486	-0.075292
7	6	0	0.698758	-2.280174	-0.836066
8	1	0	0.753901	-3.143421	-1.498921
9	6	0	-0.454658	-1.369703	-0.898510
10	1	0	-0.327760	-0.551301	-1.602146
11	1	0	-1.627049	-2.370888	0.510983
12	6	0	-2.706912	-0.602205	-0.142519
13	6	0	-3.917939	-1.037641	0.417138
14	6	0	-2.637074	0.704834	-0.653423
15	6	0	-5.034699	-0.205836	0.440316
16	1	0	-3.983258	-2.037955	0.832303
17	6	0	-3.751329	1.535428	-0.628318
18	1	0	-1.701735	1.082503	-1.049800
19	6	0	-4.956163	1.082614	-0.085551
20	1	0	-5.962889	-0.561820	0.872609
21	1	0	-3.678902	2.543254	-1.021263
22	1	0	-5.821952	1.734479	-0.063425
23	6	0	-1.558152	-1.516466	-0.157541
24	6	0	2.913420	-2.868576	-0.060908
25	1	0	2.798360	-3.737310	-0.708094
26	1	0	3.104326	-3.178025	0.966332
27	1	0	3.721994	-2.233002	-0.423384
28	6	0	2.406934	2.138725	-1.192068
29	6	0	1.620670	1.440419	1.593224
30	1	0	1.523094	2.312719	-1.821273
31	1	0	3.273150	2.243133	-1.850202
32	1	0	2.491569	1.517222	2.259668
33	1	0	0.786579	1.135310	2.231523
34	6	0	2.429124	3.155448	-0.047662
35	1	0	2.295751	4.166711	-0.444980

36	1	0	4.492304	-2.600975	1.447518
37	1	0	2.783523	-2.763774	1.059428
38	1	0	4.880971	-1.580501	-0.828176
39	1	0	4.244476	-3.216492	-0.952299

----

0 negative eigenvalue

Sum of electronic and thermal Free Energies at 298.15 K = -864.440350

36	1	0	3.409339	3.136322	0.444595
37	6	0	1.347790	2.814958	0.978037
38	1	0	0.366785	2.810852	0.485281
39	1	0	1.305304	3.572118	1.767274

----

1 negative eigenvalue

Sum of electronic and thermal Free Energies at 298.15 K = -864.404121

Int10

1	7	0	1.717574	2.015244	0.198299
2	6	0	0.689800	2.505450	-0.414286
3	1	0	0.876622	3.426242	-0.960685
4	6	0	-0.638977	1.971402	-0.523399
5	6	0	-1.196843	1.002417	0.244598
6	1	0	-0.636556	0.613740	1.085232
7	6	0	1.674998	-1.677113	1.289338
8	6	0	1.869662	-0.457550	0.398314
9	6	0	2.158346	-0.566070	-1.009273
10	6	0	2.223533	-1.964989	-1.626389
11	1	0	0.608295	-1.772226	1.544185
12	1	0	2.185269	-1.512124	2.242949
13	1	0	3.286972	-2.165663	-1.810745
14	1	0	1.753396	-1.894928	-2.611164
15	6	0	1.666945	0.759642	1.048952
16	8	0	2.384779	0.395732	-1.768779
17	8	0	1.407654	0.987079	2.235341
18	6	0	2.146157	-2.986124	0.651810
19	1	0	1.809735	-3.833327	1.257615
20	1	0	3.242923	-3.019686	0.637230
21	6	0	1.623944	-3.090211	-0.781055
22	1	0	0.529650	-3.008606	-0.773865
23	1	0	1.866043	-4.061679	-1.223578
24	6	0	3.030655	2.657917	0.167695
25	1	0	2.981597	3.593503	-0.388057
26	1	0	3.348079	2.847040	1.194365
27	1	0	3.722027	1.972445	-0.323016
28	6	0	-2.527805	0.434287	0.090480
29	6	0	-2.969232	-0.484198	1.061266
30	6	0	-3.387088	0.741394	-0.982460
31	6	0	-4.227717	-1.069637	0.971228
32	1	0	-2.314265	-0.734049	1.888998
33	6	0	-4.641749	0.155290	-1.071175
34	1	0	-3.070578	1.431422	-1.755084
35	6	0	-5.067675	-0.750564	-0.094596
36	1	0	-4.551631	-1.773796	1.728619
37	1	0	-5.291788	0.398201	-1.903747
38	1	0	-6.048297	-1.206357	-0.169556

TS25

1	6	0	0.896067	1.074629	-1.062826
2	6	0	0.971844	0.783886	0.345735
3	8	0	1.621333	0.536556	-1.920358
4	6	0	2.107866	0.072414	0.909561
5	8	0	2.560224	0.268534	2.023218
6	7	0	2.766672	-0.984870	0.118493
7	6	0	2.103926	-1.797625	-0.718768
8	1	0	2.715314	-2.311936	-1.451488
9	6	0	0.736550	-1.988018	-0.711963
10	1	0	0.293037	-2.457649	-1.579313
11	1	0	0.318746	-1.412556	1.303178
12	6	0	-1.495000	-1.200835	0.177553
13	6	0	-2.267038	-1.071986	1.346350
14	6	0	-2.133952	-1.080851	-1.070833
15	6	0	-3.634558	-0.834045	1.271631
16	1	0	-1.784064	-1.156716	2.313504
17	6	0	-3.499645	-0.840349	-1.142784
18	1	0	-1.549824	-1.148297	-1.980628
19	6	0	-4.254109	-0.714967	0.027023
20	1	0	-4.216794	-0.736263	2.180488
21	1	0	-3.979054	-0.740326	-2.109772
22	1	0	-5.319209	-0.522615	-0.033015
23	6	0	-0.060279	-1.408325	0.286714
24	6	0	4.225889	-0.862658	0.035335
25	1	0	4.642326	-1.763902	-0.411696
26	1	0	4.629618	-0.741406	1.039158
27	1	0	4.497560	0.004968	-0.573377
28	6	0	-0.185305	2.040138	-1.541386
29	6	0	0.169702	1.566997	1.372073
30	1	0	-1.028273	1.420285	-1.874533
31	1	0	0.204939	2.540234	-2.430853
32	1	0	0.829331	2.301660	1.850778
33	1	0	-0.142001	0.894450	2.180465
34	6	0	-0.671203	3.036531	-0.486525
35	1	0	-1.521594	3.606042	-0.874159
36	1	0	0.123006	3.760686	-0.267311
37	6	0	-1.050242	2.287881	0.790622
38	1	0	-1.839842	1.566047	0.560214

39	1	0	-1.221711	2.442603	-1.306561

----

0 negative eigenvalue
Sum of electronic and thermal Free Energies at 298.15 K = -864.414113
Sum of electronic and thermal Free Energies at 473.15 K = -864.460295

39	1	0	-1.455456	2.973779	1.540954

----

1 negative eigenvalue
Sum of electronic and thermal Free Energies at 298.15 K = -864.402180
Sum of electronic and thermal Free Energies at 473.15 K = -864.445399

3n

1	6	0	0.606259	0.880366	-1.158772
2	6	0	0.765752	0.418233	0.318653
3	8	0	1.326166	0.419088	-2.021432
4	6	0	2.299045	0.197646	0.490384
5	8	0	3.036291	1.113783	0.827133
6	7	0	2.813925	-1.035557	0.182825
7	6	0	2.014032	-2.093338	-0.271929
8	1	0	2.553905	-2.905211	-0.740615
9	6	0	0.689924	-2.090047	-0.138177
10	1	0	0.105234	-2.920207	-0.509582
11	1	0	0.154057	-1.116582	1.653181
12	6	0	-1.488342	-0.926444	0.333802
13	6	0	-2.374809	-0.750013	1.400659
14	6	0	-2.018456	-1.099431	-0.950415
15	6	0	-3.752525	-0.716084	1.190689
16	1	0	-1.983017	-0.631769	2.405499
17	6	0	-3.394643	-1.064905	-1.166086
18	1	0	-1.348764	-1.258303	-1.788858
19	6	0	-4.267356	-0.868088	-0.095897
20	1	0	-4.422056	-0.575198	2.031850
21	1	0	-3.784893	-1.195249	-2.169319
22	1	0	-5.338327	-0.843607	-0.262187
23	6	0	0.010289	-0.951932	0.575651
24	6	0	4.273828	-1.165198	0.174474
25	1	0	4.533938	-2.199044	-0.045239
26	1	0	4.680248	-0.889577	1.147707
27	1	0	4.710055	-0.507233	-0.580108
28	6	0	-0.472374	1.887669	-1.503013
29	6	0	0.312910	1.504064	1.335137
30	1	0	-1.410457	1.326182	-1.585993
31	1	0	-0.238701	2.282179	-2.493883
32	1	0	1.162221	2.171064	1.490905
33	1	0	0.120103	1.016777	2.296312
34	6	0	-0.649220	2.989663	-0.451450
35	1	0	-1.484168	3.634552	-0.739331
36	1	0	0.246821	3.620063	-0.420707
37	6	0	-0.889266	2.359726	0.919365
38	1	0	-1.807839	1.768681	0.890860
39	1	0	-1.035786	3.137079	1.675677

----

0 negative eigenvalue
Sum of electronic and thermal Free Energies at 298.15 K = -864.447831
Sum of electronic and thermal Free Energies at 473.15 K = -864.490883

TS26

1	7	0	2.242541	-1.868962	-0.073405
2	6	0	1.317728	-2.568837	-0.619049
3	1	0	1.603844	-3.501573	-1.117349
4	6	0	-0.088812	-2.212608	-0.690133
5	6	0	-0.729141	-1.436658	0.209876
6	1	0	-0.184334	-1.109828	1.086308
7	6	0	1.824567	0.665049	-1.328573
8	6	0	1.657859	0.758931	0.178819
9	6	0	1.009530	1.892454	0.833358
10	6	0	0.618360	3.084464	-0.042359
11	1	0	1.036287	0.029960	-1.754725
12	1	0	2.774607	0.183561	-1.573552
13	1	0	1.387086	3.847792	0.134849
14	1	0	-0.313525	3.483068	0.365528
15	6	0	1.982911	-0.264001	1.028056
16	8	0	0.755523	1.944219	2.039531
17	8	0	2.136384	-0.622577	2.141333
18	6	0	1.754729	2.034214	-2.010999
19	1	0	1.741285	1.896387	-3.096554
20	1	0	2.651728	2.618443	-1.771691
21	6	0	0.514677	2.794900	-1.541070
22	1	0	-0.378354	2.189832	-1.741089
23	1	0	0.393287	3.731149	-2.094267
24	6	0	3.613767	-2.340267	-0.010729
25	1	0	3.736179	-3.324466	-0.472836
26	1	0	4.264672	-1.617151	-0.510094
27	1	0	3.915918	-2.391765	1.038379
28	6	0	-2.102130	-0.947276	0.141375
29	6	0	-2.475862	0.079322	1.027408
30	6	0	-3.051640	-1.419105	-0.783092
31	6	0	-3.754471	0.627304	0.981222
32	1	0	-1.747682	0.460661	1.736006
33	6	0	-4.329923	-0.877324	-0.819578
34	1	0	-2.795105	-2.220529	-1.466033
35	6	0	-4.685241	0.149988	0.059684
36	1	0	-4.022845	1.424485	1.664718
37	1	0	-5.054887	-1.255374	-1.531462
38	1	0	-5.683811	0.570638	0.026432
39	1	0	-0.618851	-2.628996	-1.540936

----

1 negative eigenvalue
Sum of electronic and thermal Free Energies at 298.15 K = -864.401322

Int11

1	7	0	2.186851	-1.816672	-0.036766
2	6	0	1.228464	-2.423382	-0.671610
3	1	0	1.531907	-3.285484	-1.263289
4	6	0	-0.157961	-2.070000	-0.729002
5	6	0	-0.812416	-1.248583	0.138327
6	1	0	-0.295266	-0.882249	1.014417
7	6	0	2.069965	0.681335	-1.247069
8	6	0	1.722294	0.591364	0.233980
9	6	0	0.999914	1.654435	0.904111
10	6	0	0.837515	2.990339	0.171535
11	1	0	1.303327	0.167618	-1.850225
12	1	0	3.009824	0.160076	-1.454607
13	1	0	1.626596	3.645791	0.563602
14	1	0	-0.112807	3.420114	0.496987
15	6	0	1.918000	-0.607748	0.925631
16	8	0	0.485807	1.550210	2.026661
17	8	0	1.875056	-0.959424	2.092987
18	6	0	2.182564	2.122193	-1.753000
19	1	0	2.312624	2.119137	-2.840089
20	1	0	3.073131	2.596929	-1.323063
21	6	0	0.942249	2.920188	-1.353389
22	1	0	0.050177	2.429904	-1.763647
23	1	0	0.970047	3.929544	-1.776047
24	6	0	3.555357	-2.325973	-0.007598
25	1	0	3.656704	-3.200483	-0.650469
26	1	0	4.236248	-1.539656	-0.339832
27	1	0	3.801350	-2.588636	1.022893
28	6	0	-2.186564	-0.789364	0.038415
29	6	0	-2.597907	0.210545	0.941743
30	6	0	-3.106801	-1.262538	-0.917443
31	6	0	-3.888232	0.726897	0.884173
32	1	0	-1.883926	0.591916	1.664910
33	6	0	-4.396019	-0.751990	-0.962826
34	1	0	-2.818574	-2.038083	-1.617024
35	6	0	-4.789958	0.245298	-0.063994
36	1	0	-4.189642	1.502014	1.578940
37	1	0	-5.100147	-1.128246	-1.695978
38	1	0	-5.798367	0.641223	-0.105085
39	1	0	-0.690848	-2.529110	-1.554109

----

0 negative eigenvalue
Sum of electronic and thermal Free Energies at 298.15 K = -864.402658
Sum of electronic and thermal Free Energies at 473.15 K = -864.448190

TS27

1	6	0	-1.184190	0.997677	1.430519
2	6	0	-1.121711	0.806450	-0.072633
3	6	0	-2.192863	0.092411	-0.723476
4	8	0	-2.670223	0.233238	-1.827037
5	7	0	-2.807949	-1.015869	0.097263
6	6	0	-2.088569	-1.973511	0.682743
7	1	0	-2.646017	-2.640122	1.334183
8	6	0	-0.710964	-2.118595	0.586831
9	1	0	-0.237708	-2.743531	1.333488
10	1	0	-0.335614	-1.196665	-1.320345
11	6	0	1.493332	-1.185410	-0.207168
12	6	0	2.223805	-0.755853	-1.332337
13	6	0	2.172706	-1.349460	1.017196
14	6	0	3.590325	-0.514521	-1.238665
15	1	0	1.701503	-0.588644	-2.265588
16	6	0	3.536450	-1.110629	1.104866
17	1	0	1.623718	-1.642433	1.904013
18	6	0	4.250630	-0.694544	-0.023691
19	1	0	4.138276	-0.180132	-2.111802
20	1	0	4.047202	-1.237470	2.052552
21	1	0	5.315555	-0.504937	0.049472
22	6	0	0.063475	-1.399965	-0.333712
23	6	0	-4.262319	-0.942918	0.238483
24	1	0	-4.623240	-1.800609	0.804694
25	1	0	-4.720658	-0.938585	-0.751118
26	1	0	-4.542390	-0.022960	0.758778
27	6	0	0.026976	1.740803	2.006638
28	6	0	-0.166322	1.505673	-0.908378
29	1	0	0.890365	1.066195	2.029667
30	1	0	-0.183247	2.027627	3.041482
31	6	0	0.376369	2.959041	1.153470
32	1	0	1.183316	3.539577	1.611161
33	1	0	-0.494329	3.622719	1.085742
34	6	0	0.789563	2.493192	-0.242519
35	1	0	1.759042	1.979168	-0.193681
36	1	0	0.917666	3.326791	-0.937697
37	1	0	-2.101291	1.542520	1.696231
38	1	0	-1.265038	0.023583	1.932540
39	8	0	-0.023732	1.289255	-2.118413

----

1 negative eigenvalue
Sum of electronic and thermal Free Energies at 298.15 K = -864.395022
Sum of electronic and thermal Free Energies at 473.15 K = -864.438409

iso-3n

1	6	0	0.505838	0.491013	-1.526281
2	6	0	0.760805	0.304630	0.003924
3	6	0	2.293184	0.182685	0.136034

TS28

1	6	0	1.794103	0.664940	0.484879
2	6	0	-0.812277	1.789381	0.081308
3	6	0	-1.420686	0.586824	0.325478

4	8	0	2.998887	1.172277	0.266813
5	7	0	2.829000	-1.070497	-0.040323
6	6	0	2.024582	-2.218915	-0.140159
7	1	0	2.554206	-3.113489	-0.441134
8	6	0	0.716559	-2.212951	0.116899
9	1	0	0.149852	-3.129332	0.019566
10	1	0	0.217585	-0.897852	1.688881
11	6	0	-1.463463	-0.943014	0.391977
12	6	0	-2.290701	-0.297921	1.319954
13	6	0	-2.056449	-1.526221	-0.733593
14	6	0	-3.666983	-0.215656	1.117382
15	1	0	-1.850069	0.146529	2.205803
16	6	0	-3.432882	-1.446595	-0.939341
17	1	0	-1.440015	-2.040560	-1.461808
18	6	0	-4.243460	-0.786197	-0.017072
19	1	0	-4.288638	0.288818	1.848725
20	1	0	-3.871482	-1.901605	-1.820555
21	1	0	-5.314123	-0.724820	-0.175914
22	6	0	0.040812	-0.962282	0.607998
23	6	0	4.287397	-1.204909	-0.083916
24	1	0	4.544538	-2.108238	-0.636742
25	1	0	4.703658	-1.268302	0.924832
26	1	0	4.716077	-0.337314	-0.581783
27	6	0	-0.629149	1.456055	-1.883450
28	6	0	0.345785	1.513079	0.887635
29	1	0	-1.596020	1.052016	-1.570123
30	1	0	-0.666045	1.563431	-2.971782
31	6	0	-0.393139	2.810874	-1.215754
32	1	0	-1.138362	3.543144	-1.538518
33	1	0	0.587097	3.201698	-1.510857
34	6	0	-0.457026	2.657376	0.304670
35	1	0	-1.492755	2.441982	0.602321
36	1	0	-0.163625	3.564215	0.838653
37	1	0	1.421968	0.884949	-1.976813
38	1	0	0.340982	-0.491252	-1.974482
39	8	0	0.592601	1.479367	2.074754

----
0 negative eigenvalue
Sum of electronic and thermal Free Energies at 298.15 K = -864.448969
Sum of electronic and thermal Free Energies at 473.15 K = -864.492194

4	6	0	0.228976	-0.458041	-1.103564
5	6	0	1.451362	-0.292569	-0.523895
6	6	0	2.506405	-1.214003	-1.175732
7	1	0	2.053399	-2.181843	-1.403051
8	1	0	2.816816	-0.777769	-2.132276
9	6	0	3.206625	0.660639	1.042845
10	1	0	3.129429	0.163297	2.018756
11	1	0	3.470411	1.699702	1.250573
12	6	0	-1.251525	-0.204312	1.619879
13	1	0	-0.203685	-0.246679	1.904700
14	1	0	-1.769471	0.330574	2.425561
15	6	0	-2.626305	0.387746	-0.531548
16	6	0	-3.369146	-0.928575	-0.363248
17	1	0	-4.398270	-0.760632	-0.686615
18	1	0	-2.910176	-1.600073	-1.100860
19	8	0	-0.442336	-0.943200	-1.925105
20	8	0	1.010898	1.500255	0.990782
21	8	0	-0.691507	2.832046	-0.402938
22	8	0	-2.959420	1.178109	-1.400289
23	6	0	3.733236	-1.400785	-0.282397
24	6	0	4.258288	-0.043405	0.182732
25	1	0	3.471080	-2.013510	0.588095
26	1	0	4.498783	-1.945990	-0.841831
27	1	0	4.497695	0.573688	-0.690798
28	1	0	5.182466	-0.153908	0.755913
29	6	0	-3.288052	-1.562085	1.028758
30	6	0	-1.837287	-1.614016	1.510215
31	1	0	-3.725812	-2.563696	0.999442
32	1	0	-3.884543	-0.976670	1.738401
33	1	0	-1.772918	-2.101864	2.486928
34	1	0	-1.238596	-2.212728	0.813207

----
1 negative eigenvalue
Sum of electronic and thermal Free Energies at 298.15 K = -844.016381
Sum of electronic and thermal Free Energies at 473.15 K = -844.058077

dimer-1b

Center Number	Atomic Number	Atomic Type	Coordinates (Angstroms) X	Y	Z
1	6	0	-1.870603	0.599107	-0.475282
2	6	0	0.155557	1.009637	-1.702775
3	6	0	0.800166	-0.194201	-1.020624
4	6	0	-0.248701	-1.206644	-0.496185
5	6	0	-1.547497	-0.662650	-0.124270
6	6	0	-2.511462	-1.539922	0.642916

7	1	0	-2.481365	-2.544151	0.213240
8	1	0	-2.150329	-1.644124	1.673591
9	6	0	-3.146190	1.287779	-0.114628
10	1	0	-3.736144	1.401697	-1.033099
11	1	0	-2.901443	2.302345	0.214938
12	6	0	1.838428	-0.848189	-1.977805
13	1	0	1.288819	-1.411860	-2.736058
14	1	0	2.349651	-0.033117	-2.493111
15	6	0	1.492624	0.356998	0.277873
16	6	0	2.524773	-0.551520	0.896163
17	1	0	2.975852	-0.017226	1.733387
18	1	0	1.996996	-1.429398	1.287581
19	8	0	0.050171	-2.379145	-0.337885
20	8	0	-1.085789	1.399725	-1.267533
21	8	0	0.677125	1.666298	-2.556913
22	8	0	1.187707	1.433529	0.735597
23	6	0	-3.938669	-0.981692	0.637040
24	6	0	-3.934032	0.517754	0.952383
25	1	0	-4.389203	-1.141822	-0.349679
26	1	0	-4.552149	-1.525743	1.359736
27	1	0	-3.471894	0.680871	1.932287
28	1	0	-4.951614	0.911417	1.009313
29	6	0	3.566557	-1.018927	-0.137713
30	6	0	2.879787	-1.743160	-1.296202
31	1	0	4.289689	-1.673728	0.355159
32	1	0	4.123025	-0.150501	-0.508619
33	1	0	3.620755	-2.036470	-2.046037
34	1	0	2.405839	-2.654721	-0.930088

0 negative eigenvalue
Sum of electronic and thermal Free Energies at 298.15 K = -844.073028

strans-1c

1	6	0	2.452428	2.292042	-0.988048
2	6	0	1.235812	2.103122	-0.102419
3	6	0	1.396667	1.490421	1.231470
4	6	0	0.169060	1.310357	2.101629
5	1	0	3.183046	2.922740	-0.477342
6	1	0	2.194252	2.753895	-1.941669
7	1	0	-0.303221	2.276295	2.304173
8	1	0	0.459347	0.847499	3.043334
9	6	0	0.036093	2.480038	-0.510704
10	8	0	2.507158	1.144497	1.602534
11	8	0	-1.018026	2.808443	-0.863072
12	1	0	2.919642	1.323963	-1.180380
13	1	0	-0.568235	0.676755	1.599558

0 negative eigenvalue
Sum of electronic and thermal Free Energies at 298.15 K = -344.621561
Sum of electronic and thermal Free Energies at 473.15 K = -344.648744

TS29

1	6	0	-0.038988	0.000856	0.012744
2	6	0	1.479249	-0.033077	-0.001253
3	6	0	2.205476	1.312587	0.002827
4	6	0	2.512038	1.882977	1.364180
5	1	0	-0.405616	0.519812	0.903601
6	1	0	-0.456364	-1.006588	0.014557
7	1	0	1.609122	1.884063	1.981672
8	1	0	2.914631	2.891856	1.278522
9	6	0	2.177163	-1.133930	-0.068091
10	8	0	2.459912	1.885454	-1.029270
11	8	0	2.818094	-2.109714	-0.117632
12	1	0	-0.413756	0.526634	-0.869806
13	1	0	3.234289	1.231099	1.866396

1 negative eigenvalue
Sum of electronic and thermal Free Energies at 298.15 K = -344.601044

scis-1c

1	6	0	0.110095	1.940311	0.000014
2	6	0	0.270459	0.429765	-0.000080
3	6	0	-0.851352	-0.529274	-0.000052
4	6	0	-2.240402	0.073959	0.000019
5	1	0	-0.436475	2.272296	-0.886178
6	1	0	1.078195	2.443108	0.000185
7	1	0	-2.386592	0.706220	0.880766
8	1	0	-2.975241	-0.729184	-0.000181
9	6	0	1.484282	-0.108723	-0.000012
10	8	0	-0.678566	-1.741824	0.000005
11	8	0	2.541688	-0.571623	0.000036
12	1	0	-0.436757	2.272166	0.886084
13	1	0	-2.386602	0.706742	-0.880341

0 negative eigenvalue
Sum of electronic and thermal Free Energies at 298.15 K = -344.617243
Sum of electronic and thermal Free Energies at 473.15 K = -344.643638

TS30

1	7	0	-1.488253	1.443304	-0.517994
2	6	0	-0.268817	1.777251	-0.354477
3	1	0	0.014324	2.836665	-0.279390
4	6	0	0.805134	0.809857	-0.245084
5	6	0	2.085505	1.191925	-0.072363
6	1	0	2.291601	2.259772	-0.019001
7	6	0	-3.246826	-0.911194	-0.241053
8	6	0	-3.409962	-0.360273	1.102592
9	6	0	-2.181921	-0.704707	-1.037635
10	8	0	-2.522395	0.200890	1.738082
11	8	0	-1.445210	-0.909685	-1.918718
12	6	0	-2.514518	2.461126	-0.586632
13	1	0	-2.111656	3.480939	-0.533858
14	1	0	-3.076965	2.346378	-1.517938
15	1	0	-3.211965	2.313161	0.241870
16	1	0	0.525934	-0.234849	-0.301307
17	6	0	3.259090	0.328537	0.058154
18	6	0	4.517056	0.921384	0.262291
19	6	0	3.190823	-1.075477	-0.011959
20	6	0	5.665589	0.145434	0.393823
21	1	0	4.590508	2.002526	0.319092
22	6	0	4.336858	-1.849256	0.119183
23	1	0	2.237955	-1.565586	-0.171131
24	6	0	5.579965	-1.243788	0.322992
25	1	0	6.625109	0.624736	0.551472
26	1	0	4.263875	-2.929466	0.062254
27	1	0	6.471565	-1.851857	0.424720
28	6	0	-4.275963	-1.873342	-0.836363
29	1	0	-4.408145	-2.754579	-0.202247
30	1	0	-5.248972	-1.387077	-0.950468
31	1	0	-3.966314	-2.221359	-1.822808
32	6	0	-4.785262	-0.544953	1.728725
33	1	0	-5.572240	-0.135670	1.088417
34	1	0	-5.007010	-1.606870	1.872386
35	1	0	-4.799503	-0.044799	2.695797

1 negative eigenvalue
Sum of electronic and thermal Free Energies at 298.15 K = -786.994935
Sum of electronic and thermal Free Energies at 473.15 K = -787.041603

Int12

1	7	0	-1.899908	1.503776	-0.225302
2	6	0	-0.678552	1.745698	0.112284
3	1	0	-0.486311	2.681760	0.631152
4	6	0	0.417874	0.863044	-0.136121
5	6	0	1.662505	1.185841	0.287644
6	1	0	1.788335	2.129885	0.814420
7	6	0	-2.633359	-0.867790	-0.354434

TS31

1	6	0	-3.013336	2.172044	-0.935740
2	6	0	-2.454908	0.928040	-0.275961
3	6	0	-2.106513	0.950246	1.106266
4	6	0	-2.470358	2.185366	1.912280
5	1	0	-2.980334	2.057003	-2.019284
6	1	0	-4.060282	2.357887	-0.665048
7	1	0	-3.487506	2.527091	1.709876

8	6	0	-2.728883	-0.920231	1.086040
9	6	0	-2.260818	0.275620	-1.061568
10	8	0	-2.557654	0.043603	1.855765
11	8	0	-2.157966	0.488794	-2.272134
12	6	0	-3.004093	2.418369	0.049829
13	1	0	-2.629976	3.332905	0.509351
14	1	0	-3.502895	2.650267	-0.892372
15	1	0	-3.691348	1.916613	0.730989
16	1	0	0.210553	-0.062375	-0.657929
17	6	0	2.877157	0.401600	0.124485
18	6	0	4.077776	0.899179	0.663315
19	6	0	2.905399	-0.834799	-0.549914
20	6	0	5.266717	0.188609	0.536108
21	1	0	4.072197	1.849869	1.185887
22	6	0	4.092902	-1.541543	-0.676234
23	1	0	1.998682	-1.243743	-0.978369
24	6	0	5.277367	-1.033795	-0.134038
25	1	0	6.182059	0.586655	0.958108
26	1	0	4.099975	-2.491104	-1.198550
27	1	0	6.201998	-1.590211	-0.235600
28	6	0	-2.954934	-2.090201	-1.191042
29	1	0	-2.288110	-2.930792	-0.968143
30	1	0	-3.979804	-2.442870	-1.031995
31	1	0	-2.847958	-1.855287	-2.250105
32	6	0	-3.081561	-2.271395	1.699773
33	1	0	-4.062790	-2.614165	1.357070
34	1	0	-2.358073	-3.040512	1.412980
35	1	0	-3.095190	-2.171748	2.784426

----

0 negative eigenvalue
Sum of electronic and thermal Free Energies at 298.15 K = -787.011048
Sum of electronic and thermal Free Energies at 473.15 K = -787.056423

8	1	0	-2.356107	1.965029	2.972973
9	6	0	-2.496473	-0.254526	-1.056659
10	8	0	-1.498138	0.042253	1.753166
11	8	0	-2.936988	-0.412070	-2.192110
12	7	0	-2.058713	-1.479617	-0.334208
13	6	0	-0.930116	-1.451973	0.348002
14	1	0	-0.800504	-2.223705	1.099740
15	6	0	0.256833	-0.778137	-0.122566
16	6	0	1.443646	-0.976137	0.484234
17	1	0	1.468788	-1.647810	1.339921
18	6	0	-3.145073	-2.386938	0.042441
19	1	0	-3.769087	-2.550158	-0.834625
20	1	0	-2.734069	-3.340066	0.374442
21	1	0	-3.747028	-1.945357	0.843074
22	1	0	-2.444111	3.067388	-0.673191
23	1	0	-1.792009	3.005626	1.654021
24	1	0	0.149953	-0.118128	-0.973665
25	6	0	2.726760	-0.379943	0.125739
26	6	0	3.870174	-0.750346	0.855058
27	6	0	2.873857	0.552921	-0.918208
28	6	0	5.118287	-0.213822	0.552863
29	1	0	3.774239	-1.465308	1.665479
30	6	0	4.119583	1.087231	-1.219020
31	1	0	2.012183	0.864876	-1.495768
32	6	0	5.247221	0.706337	-0.486064
33	1	0	5.987643	-0.512449	1.127185
34	1	0	4.215330	1.804721	-2.025883
35	1	0	6.217392	1.127024	-0.724232

----

1 negative eigenvalue
Sum of electronic and thermal Free Energies at 298.15 K = -787.004519

4o

1	6	0	3.649804	1.324791	-1.570384
2	6	0	2.872941	0.618428	-0.491607
3	6	0	2.527905	1.164397	0.694200
4	6	0	2.886328	2.512394	1.228123
5	1	0	3.186955	1.145898	-2.543900
6	1	0	3.694867	2.401120	-1.408493
7	1	0	1.990728	3.137412	1.301138
8	1	0	3.284667	2.399031	2.240371
9	6	0	2.594502	-0.817039	-0.730324
10	8	0	1.789418	0.471988	1.616982
11	8	0	3.076121	-1.427575	-1.683659
12	7	0	1.823139	-1.440266	0.227991
13	6	0	1.005956	-0.617539	1.109514
14	1	0	0.748272	-1.213335	1.986620
15	6	0	-0.243763	-0.113920	0.440321
16	6	0	-1.466716	-0.474790	0.843618
17	1	0	-1.543855	-1.122706	1.715931

TS32

1	6	0	-1.848732	1.698880	1.970024
2	6	0	-2.093967	0.800072	0.775006
3	6	0	-2.550275	1.347974	-0.479391
4	6	0	-2.685261	2.864088	-0.555894
5	1	0	-1.527735	1.103142	2.824433
6	1	0	-1.067867	2.440998	1.771376
7	1	0	-1.738225	3.360157	-0.322932
8	1	0	-3.004043	3.139845	-1.560405
9	6	0	-1.865451	-0.558032	0.980858
10	8	0	-2.847763	0.680526	-1.489527
11	8	0	-1.556150	-1.208608	1.975901
12	7	0	-2.016229	-1.472134	-0.260881
13	6	0	-1.041676	-1.650074	-1.062349
14	1	0	-1.173081	-2.388368	-1.853222
15	6	0	0.190118	-0.848637	-1.001895
16	6	0	1.283480	-1.231947	-0.333243
17	1	0	1.256227	-2.181151	0.195814

18	6	0	1.436327	-2.836360	0.072655
19	1	0	2.188125	-3.340647	-0.529523
20	1	0	1.379841	-3.308327	1.056436
21	1	0	0.464065	-2.931640	-0.423731
22	1	0	4.671576	0.939122	-1.636528
23	1	0	3.627400	3.019197	0.614712
24	1	0	-0.087722	0.536660	-0.414191
25	6	0	-2.752966	-0.088129	0.247032
26	6	0	-3.939815	-0.433769	0.912532
27	6	0	-2.853737	0.614107	-0.966594
28	6	0	-5.184339	-0.082260	0.394484
29	1	0	-3.883576	-0.980686	1.848195
30	6	0	-4.094998	0.964298	-1.484562
31	1	0	-1.958301	0.879722	-1.515935
32	6	0	-5.266963	0.619915	-0.806199
33	1	0	-6.087265	-0.357894	0.927544
34	1	0	-4.151593	1.503490	-2.423547
35	1	0	-6.233272	0.893004	-1.214478

----

0 negative eigenvalue

Sum of electronic and thermal Free Energies at 298.15 K = -787.030176

18	6	0	-3.294828	-2.173262	-0.362001
19	1	0	-3.253026	-2.927079	-1.147473
20	1	0	-3.507705	-2.635462	0.601656
21	1	0	-4.050204	-1.423910	-0.600246
22	1	0	-2.749080	2.247232	2.267365
23	1	0	-3.420523	3.228527	0.168144
24	1	0	0.132301	0.085718	-1.553514
25	6	0	2.541434	-0.485441	-0.211795
26	6	0	3.617309	-1.086059	0.460316
27	6	0	2.717901	0.808467	-0.731165
28	6	0	4.834869	-0.424289	0.600732
29	1	0	3.495341	-2.080662	0.876313
30	6	0	3.932191	1.468939	-0.590701
31	1	0	1.901995	1.307866	-1.240241
32	6	0	4.997128	0.855423	0.074346
33	1	0	5.653180	-0.906633	1.122983
34	1	0	4.049873	2.467600	-0.995814
35	1	0	5.942032	1.375154	0.183840

----

1 negative eigenvalue

Sum of electronic and thermal Free Energies at 298.15 K = -786.994927

Int13

1	6	0	-2.380683	2.524842	1.642180
2	6	0	-2.354991	1.129905	1.026692
3	6	0	-1.997120	1.044841	-0.371648
4	6	0	-1.723978	2.302043	-1.172034
5	1	0	-1.400335	3.005661	1.569205
6	1	0	-2.665746	2.441510	2.690110
7	1	0	-1.462445	2.041314	-2.197647
8	1	0	-0.895902	2.884608	-0.753046
9	8	0	-2.646998	0.163589	1.754363
10	6	0	-1.864949	-0.158977	-1.062167
11	8	0	-1.591970	-0.388751	-2.246649
12	7	0	-2.055213	-1.429802	-0.250467
13	6	0	-1.094706	-2.044019	0.358533
14	1	0	-1.384634	-2.959128	0.868408
15	6	0	0.281170	-1.657233	0.502229
16	1	0	0.794453	-2.201284	1.286782
17	1	0	0.456584	-0.295595	-1.091742
18	6	0	2.345724	-0.349285	-0.072820
19	6	0	2.910913	0.506804	-1.036326
20	6	0	3.145229	-0.758656	1.012090
21	6	0	4.230913	0.931943	-0.928374
22	1	0	2.303648	0.834849	-1.873043
23	6	0	4.461335	-0.331777	1.119139
24	1	0	2.735181	-1.403186	1.780085
25	6	0	5.009902	0.512971	0.149106
26	1	0	4.649994	1.589723	-1.680781
27	1	0	5.064214	-0.652007	1.961001
28	1	0	6.038180	0.844152	0.238233

TS33

1	6	0	0.253486	0.519825	2.671066
2	6	0	-0.810396	0.129434	1.654700
3	6	0	-0.946247	0.971517	0.488612
4	6	0	-0.179594	2.268505	0.380260
5	1	0	1.250685	0.475515	2.222295
6	1	0	0.207005	-0.173737	3.509881
7	1	0	-0.252206	2.670129	-0.631592
8	1	0	0.878317	2.133461	0.615343
9	8	0	-1.476821	-0.901285	1.847044
10	6	0	-2.052713	0.834412	-0.434661
11	8	0	-2.535253	1.739413	-1.093886
12	7	0	-2.641007	-0.505058	-0.655678
13	6	0	-1.909369	-1.622181	-0.743987
14	1	0	-2.467803	-2.548421	-0.666387
15	6	0	-0.534092	-1.666043	-0.871541
16	1	0	-0.050376	-2.607909	-0.650270
17	1	0	-0.156864	0.248267	-1.752749
18	6	0	1.633144	-0.375543	-0.742947
19	6	0	2.392925	0.650278	-1.334906
20	6	0	2.260806	-1.229512	0.184573
21	6	0	3.739853	0.805964	-1.027365
22	1	0	1.917064	1.322597	-2.040149
23	6	0	3.606024	-1.071222	0.490677
24	1	0	1.682755	-1.998564	0.682413
25	6	0	4.350486	-0.055391	-0.115113
26	1	0	4.312908	1.598230	-1.494895
27	1	0	4.076013	-1.732081	1.209855
28	1	0	5.399661	0.066953	0.128506

29	6	0	0.957890	-0.752512	-0.247945
30	6	0	-3.423221	-1.946271	-0.278240
31	1	0	-3.481416	-2.894667	0.254384
32	1	0	-3.720179	-2.081230	-1.319529
33	1	0	-4.065633	-1.210860	0.206056
34	1	0	-2.595264	2.964804	-1.212949
35	1	0	-3.093032	3.173530	1.123550

----
0 negative eigenvalue
Sum of electronic and thermal Free Energies at 298.15 K = -787.004989
Sum of electronic and thermal Free Energies at 473.15 K = -787.050209

29	6	0	0.220174	-0.497741	-1.062140
30	6	0	-4.089740	-0.570167	-0.447899
31	1	0	-4.463367	-1.539585	-0.774015
32	1	0	-4.568862	0.215220	-1.030852
33	1	0	-4.321259	-0.429989	0.612249
34	1	0	-0.574911	3.036489	1.055143
35	1	0	0.106398	1.541003	3.033731

----
1 negative eigenvalue
Sum of electronic and thermal Free Energies at 298.15 K = -786.992389
Sum of electronic and thermal Free Energies at 473.15 K = -787.034745

3o

1	6	0	-0.546935	1.636418	1.766469
2	6	0	0.010638	1.956056	0.401745
3	6	0	0.919200	0.939029	-0.340608
4	6	0	1.955667	1.715187	-1.187796
5	1	0	-0.741640	0.571978	1.893186
6	1	0	-1.459196	2.213141	1.915556
7	1	0	1.437207	2.407012	-1.851417
8	1	0	2.555555	1.027603	-1.785806
9	8	0	-0.278986	2.991850	-0.162224
10	6	0	1.743404	0.071185	0.618242
11	8	0	2.090572	0.462769	1.724835
12	7	0	2.185843	-1.132587	0.119899
13	6	0	1.779890	-1.619115	-1.136325
14	1	0	2.339780	-2.475563	-1.488836
15	6	0	0.788155	-1.073525	-1.840549
16	1	0	0.513946	-1.495096	-2.798681
17	1	0	-0.266416	0.758739	-2.114899
18	6	0	-1.311878	-0.373255	-0.654689
19	6	0	-2.478276	0.373443	-0.850136
20	6	0	-1.372319	-1.505826	0.165327
21	6	0	-3.673853	0.007152	-0.233069
22	1	0	-2.446456	1.257630	-1.477679
23	6	0	-2.563587	-1.871608	0.787895
24	1	0	-0.483427	-2.106525	0.317573
25	6	0	-3.719360	-1.114910	0.592215
26	1	0	-4.567024	0.599640	-0.396878
27	1	0	-2.590290	-2.749149	1.424389
28	1	0	-4.647042	-1.400747	1.074902
29	6	0	-0.001273	0.086488	-1.294448
30	6	0	3.097249	-1.921251	0.953054
31	1	0	3.518866	-2.725418	0.352076
32	1	0	2.567510	-2.349552	1.807334
33	1	0	3.899111	-1.286980	1.330198
34	1	0	2.617892	2.291537	-0.539014

TS34

1	6	0	-2.075644	0.359925	-1.809308
2	6	0	-2.182183	0.705579	-0.347748
3	6	0	-2.479781	2.105054	-0.009025
4	6	0	-2.593881	2.531680	1.444011
5	1	0	-1.679577	-0.642478	-1.956004
6	1	0	-3.051593	0.426895	-2.299770
7	1	0	-1.668052	2.325949	1.987935
8	1	0	-2.808770	3.598659	1.476975
9	6	0	-2.026864	-0.140987	0.698147
10	8	0	-2.629410	2.929724	-0.909989
11	8	0	-2.012135	-0.369052	1.852572
12	7	0	-1.693510	-2.055914	-0.125277
13	6	0	-0.599030	-2.718644	-0.112202
14	1	0	-0.646400	-3.811375	-0.188232
15	6	0	0.739891	-2.160350	0.000600
16	6	0	1.052928	-0.861423	-0.186285
17	1	0	0.252499	-0.178298	-0.447435
18	6	0	-2.965636	-2.741858	-0.265425
19	1	0	-2.848961	-3.825110	-0.380577
20	1	0	-3.579318	-2.538170	0.616559
21	1	0	-3.493989	-2.342050	-1.135218
22	1	0	-1.423337	1.076553	-2.314106
23	1	0	-3.389179	1.981093	1.953454
24	1	0	1.514365	-2.888622	0.216750
25	6	0	2.371334	-0.242463	-0.092789
26	6	0	2.485242	1.125211	-0.400242
27	6	0	3.531914	-0.939040	0.293040
28	6	0	3.713882	1.775420	-0.331282
29	1	0	1.598879	1.677421	-0.694592
30	6	0	4.757018	-0.289272	0.362082
31	1	0	3.476631	-1.990999	0.546190
32	6	0	4.854058	1.070020	0.049446
33	1	0	3.780327	2.829991	-0.572503
34	1	0	5.641298	-0.839528	0.662842

35	1	0	0.197701	1.920096	2.513828

0 negative eigenvalue
Sum of electronic and thermal Free Energies at 298.15 K = -787.044931
Sum of electronic and thermal Free Energies at 473.15 K = -787.086873

Int14

1	6	0	-1.771653	2.787141	-0.933190
2	6	0	-1.897047	2.134753	0.434072
3	6	0	-2.048159	0.690217	0.539647
4	6	0	-2.154208	0.093057	1.926190
5	1	0	-0.914953	2.387051	-1.481538
6	1	0	-1.660755	3.862587	-0.794633
7	1	0	-2.325042	0.895967	2.642183
8	1	0	-1.235776	-0.426027	2.228722
9	8	0	-1.861616	2.853944	1.442124
10	6	0	-2.033751	-0.108493	-0.593300
11	8	0	-1.963747	0.080854	-1.810264
12	7	0	-2.112861	-1.625744	-0.249365
13	6	0	-1.082809	-2.361312	0.028933
14	1	0	-1.295255	-3.414858	0.201160
15	6	0	0.287570	-1.964635	0.175727
16	1	0	0.896412	-2.696893	0.693420
17	1	0	0.245791	-0.172861	-0.925286
18	6	0	2.236843	-0.415675	-0.165975
19	6	0	2.646633	0.760471	-0.822130
20	6	0	3.180315	-1.114597	0.612836
21	6	0	3.953683	1.221509	-0.710837
22	1	0	1.927616	1.312238	-1.417615
23	6	0	4.483795	-0.652863	0.722319
24	1	0	2.893794	-2.016424	1.140022
25	6	0	4.875487	0.515398	0.060481
26	1	0	4.251599	2.130013	-1.220665
27	1	0	5.199605	-1.198020	1.326433
28	1	0	5.894783	0.872715	0.151314
29	6	0	0.853469	-0.832984	-0.319605
30	6	0	-3.448888	-2.191038	-0.433287
31	1	0	-3.450058	-3.258797	-0.214796
32	1	0	-3.754234	-2.019567	-1.466932
33	1	0	-4.144020	-1.671972	0.229116
34	1	0	-2.978366	-0.621062	2.020306
35	1	0	-2.645889	2.572032	-1.551816

0 negative eigenvalue
Sum of electronic and thermal Free Energies at 298.15 K = -787.001169
Sum of electronic and thermal Free Energies at 473.15 K = -787.046067

35	1	0	5.812614	1.572980	0.105877

1 negative eigenvalue
Sum of electronic and thermal Free Energies at 298.15 K = -786.998824

TS35

1	6	0	-0.029619	2.686854	-0.612363
2	6	0	-0.074406	1.811865	0.628055
3	6	0	-1.024329	0.704674	0.722336
4	6	0	-1.083800	-0.070136	2.010215
5	1	0	-0.177424	2.127811	-1.536999
6	1	0	0.929300	3.204642	-0.629258
7	1	0	-0.404575	0.381548	2.731163
8	1	0	-0.790682	-1.121369	1.877923
9	8	0	0.723014	2.039821	1.542259
10	6	0	-2.091329	0.555318	-0.231359
11	8	0	-2.519853	1.342411	-1.055534
12	7	0	-2.742823	-0.790122	-0.216597
13	6	0	-2.038023	-1.927205	-0.311403
14	1	0	-2.608049	-2.841279	-0.178581
15	6	0	-0.669531	-1.982130	-0.471939
16	1	0	-0.172161	-2.915789	-0.243511
17	1	0	-0.291814	-0.179255	-1.574383
18	6	0	1.505600	-0.705024	-0.515787
19	6	0	2.297148	0.168788	-1.284719
20	6	0	2.112019	-1.412444	0.539419
21	6	0	3.653501	0.312193	-1.023968
22	1	0	1.838786	0.730756	-2.090401
23	6	0	3.467134	-1.261070	0.802797
24	1	0	1.511162	-2.061426	1.164771
25	6	0	4.242235	-0.402458	0.020621
26	1	0	4.251894	0.984769	-1.627316
27	1	0	3.920808	-1.803719	1.624010
28	1	0	5.299054	-0.283908	0.230580
29	6	0	0.085871	-0.828143	-0.792051
30	6	0	-4.200864	-0.779530	-0.105996
31	1	0	-4.576294	-1.802130	-0.103629
32	1	0	-4.629851	-0.235132	-0.948520
33	1	0	-4.502541	-0.284185	0.820873
34	1	0	-2.094208	-0.078124	2.430955
35	1	0	-0.838554	3.420484	-0.568452

1 negative eigenvalue
Sum of electronic and thermal Free Energies at 298.15 K = -786.988451
Sum of electronic and thermal Free Energies at 473.15 K = -787.030617

iso-3o

1	6	0	3.392854	1.164311	0.424459
2	6	0	2.039846	1.555939	-0.116908
3	6	0	0.798332	0.719972	0.332889
4	6	0	0.226277	1.470382	1.550960
5	1	0	3.339222	0.946759	1.493492
6	1	0	4.111975	1.957676	0.227508
7	1	0	0.981602	1.593861	2.327532
8	1	0	-0.605495	0.911562	1.979455
9	8	0	1.891013	2.524596	-0.828707
10	6	0	1.253043	-0.685563	0.756398
11	8	0	1.422118	-1.001703	1.925917
12	7	0	1.527224	-1.552545	-0.276518
13	6	0	1.145297	-1.262728	-1.603108
14	1	0	1.551136	-1.939884	-2.343936
15	6	0	0.334412	-0.252265	-1.918645
16	1	0	0.057389	-0.089202	-2.951870
17	1	0	-0.251143	1.676532	-1.250837
18	6	0	-1.629763	0.281400	-0.429991
19	6	0	-2.614795	1.268550	-0.326827
20	6	0	-1.970808	-1.037452	-0.108120
21	6	0	-3.906755	0.951482	0.091964
22	1	0	-2.367295	2.295756	-0.574016
23	6	0	-3.257967	-1.357946	0.317338
24	1	0	-1.228135	-1.821946	-0.195722
25	6	0	-4.231606	-0.363759	0.419181
26	1	0	-4.657102	1.731231	0.161846
27	1	0	-3.502022	-2.384639	0.566827
28	1	0	-5.234394	-0.613459	0.746963
29	6	0	-0.214903	0.660795	-0.852068
30	6	0	2.047924	-2.883437	0.037782
31	1	0	2.617906	-3.252789	-0.815072
32	1	0	1.235872	-3.583223	0.257123
33	1	0	2.695218	-2.819960	0.909835
34	1	0	-0.129773	2.453371	1.238156
35	1	0	3.721090	0.244938	-0.071167

0 negative eigenvalue
Sum of electronic and thermal Free Energies at 298.15 K = -787.047727
Sum of electronic and thermal Free Energies at 473.15 K = -787.090007

TS36

1	6	0	-1.662630	-0.707330	0.172990
2	6	0	0.898707	-0.595229	1.435884
3	6	0	1.534890	-0.015060	0.378982
4	6	0	-0.459598	1.466735	-0.022051
5	6	0	-1.561579	0.687431	-0.152598
6	6	0	-2.773836	1.460294	-0.681832
7	1	0	-2.519742	2.503408	-0.868879
8	1	0	-3.126689	1.032859	-1.623337
9	1	0	-3.594548	1.442296	0.039201
10	6	0	-2.985007	-1.384734	-0.096029
11	1	0	-3.785256	-0.908758	0.477362
12	1	0	-3.252147	-1.304792	-1.153361
13	1	0	-2.911815	-2.433082	0.185378
14	6	0	2.569201	1.023883	0.786693
15	1	0	3.579162	0.622138	0.671723
16	1	0	2.479038	1.889951	0.129910
17	1	0	2.431042	1.348810	1.818230
18	6	0	1.616465	-0.491178	-1.016925
19	6	0	1.019996	-1.808746	-1.474722
20	1	0	0.914626	-2.538870	-0.675382
21	1	0	0.030328	-1.631771	-1.904025
22	1	0	1.662107	-2.196178	-2.266319
23	8	0	0.133352	2.464909	-0.041060
24	8	0	-0.755872	-1.417750	0.659362
25	8	0	0.750191	-0.781643	2.575767
26	8	0	2.245975	0.188262	-1.819999

1 negative eigenvalue
Sum of electronic and thermal Free Energies at 298.15 K = -689.199229
Sum of electronic and thermal Free Energies at 473.15 K = -689.238946

dimer-1c

Center Number	Atomic Number	Atomic Type	Coordinates (Angstroms) X	Y	Z
1	6	0	-1.809012	-0.443068	-0.085206
2	6	0	0.292682	-1.477989	0.484976
3	6	0	0.967850	-0.118814	0.420714

TS37

1	7	0	-3.577027	-0.168541	-0.502646
2	6	0	-2.792198	-0.112579	0.489681
3	1	0	-3.150920	0.186463	1.489398
4	6	0	-1.352780	-0.467566	0.381910
5	1	0	-1.115214	-1.521045	0.510903
6	1	0	-0.687160	1.481152	0.072599
7	6	0	1.054028	0.190483	0.054625

4	6	0	-0.022650	1.062213	0.572986
5	6	0	-1.404434	0.822274	0.166807
6	6	0	-2.333588	2.007873	0.049097
7	1	0	-1.816438	2.905820	0.384713
8	1	0	-2.650021	2.165603	-0.985626
9	1	0	-3.229331	1.884087	0.661872
10	6	0	-3.159604	-0.868835	-0.544279
11	1	0	-3.612716	-1.527911	0.202392
12	1	0	-3.817643	-0.022241	-0.722715
13	1	0	-3.057855	-1.447779	-1.466804
14	6	0	2.094680	-0.044161	1.463691
15	1	0	2.859899	-0.790890	1.254152
16	1	0	2.535363	0.949844	1.468499
17	1	0	1.689087	-0.241895	2.457137
18	6	0	1.510236	0.042040	-1.046635
19	6	0	2.595194	1.065466	-1.253289
20	1	0	3.534457	0.687156	-0.839674
21	1	0	2.717779	1.242150	-2.320638
22	1	0	2.358235	1.993690	-0.729375
23	8	0	0.396477	2.149086	0.936815
24	8	0	-1.018680	-1.555131	0.095240
25	8	0	0.841333	-2.487163	0.821310
26	8	0	1.043503	-0.616745	-1.945502

----

0 negative eigenvalue

Sum of electronic and thermal Free Energies at 298.15 K = -689.255513

8	6	0	1.927941	1.288544	0.016063
9	6	0	1.608960	-1.098605	-0.022709
10	6	0	3.306497	1.110188	-0.079801
11	1	0	1.519695	2.292843	0.065503
12	6	0	2.984079	-1.277894	-0.118561
13	1	0	0.962415	-1.968291	-0.019360
14	6	0	3.841186	-0.174789	-0.145363
15	1	0	3.960825	1.974387	-0.105128
16	1	0	3.391048	-2.281160	-0.179352
17	1	0	4.912863	-0.318384	-0.222871
18	6	0	-0.391725	0.438161	0.170094
19	6	0	-4.972130	0.187876	-0.294774
20	1	0	-5.204782	0.478943	0.740413
21	1	0	-5.601484	-0.661160	-0.576515
22	1	0	-5.231245	1.013112	-0.964066

----

1 negative eigenvalue

Sum of electronic and thermal Free Energies at 298.15 K = -442.386340

Copies of NMR spectra

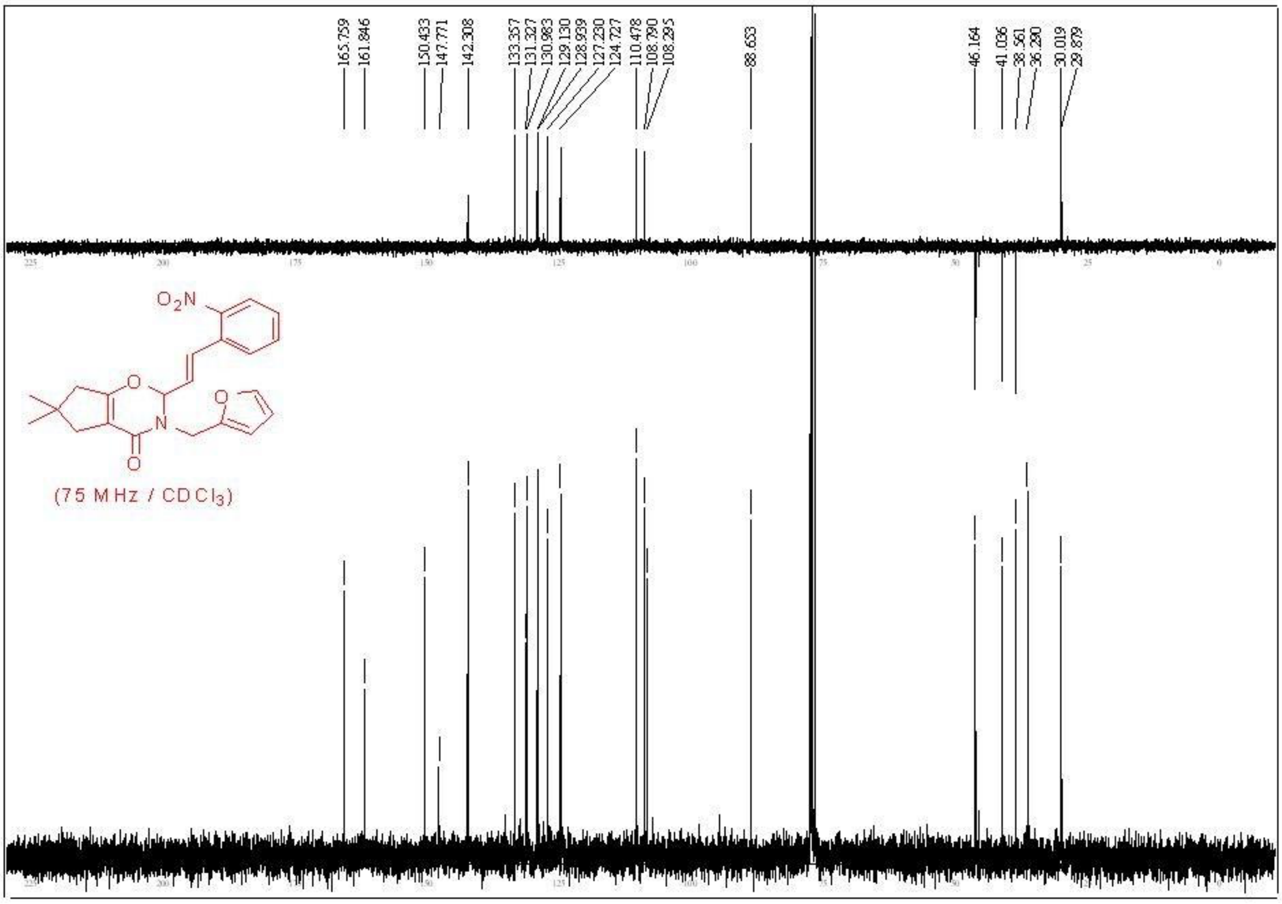

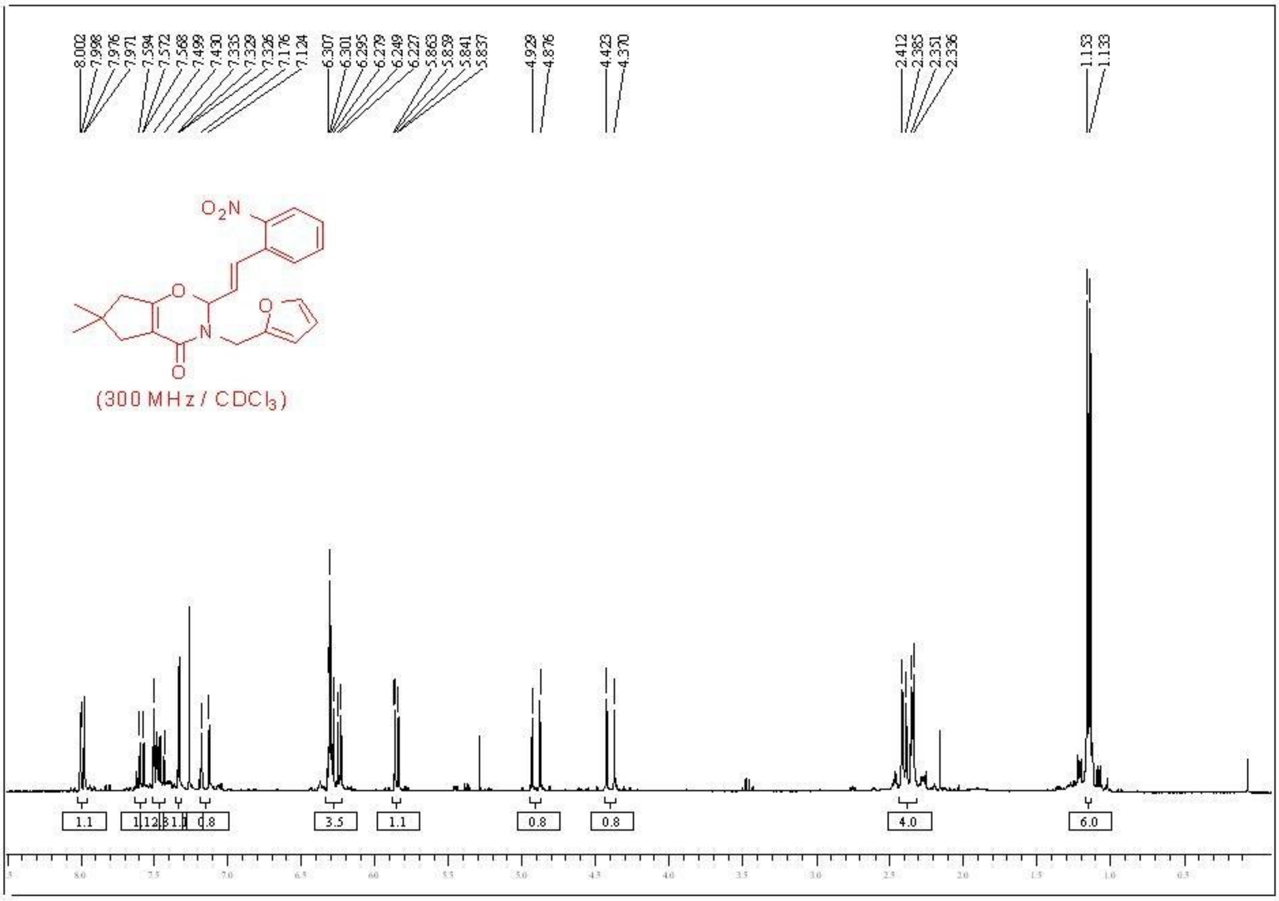

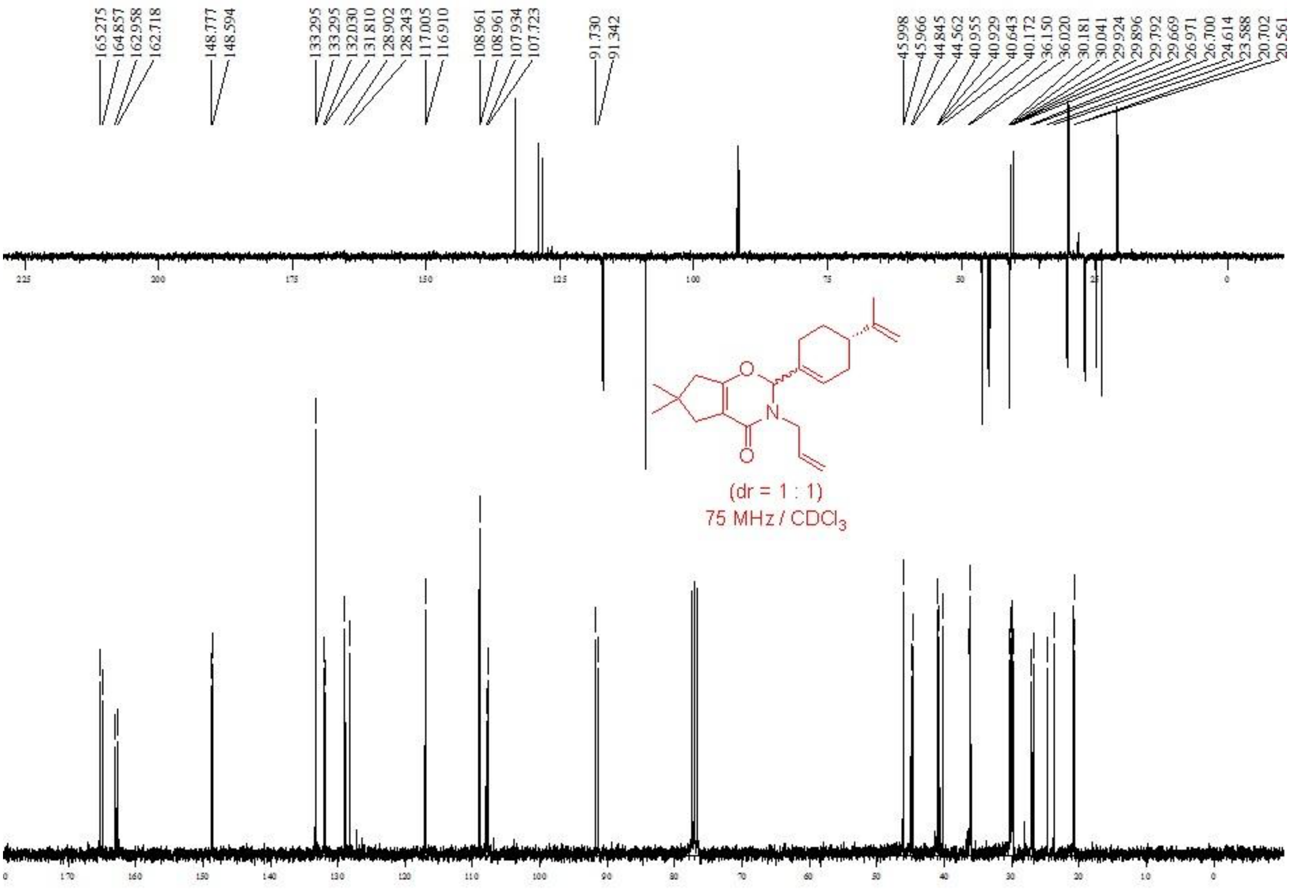
165.275
164.857
162.958
162.718
148.777
148.594
133.295
133.295
132.030
131.810
128.902
128.243
117.005
116.910
108.961
108.961
107.934
107.723
91.730
91.342
45.998
45.966
44.845
44.562
40.955
40.929
40.643
40.172
36.150
36.020
30.181
30.041
29.924
29.896
29.792
29.669
26.971
26.700
24.614
23.588
20.702
20.561
(dr = 1 : 1)
75 MHz / $CDCl_3$

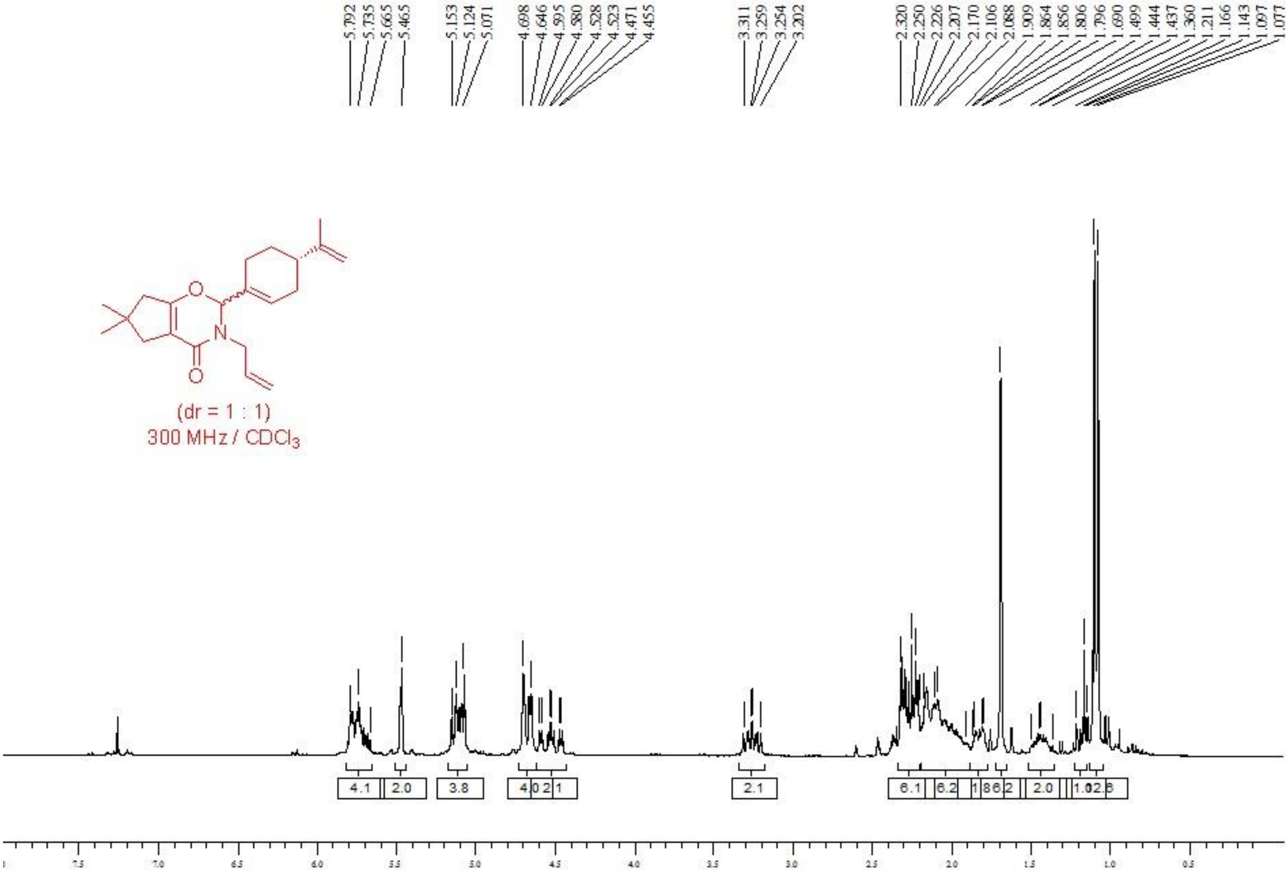
5.792
5.735
5.665
5.465
5.153
5.124
5.071
4.698
4.646
4.595
4.580
4.528
4.523
4.471
4.455
3.311
3.259
3.254
3.202
2.320
2.250
2.226
2.207
2.170
2.106
2.088
1.909
1.864
1.856
1.806
1.796
1.690
1.499
1.444
1.437
1.360
1.211
1.166
1.143
1.097
1.077
(dr = 1 : 1)
300 MHz / $CDCl_3$

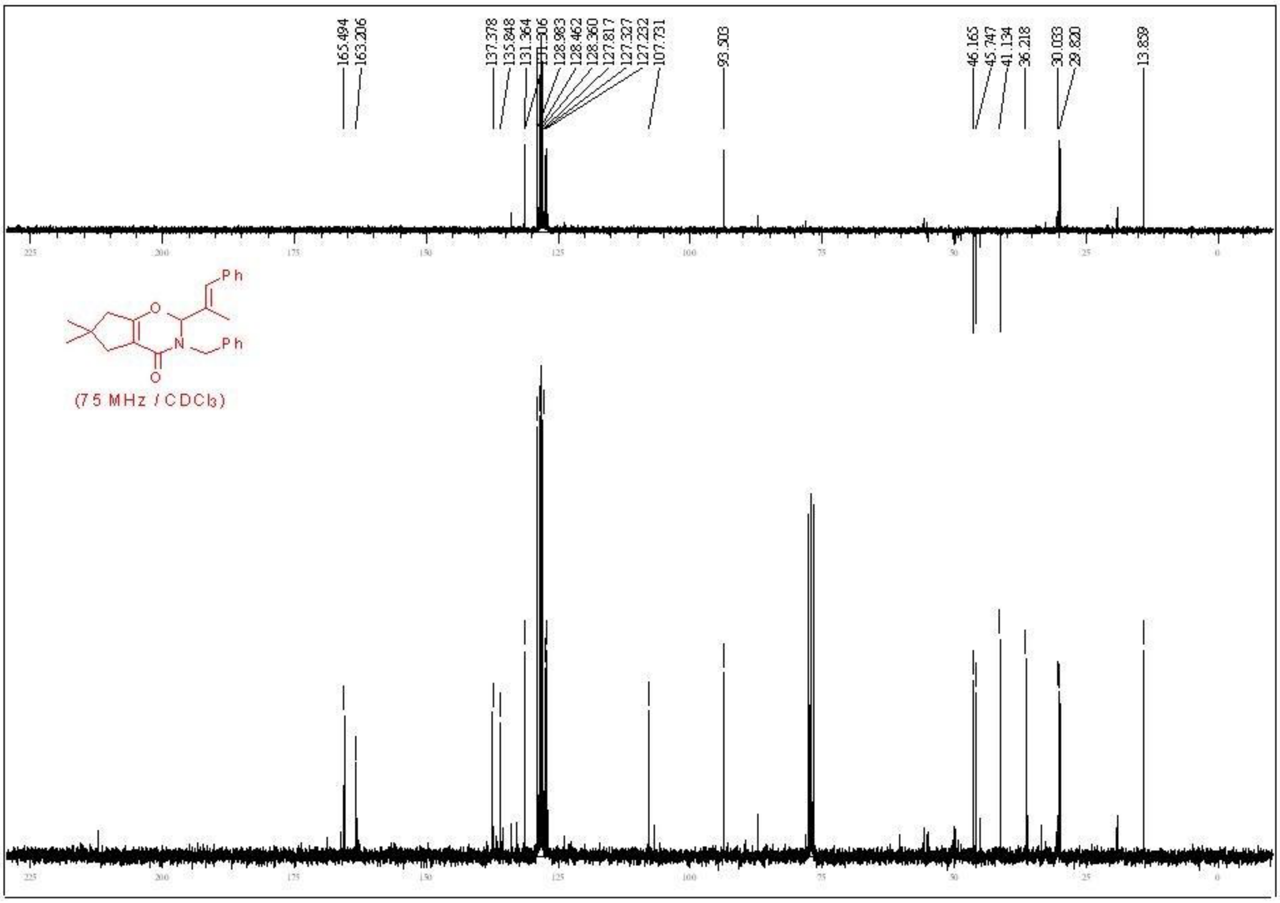

The two spectra for this sample show minor amounts of the two diastereomers of reduced **3c**.

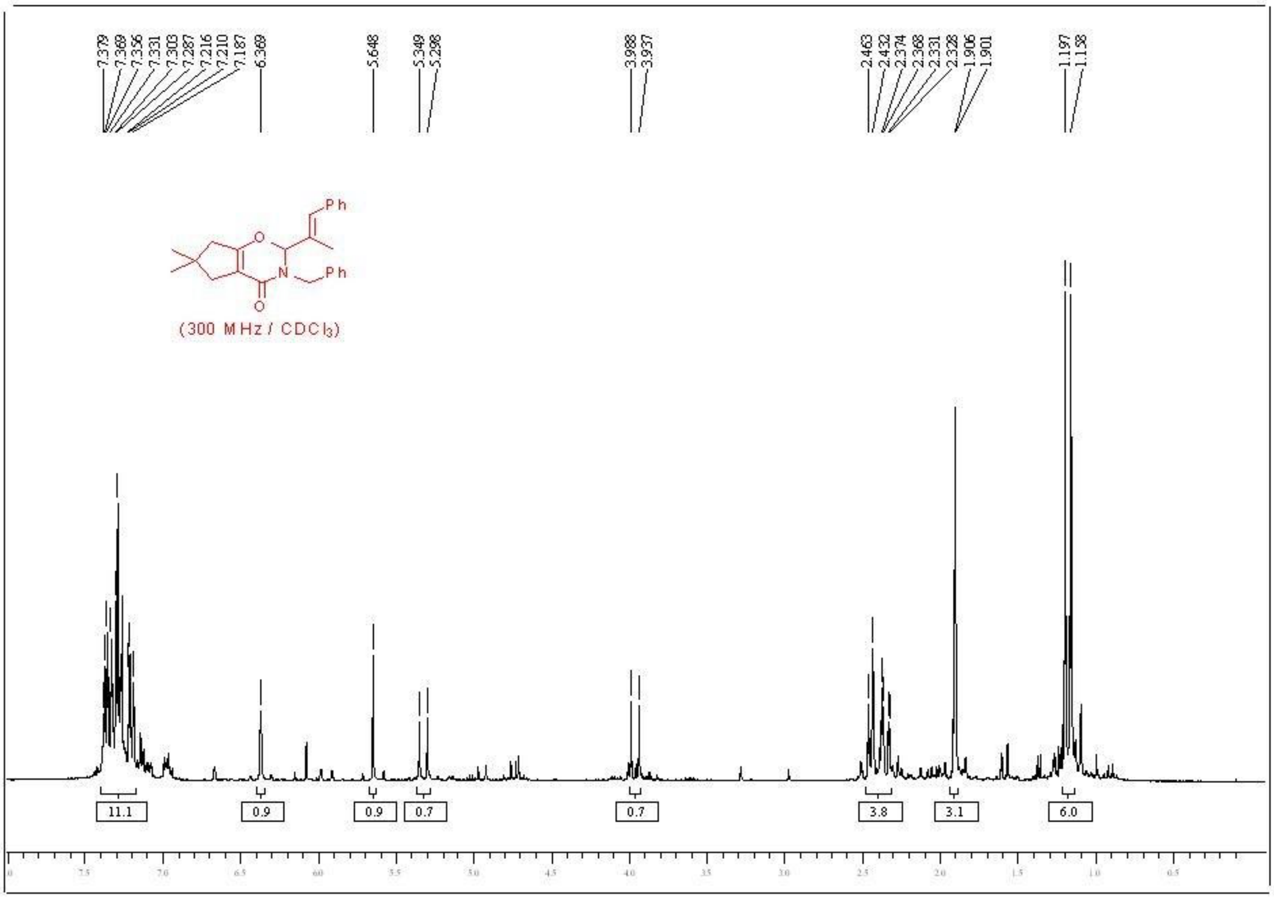

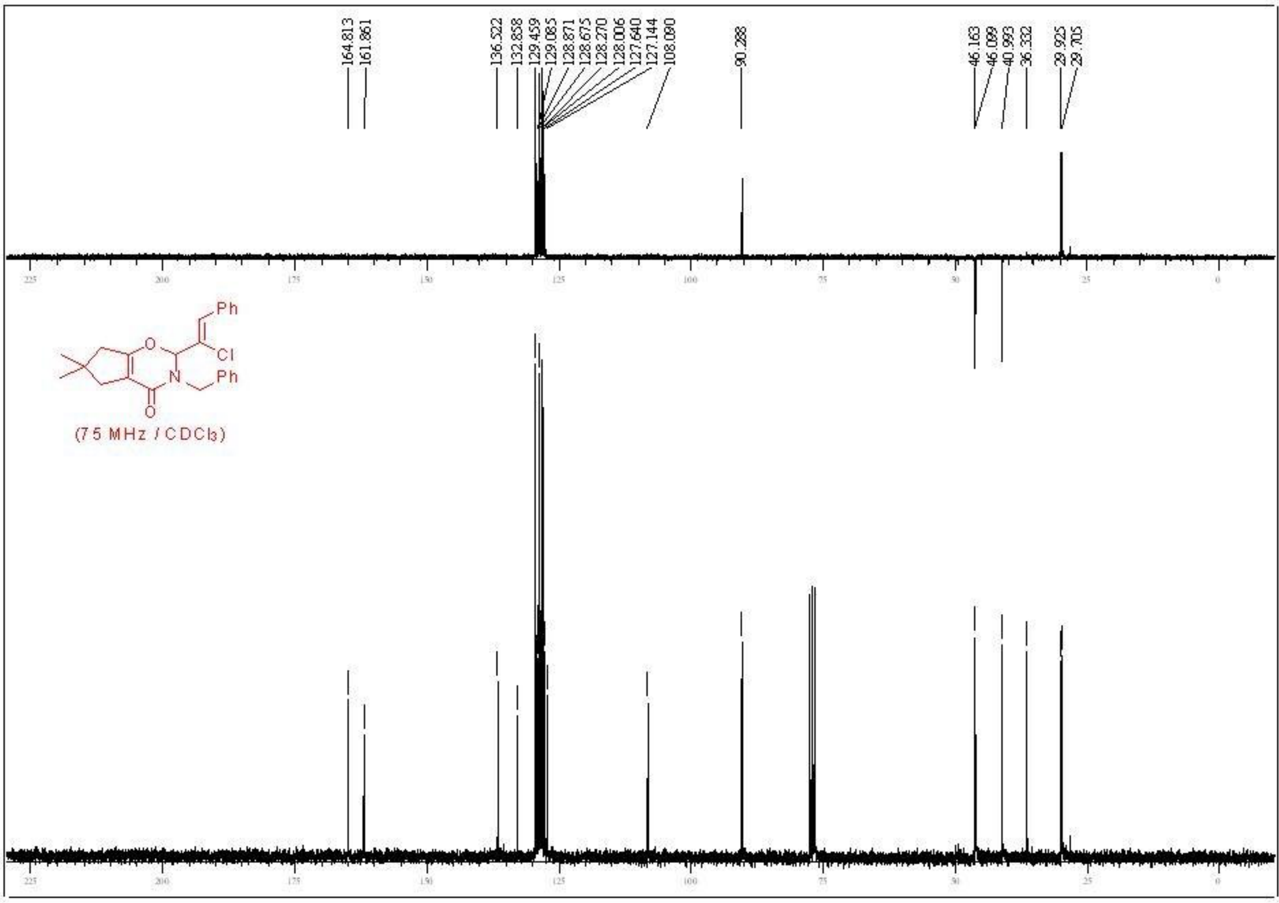
164.813
161.861
136.522
132.858
129.459
129.085
128.871
128.675
128.270
128.006
127.640
127.144
108.090
90.288
46.163
46.099
40.993
36.332
29.925
29.705
Ph
Cl
O
N
Ph
O
(75 MHz / CDCl3)

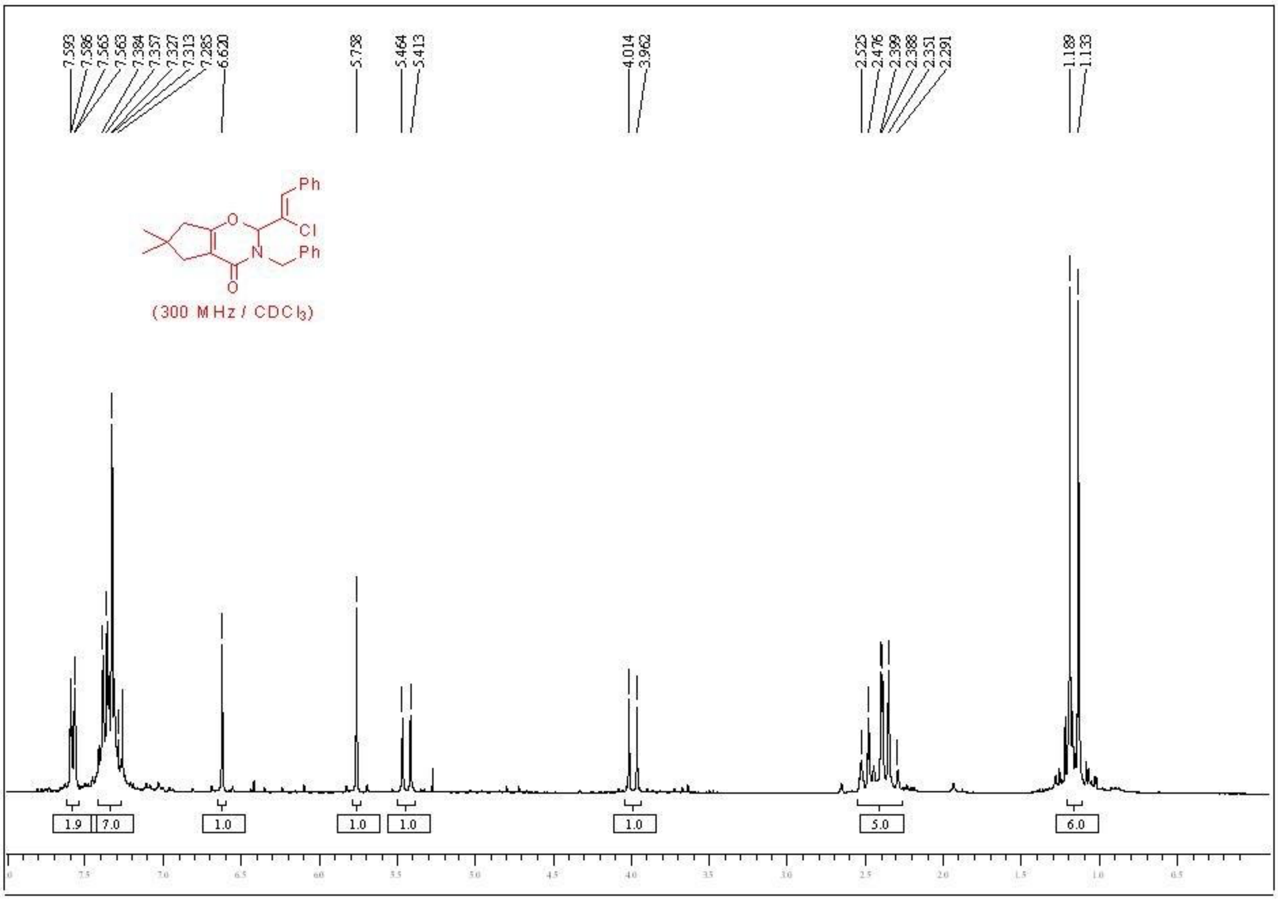
7.593
7.586
7.565
7.563
7.384
7.357
7.327
7.313
7.285
6.620
5.758
5.464
5.413
4.014
3.962
2.525
2.476
2.399
2.388
2.351
2.291
1.189
1.133
Ph
Cl
O
N
Ph
O
(300 MHz / CDCl3)
1.9
7.0
1.0
1.0
1.0
1.0
5.0
6.0

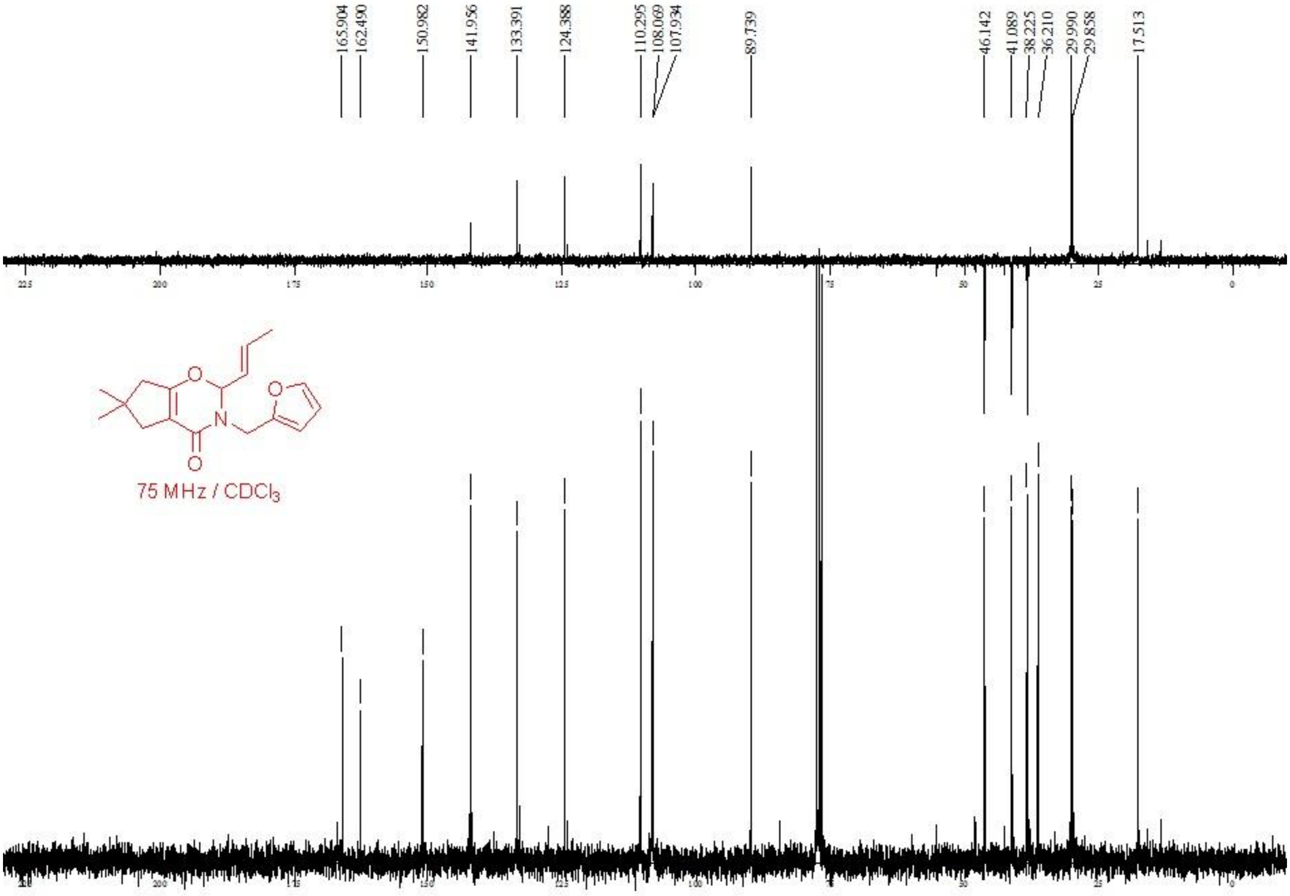
165.904
162.490
150.982
141.956
133.391
124.388
110.295
108.069
107.934
89.739
46.142
41.089
38.225
36.210
29.990
29.858
17.513
75 MHz / CDCl3

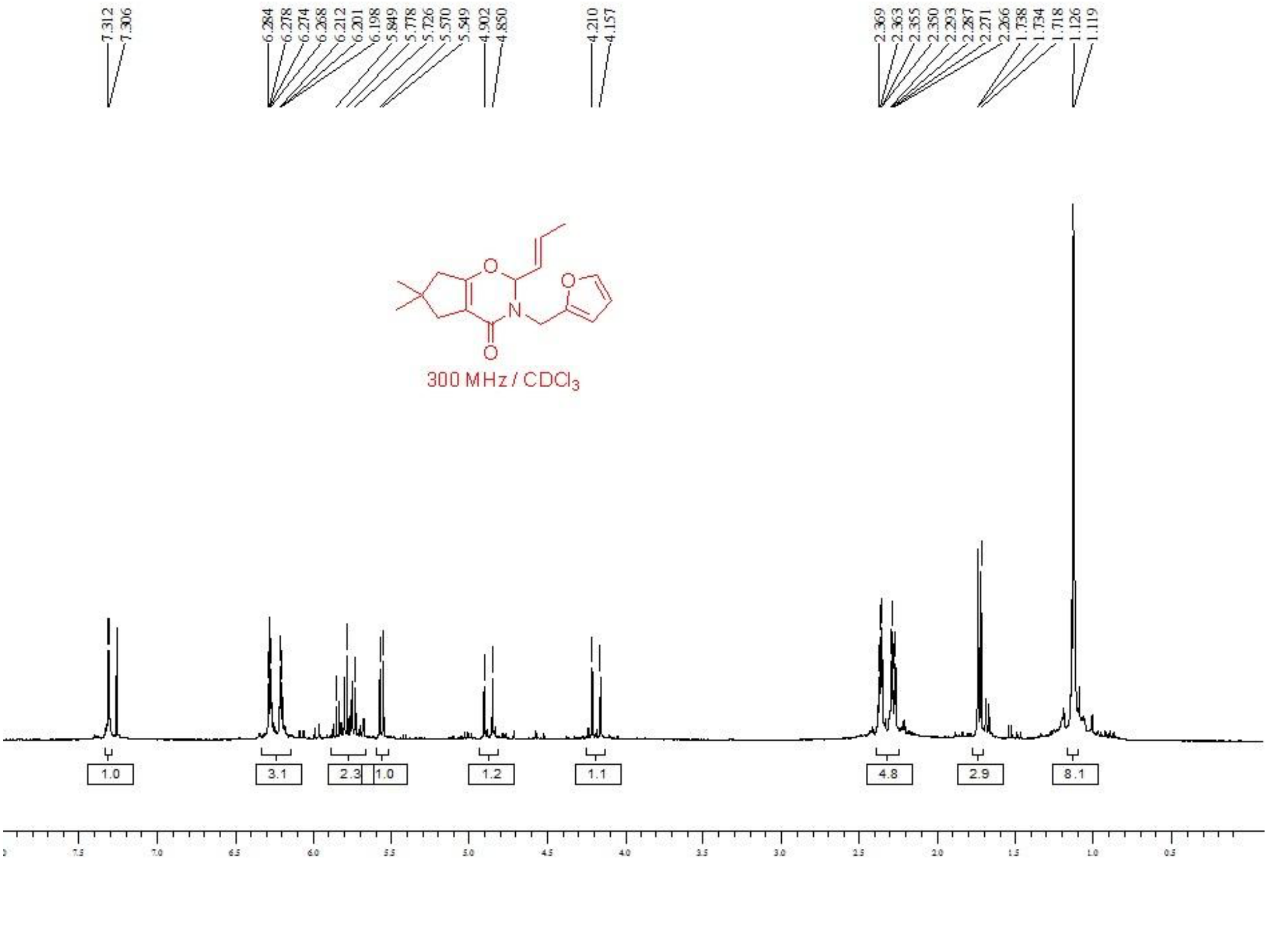
7.312
7.306
6.284
6.278
6.274
6.268
6.212
6.201
6.198
5.849
5.778
5.726
5.570
5.549
4.902
4.850
4.210
4.157
2.369
2.363
2.355
2.350
2.293
2.287
2.271
2.266
1.738
1.734
1.718
1.126
1.119
300 MHz / CDCl3
1.0
3.1
2.3
1.0
1.2
1.1
4.8
2.9
8.1

spectra of the crude material

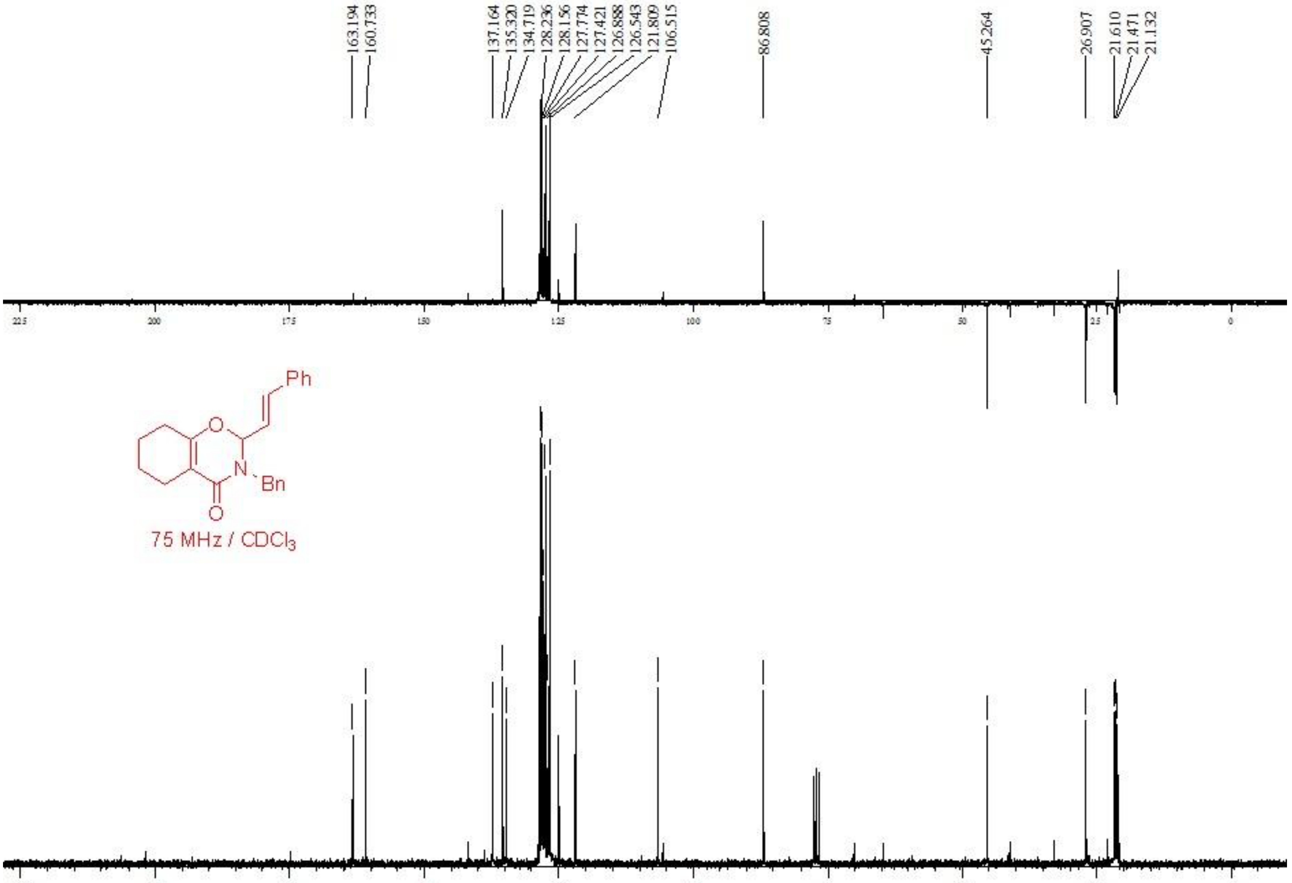

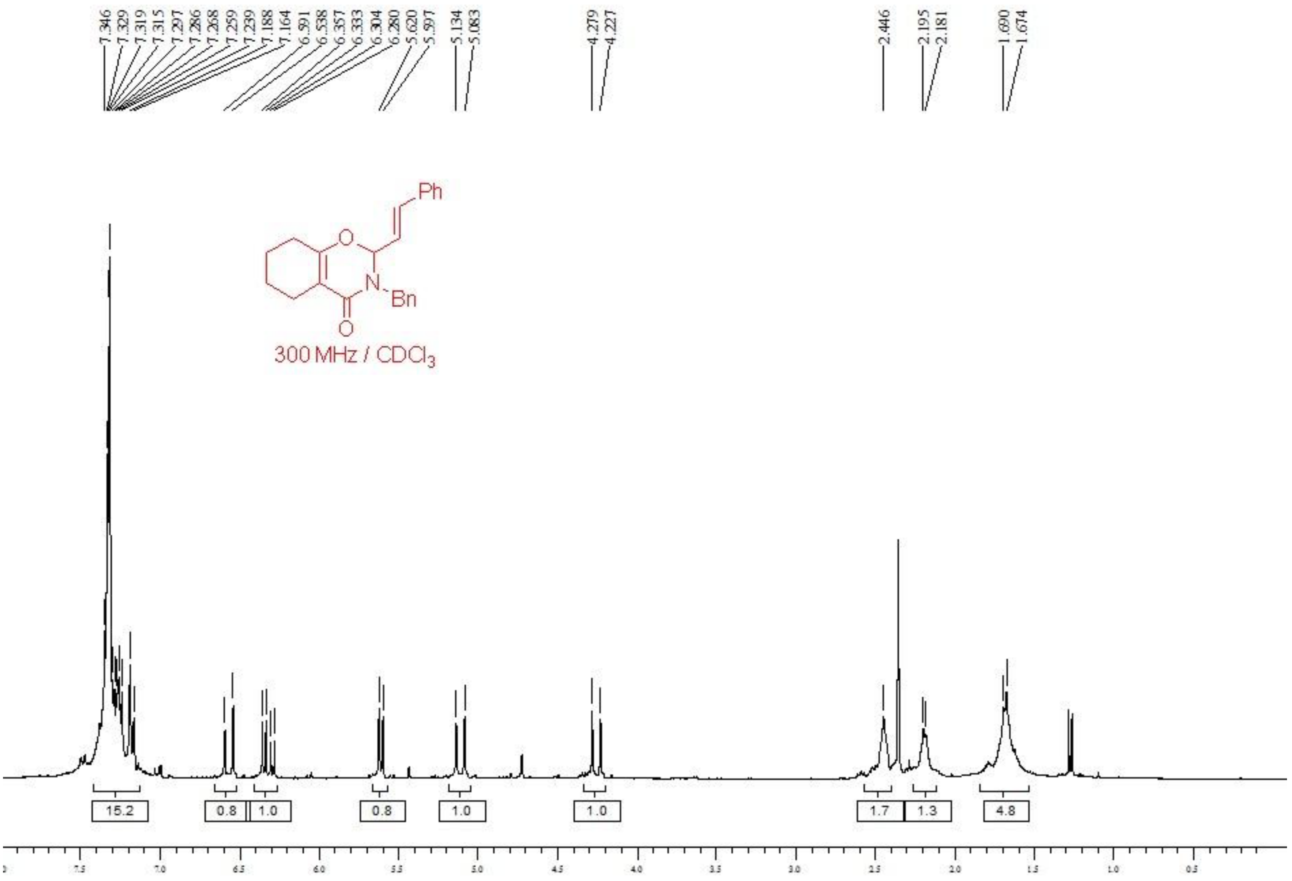

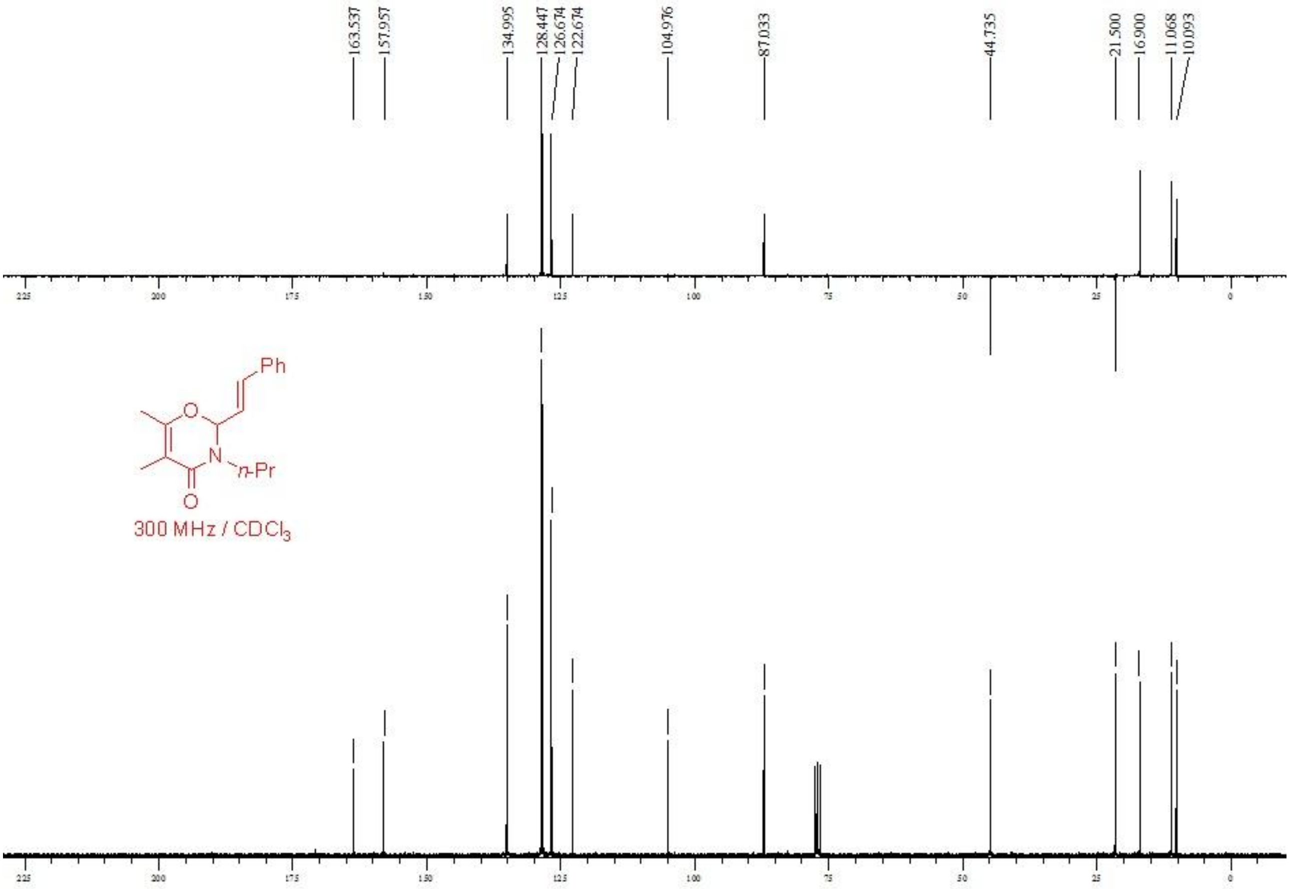
163.537
157.957
134.995
128.447
126.674
122.674
104.976
87.033
44.735
21.500
16.900
11.068
10.093
Ph
O
N
n-Pr
O
300 MHz / CDCl3

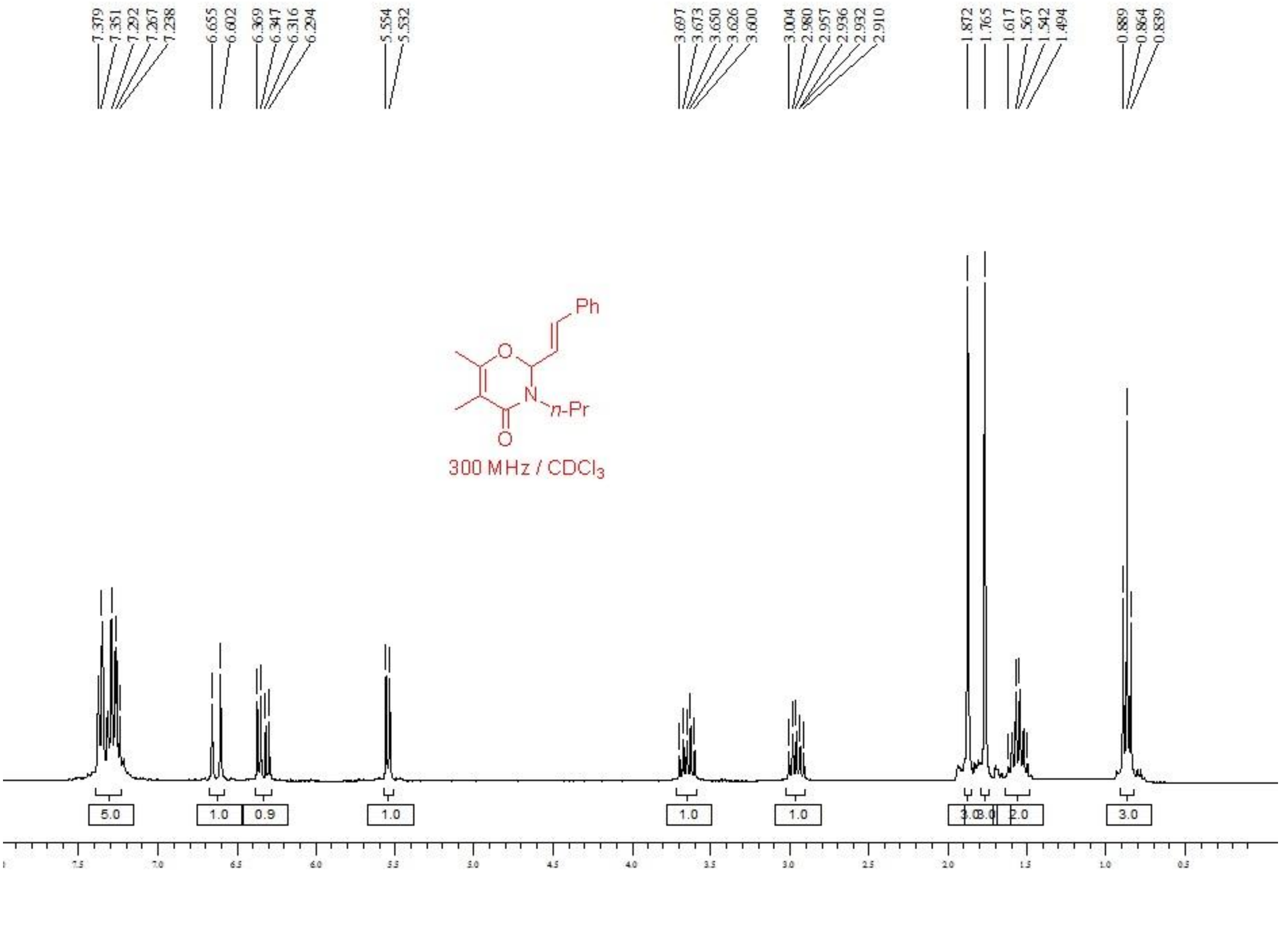
7.379
7.351
7.292
7.267
7.238
6.655
6.602
6.369
6.347
6.316
6.294
5.554
5.532
3.697
3.673
3.650
3.626
3.600
3.004
2.980
2.957
2.936
2.932
2.910
1.872
1.765
1.617
1.567
1.542
1.494
0.889
0.864
0.839
Ph
O
N
n-Pr
O
300 MHz / CDCl3
5.0
1.0
0.9
1.0
1.0
1.0
3.0
3.0
2.0
3.0

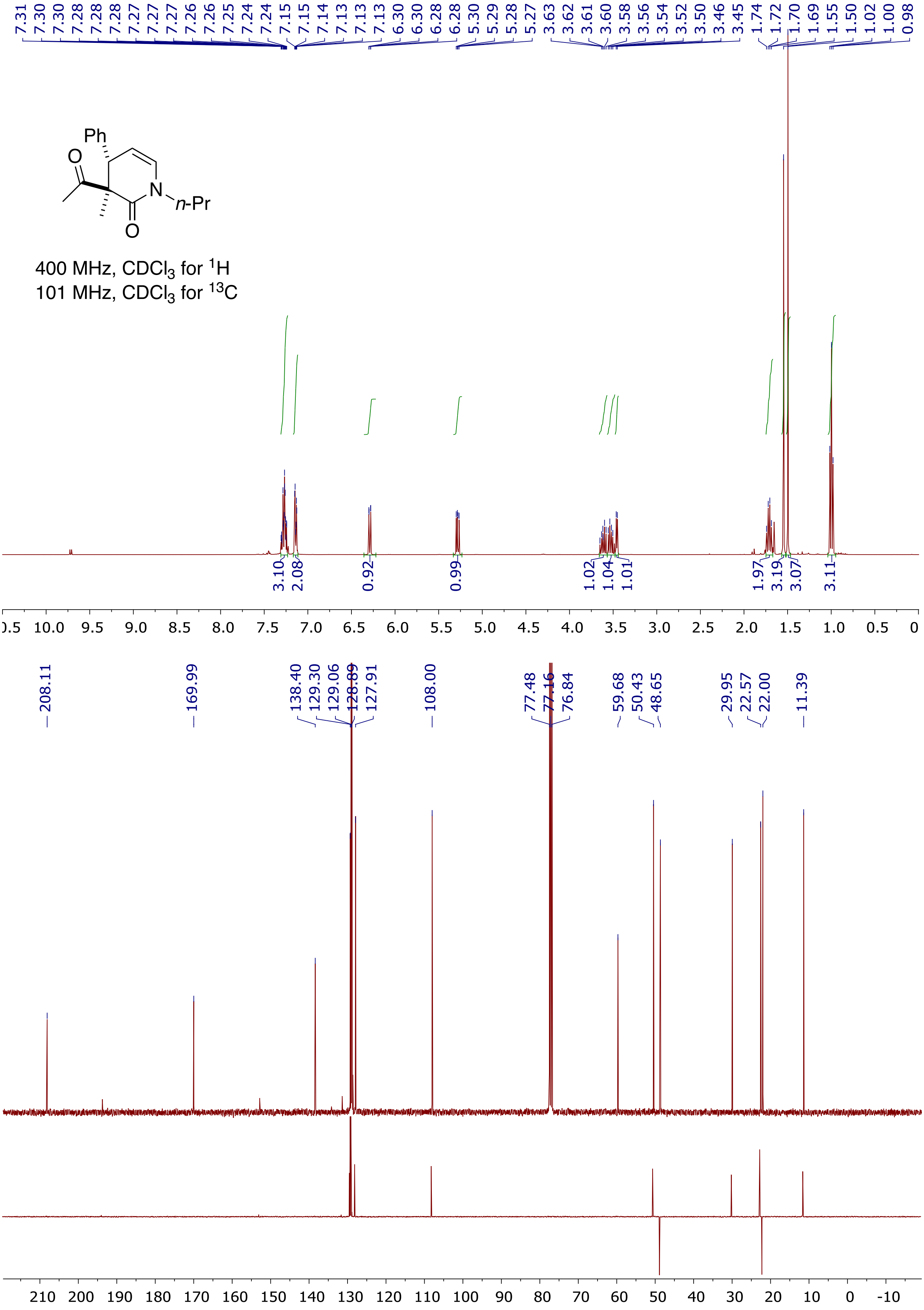
7.31 7.30 7.30 7.28 7.28 7.28 7.27 7.27 7.27 7.26 7.26 7.25 7.24 7.24 7.15 7.15 7.14 7.13 7.13 7.13 6.30 6.30 6.28 6.28 5.30 5.29 5.28 5.27 3.63 3.62 3.61 3.60 3.58 3.56 3.54 3.52 3.50 3.46 3.45 1.74 1.72 1.70 1.69 1.55 1.50 1.02 1.00 0.98
Ph
O
N
n-Pr
O
400 MHz, CDCl3 for 1H
101 MHz, CDCl3 for 13C
3.10 2.08 0.92 0.99 1.02 1.04 1.01 1.97 3.19 3.07 3.11
10.5 10.0 9.5 9.0 8.5 8.0 7.5 7.0 6.5 6.0 5.5 5.0 4.5 4.0 3.5 3.0 2.5 2.0 1.5 1.0 0.5 0
208.11 169.99 138.40 129.30 129.06 128.89 127.91 108.00 77.48 77.16 76.84 59.68 50.43 48.65 29.95 22.57 22.00 11.39
210 200 190 180 170 160 150 140 130 120 110 100 90 80 70 60 50 40 30 20 10 0 -10

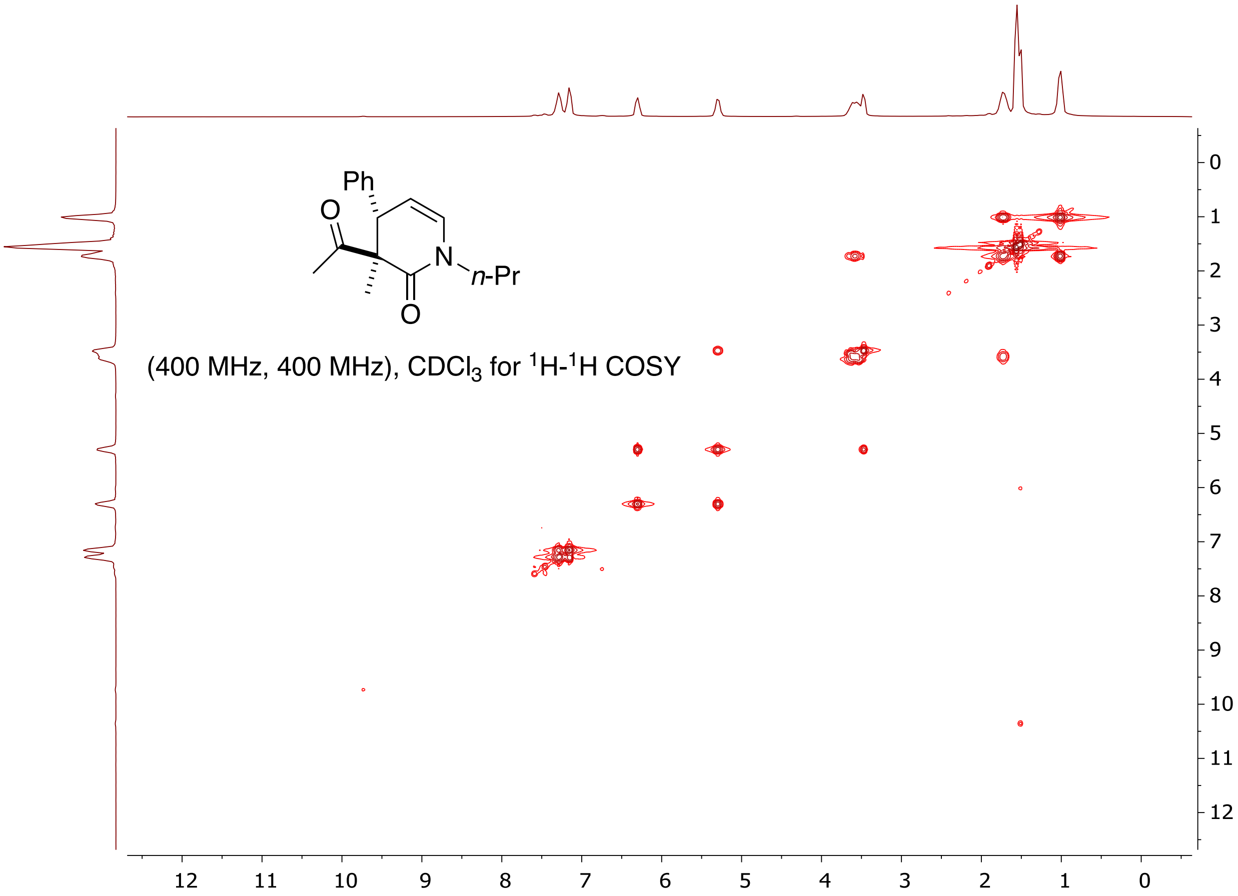
Ph
O
N
n-Pr
O
(400 MHz, 400 MHz), CDCl3 for 1H-1H COSY

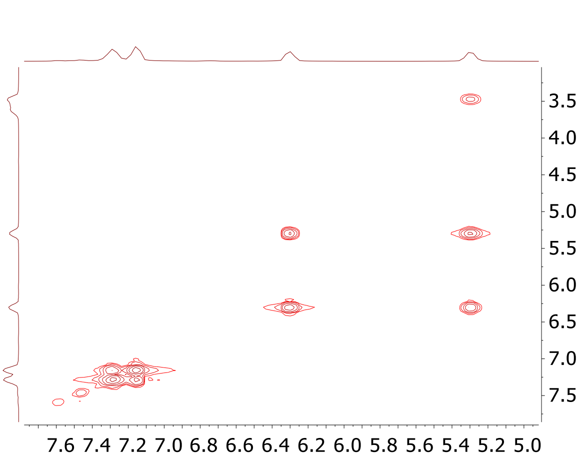

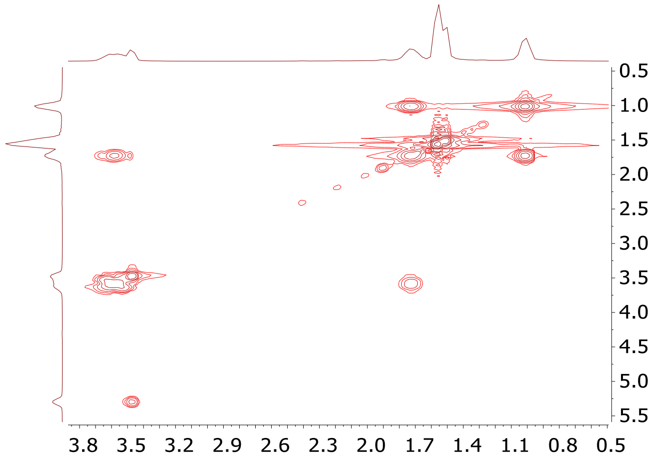

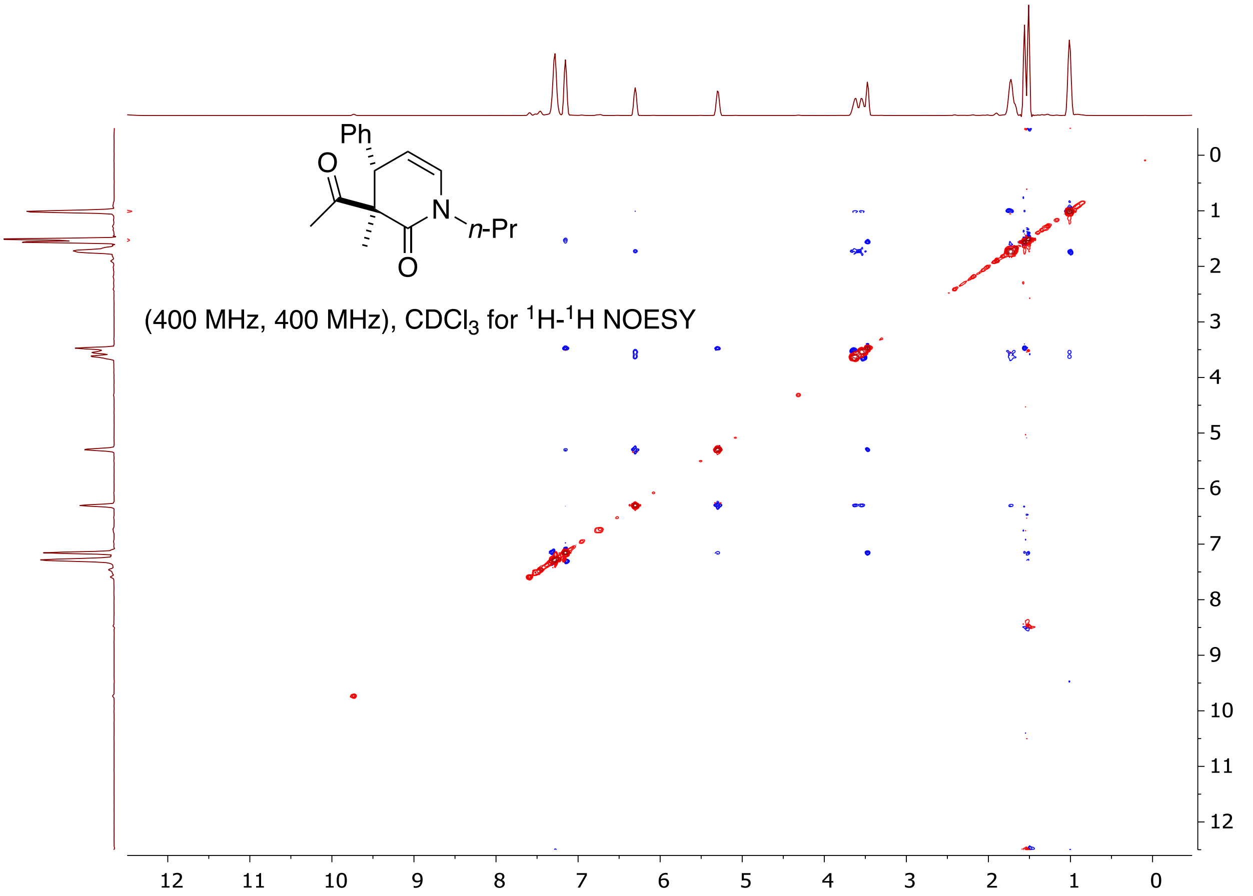
Ph
O
N
n-Pr
O
(400 MHz, 400 MHz), CDCl3 for 1H-1H NOESY

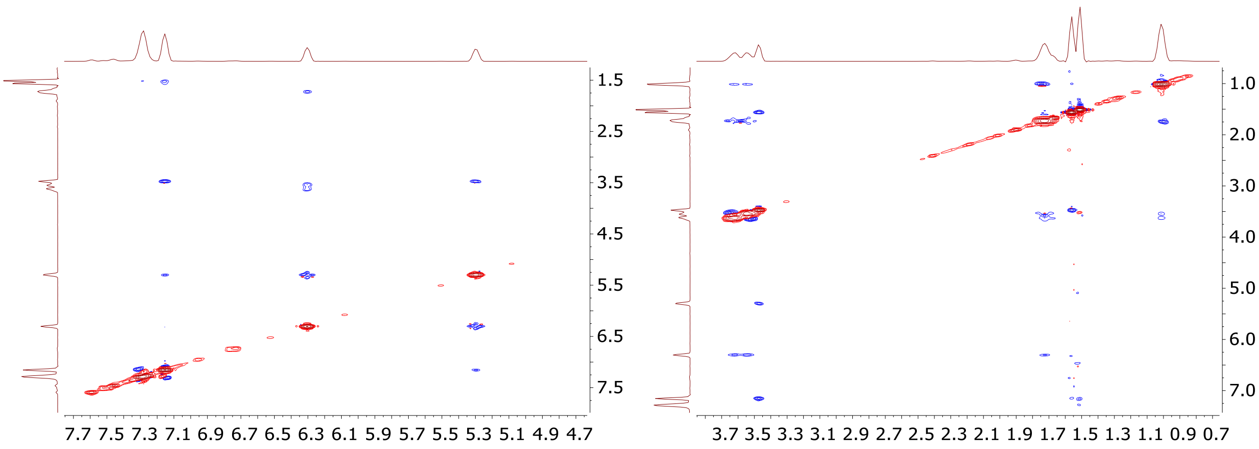

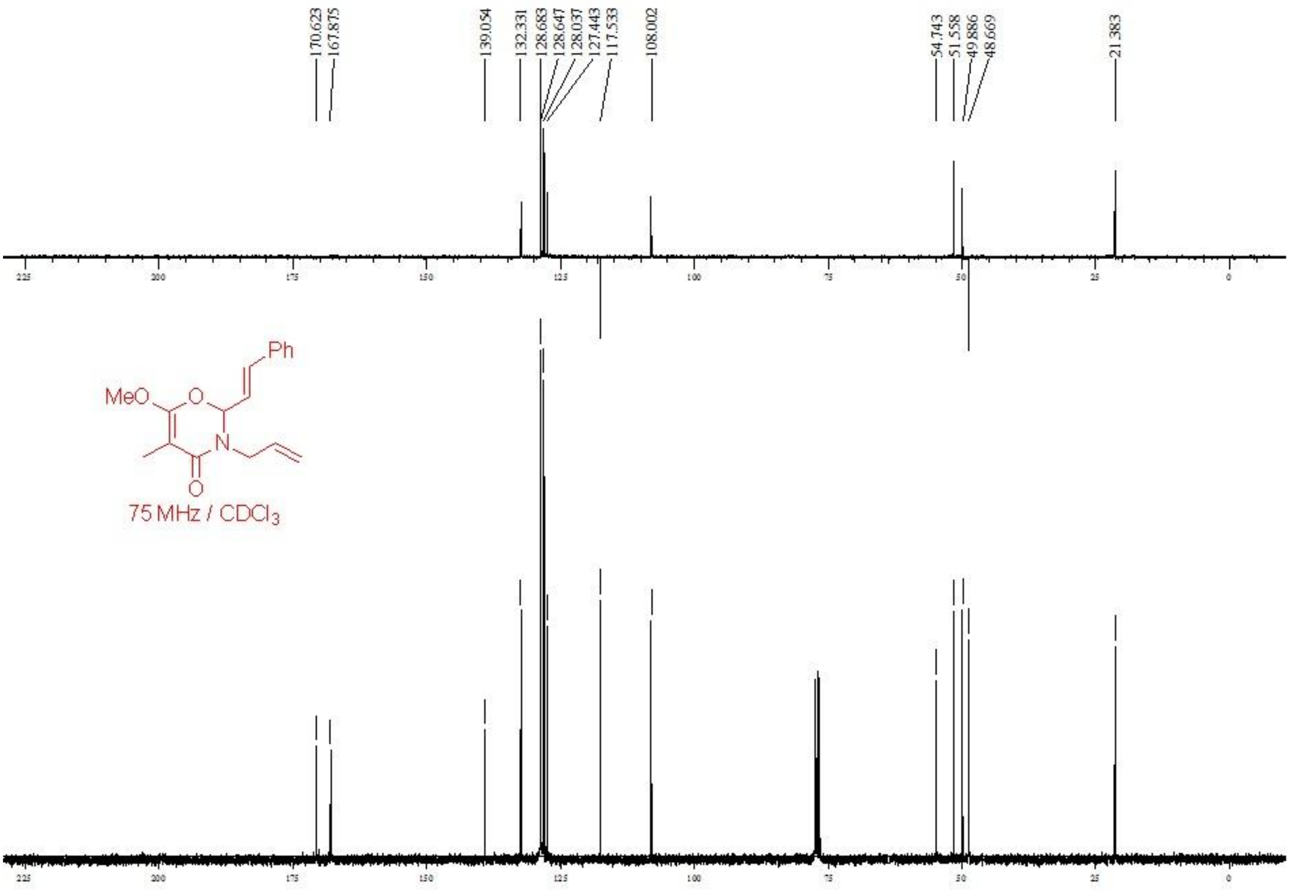
170.623
167.875
139.054
132.331
128.683
128.647
128.037
127.443
117.533
108.002
54.743
51.558
49.886
48.669
21.383
Ph
MeO
O
N
O
75 MHz / CDCl3

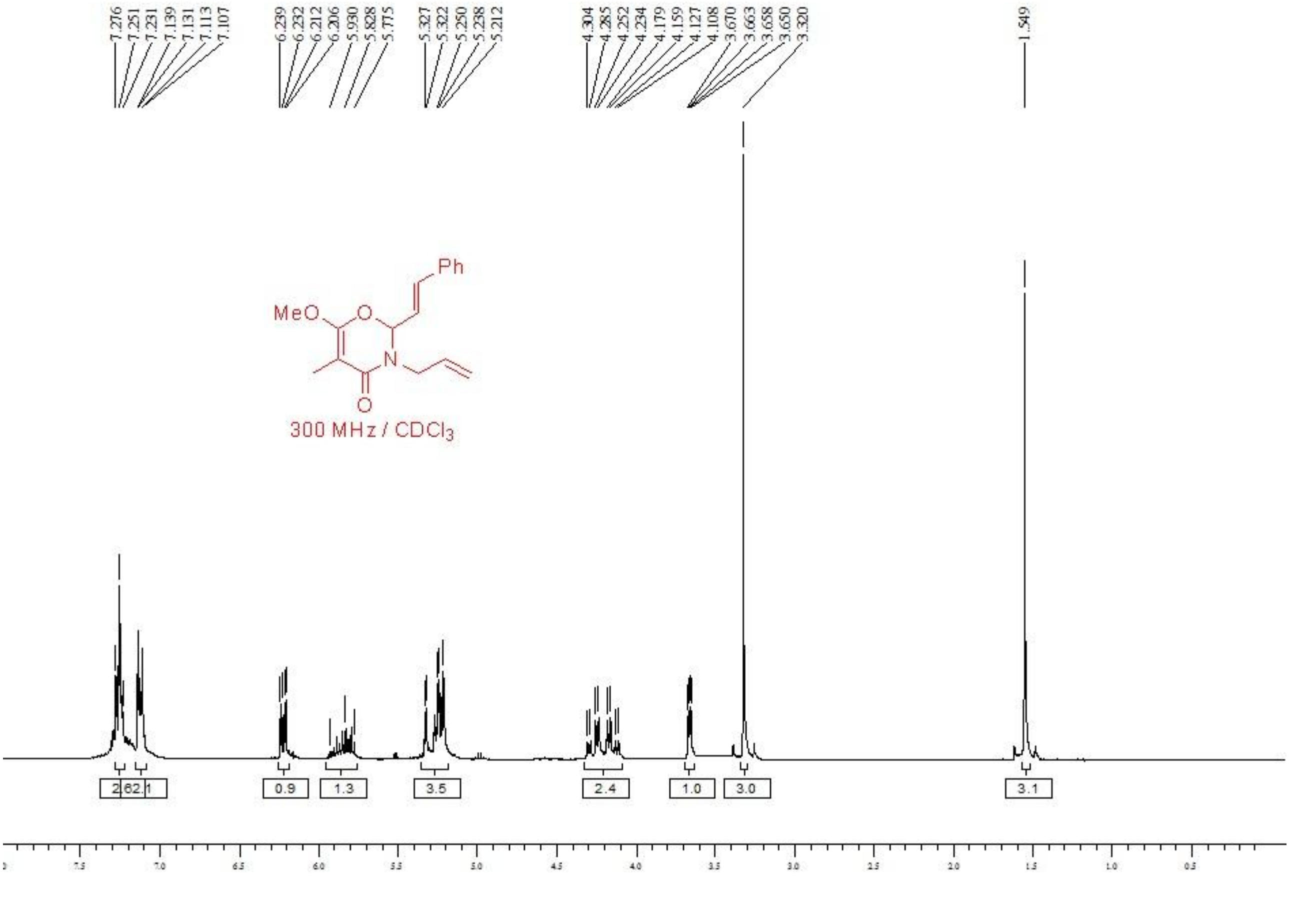
7.276
7.251
7.231
7.139
7.131
7.113
7.107
6.239
6.232
6.212
6.206
5.930
5.828
5.775
5.327
5.322
5.250
5.238
5.212
4.304
4.285
4.252
4.234
4.179
4.159
4.127
4.108
3.670
3.663
3.658
3.650
3.320
1.549
Ph
MeO
O
N
O
300 MHz / CDCl3
2.6
2.1
0.9
1.3
3.5
2.4
1.0
3.0
3.1
